# Towards defining core principles of public health emergency preparedness: scoping review and Delphi consultation among European Union country experts

**DOI:** 10.1186/s12889-020-09307-y

**Published:** 2020-10-01

**Authors:** Evelien Belfroid, Dorothee Roβkamp, Graham Fraser, Corien Swaan, Aura Timen

**Affiliations:** 1 National Institute for Public Health and the Environment (RIVM), Centre for Infectious Disease Control, Antonie van Leeuwenhoeklaan 9, 3721 MA Bilthoven, The Netherlands; 2 Health Security Consultant (formerly ECDC), Oxford, UK; 3 Athena Institute, Free University Amsterdam, De Boelelaan 1105, 1081 HV Amsterdam, The Netherlands

**Keywords:** Infectious disease, Outbreak, Public health emergencies, Preparedness, Recommendation, Consensus, Guidance

## Abstract

**Background:**

European Member States, the European Commission and its agencies work together to enhance preparedness and response for serious cross-border threats to health such as Ebola. Yet, common understanding of public health emergency preparedness across EU/EEA countries is challenging, because preparedness is a relatively new field of activity and is inherently fraught with uncertainty. A set of practical, widely accepted and easy to use recommendations for generic preparedness that bundles the activities described in separate guidance documents supports countries in preparing for any possible health threat. The aim of this consensus procedure was to identify and seek consensus from national-level preparedness experts from EU/EEA countries on key recommendations of public health emergency preparedness.

**Methods:**

To identify key recommendations and to prioritize the recommendations we started with a literature consensus procedure, followed by a modified Delphi method for consultation of public health emergency preparedness leaders of EU/EEA countries. This consisted of six consecutive steps: a questionnaire to achieve consensus on a core set of recommendations, a face-to-face consultation, preselection of prioritized recommendations, a questionnaire to achieve consensus on the prioritized set and a face-to-face consensus meeting to further prioritize recommendations.

**Results:**

As a result, EU/EEA experts selected 149 recommendations as core preparedness principles and prioritized 42. The recommendations were grouped in the seven domains: governance (57), capacity building and maintenance (11), surveillance (19), risk-assessment (16), risk- and crisis management (35), post-event evaluation (6) and implementation of lessons learned (5).

**Conclusions:**

This prioritised set of consensus principles can provide a foundation for countries aiming to evaluate and improve their preparedness for public health emergencies. The recommendations are practical, support generic preparedness planning, and can be used by all countries irrespective of their current level of preparedness.

## Background

Cross-border outbreaks demonstrate that, in an interconnected world, all countries are potentially vulnerable [1]. The likelihood of outbreaks is exacerbated by factors such as urbanization, the rapid growth and mobility of the world population, and the speed of travel [2, 3]. COVID-19, Ebola, as well as other recent threats and outbreaks, highlighted the need for countries to better prepare for threats and outbreaks and the need for a coherent view of preparedness for international purposes. The COVID-19 pandemic shows that countries are not prepared enough for this type of situations and better preparedness is needed.

In Europe, Member States and the European Commission work together with the aim of coordinating their efforts in enhancing preparedness and response for serious cross-border threats to health [4]. Yet, common understanding of public health emergency preparedness across EU/EEA countries is challenging, because preparedness is a relatively new field of activity and is inherently subject to uncertainty [5]. In general, the evidence level supporting preparedness actions/recommendations is low and most of them are produced by consensus, case studies, or outbreak descriptions [6]. Several knowledge gaps have been identified for the preparedness evidence base [7], such as the lack of instruments for improving public health preparedness as well as instruments that can be used to measure and promote preparedness quality [8, 9].

There are a large number of guidance documents available, each describing preparedness recommendations and activities for specific situations or specific stakeholders. When countries want to prepare themselves in general for a wide range of threats it is very difficult to assimilate the generic elements that are described across these different guidance documents. A set of practical, widely accepted and easy to use recommendations for generic preparedness that integrates guidance across all aspects of the preparedness emergency cycle supports countries in preparing for any possible health threat. Until now, there is no overview of recommendations for generic preparedness planning at operational level to support countries in preparedness planning at both the local and the national level.

The aim of this consensus procedure was to seek consensus from national-level experts from EU/EEA countries on identification of core principles of public health emergency preparedness. The findings can be used in a systematic and integrated approach to public health emergency preparedness planning.

## Methods

**Fig. 1:**
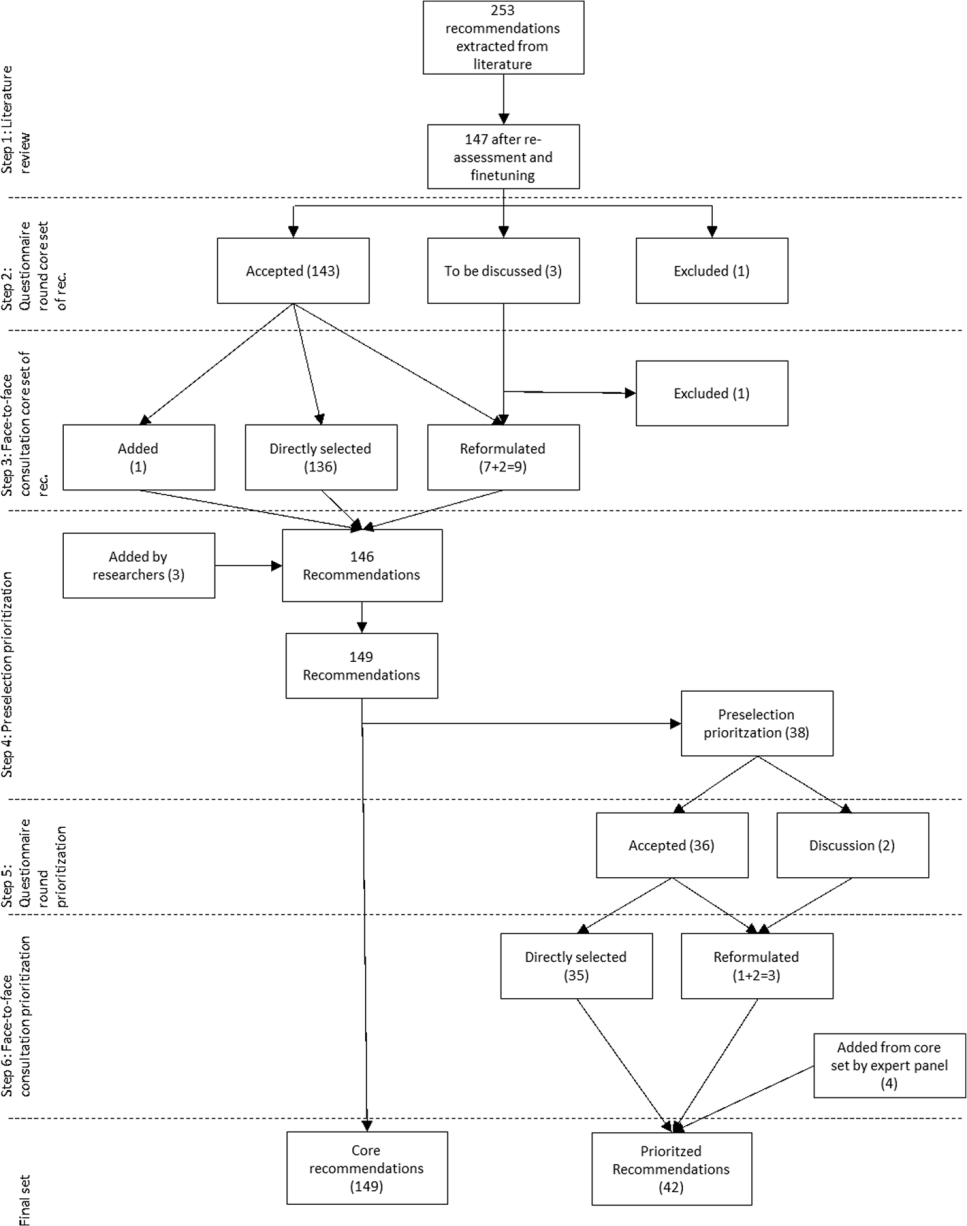
Flowchart of recommendations through all steps

A multistep approach was developed to achieve consensus on key recommendations for planning for public health emergency preparedness from a national perspective and to prioritize the recommendations (Fig. 1). A literature review was conducted, followed by a modified Delphi method [10] that consists of a series of consecutive steps: a questionnaire to achieve consensus on core set of recommendations, a face-to-face consultation, preselection of prioritized recommendations by the researchers, a questionnaire to achieve consensus on the prioritized set and a face-to-face consensus meeting on the prioritized recommendations.

### Step 1: Literature study

For the literature study grey literature was identified relating to public health emergency preparedness applicable to all European countries. As information sources the websites of the WHO and ECDC were scanned and via Google websites of other public health institutes and organizations (performed by EB and DR). Documents produced by international organizations were included, because their guidance documents apply to more than one country. Documents were selected as a source of evidence if they described public health emergency planning from a national perspective, provided guidance for preparedness, described tools or checklists to assess the level of preparedness, or described lessons learnt for emergency planning from a national perspective. In addition, country-level public health emergency preparedness plans were included that were available for review and developed after the 2009 pandemic (H1N1) influenza outbreak. Documents were systematically assessed for data items: all relevant information, recommendations or questionnaire items extracted that expressed a recommendation on preparedness from a national perspective, see Fig. 1. The extraction was done by two researchers (EB and DR) independently. After the extraction all recommendations were refined by four researchers (EB, DR, CS and AT together) to make them generically applicable. This means that disease specific recommendations were altered textually to make them applicable for a wide range of infectious diseases, recommendations that were solely applicable in a disease specific context were excluded and recommendations that contained the same message were excluded. A common set of domains were identified in the extracted recommendations, based on the expertise of the team and the extracted recommendations. The recommendations were processed in an online questionnaire (EU Survey), see Additional File 3 for the recommendations included in the questionnaire and Additional file 1 (Questionnaire core set of recommendations) for the questionnaire.

### Step 2: Questionnaire core set of recommendations

In step 2 we presented the preselected recommendation to a panel by means of a digital questionnaire. We invited the National Focal Points for Preparedness and Response (NFP P&R) of 31 EU/EEA countries, their alternates, or another expert from their country with at least 3 years of experience in preparedness planning for our panel. The NFPs P&R are experts designated by their member states to represent them in international meetings. We aimed for one response per country. The invitations were sent by ECDC. Non-responders received at least one reminder.

The expert panel was asked to appraise the relevance of each of the recommendations in an online survey on a 9-point Likert Scale (1 = not relevant, 9 = very relevant). Additionally, an open textbox was provided for any comments or adding new recommendations, for each main section of the questionnaire.

#### Analysis

Median relevance scores were calculated for each recommendation. Recommendations with a median score > 7 and > 70% of the scores in the highest tertile indicated that the recommendation were regarded as accepted by the panel, whereas recommendations with a median score > 7 and < 70% of the scores in the highest tertile were identified as requiring further consultation. Recommendations with a median score of 7 and > 70% of the scores in the highest tertile were labelled to be discussed by the research team, whereas recommendations with the score of 7 and < 70% of the scores in the highest tertile were excluded [10]. Finally, a recommendation with a median score < 7 indicated that the recommendation was rejected by the expert panel. This analysis is derived from the RAND/UCLA Appropriateness Method User’s Manual [10]. In our analysis we use a median that is more strict then in the RAND manual to ensure we only select recommendations that are valued highly by the entire group of experts.

If in the open text box comments were added concerning a recommendations, the team (EB, DR, CS, AT) assessed the comments and a decision was made whether panel consultation was needed on that recommendation even if the analysis of the scores concluded otherwise.

### Step 3: Face-to-face consultation core set of recommendations

In step 3 we conducted a face-to-face meeting to achieve consensus on the recommendations that were identified as requiring further consultation. For the face-to-face consultation, we sent invitations to the same experts as invited for the questionnaire. It was possible for countries to send a delegate if necessary. The 2-day meeting was organized on the 10th and 11th of April 2017 and was held in Utrecht (the Netherlands). Prior to the meeting all experts received a personal feedback report, which included details on the analysis of the scores, an overview of the group scores and their personal score for each recommendation. The aim of the face-to-face meeting was to discuss the recommendations that were not directly accepted or rejected by the expert panel during the online survey, in order to reach consensus on a complete set of recommendations. After the plenary discussion the experts voted for the in- or exclusion of recommendations needing further consultation, recommendations with suggested textual adjustments, as well as proposals of new recommendations. We used a threshold of 70% for acceptance. Furthermore, criteria for the preselection of priority recommendations were discussed with the expert group. After the set of recommendations was selected, the set was assessed by the research team and completed where necessary.

### Step 4: Preselection prioritization

In step 4, 5 and 6 we aimed to prioritize the selected recommendations, as the set resulting from step 3 could be too extensive for practical use in preparedness evaluation and planning. A preselection of the selected recommendations from step 3 was done by five experts (EB, DR, CS, AT, AJ). The preselection was performed based on the following criteria: the recommendation is essential for PHEP, the recommendation is feasible and accessible for all EU countries, the recommendation functions as enabler for other recommendations. All recommendations had to meet all criteria to be preselected. The experts discussed all recommendations and determined per recommendation whether the recommendation met the criteria. The preselected recommendations served as input for step 5.

### Step 5: Questionnaire prioritization

We invited the experts as in step 2 for the panel. The expert group was invited per e-mail by the ECDC. Non-responders received at least one reminder. See Additional file 2 (Questionnaire baseline set) for the questionnaire.

We asked the experts in an online questionnaire (EU Survey) to indicate on a 9-point Likert scale (1 = not appropriate, 9 = very appropriate) the appropriateness of the preselected recommendations as a baseline set needed to achieve preparedness, applicable for all countries. There was the option for ‘no opinion’ and an open text box for comments to add recommendations. The questionnaire was structured according to the seven domains.

#### Analysis

The analysis for step 4 was the same as the analysis for step 2.

### Step 6: Face-to-face consultation prioritization

The face-to-face expert meeting was organized to discuss the recommendations that were not directly accepted or rejected as a prioritized recommendation, or were added by the expert panel during the online survey. The purpose of the discussion was to achieve consensus on the prioritized set of recommendations. After the plenary discussion the experts voted for the in- or exclusion of recommendations needing further consultation, recommendations with suggested textual adjustments, as well as proposals of new recommendations. We used a threshold of 70% for acceptance. The meeting took place during a regular NFP P&R meeting in Stockholm, organized by the ECDC on May 18th, 2017_._

## Results

### Step 1: Literature review

**Fig. 2:**
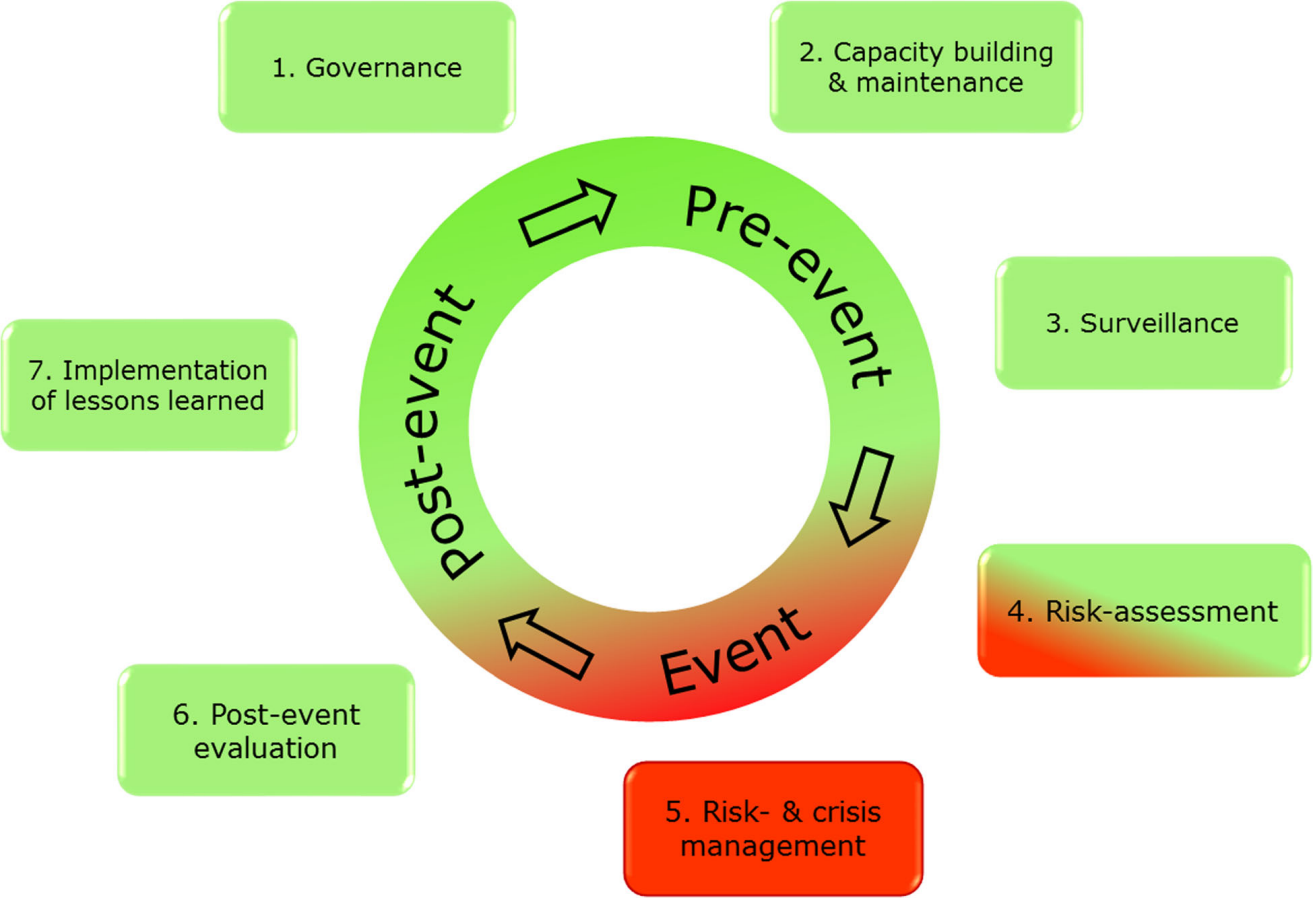
Public Health Emergency Preparedness cycle

In total, the search identified twenty documents of grey literature (sources of evidence). The documents were characterised as follows: six were ECDC guidance documents, nine were WHO documents and one was developed by CDC and one by UNISDR. Three EU member state preparedness plans were identified that met our inclusion criteria, see Table 1. The 20 documents (sources of evidence) resulted in 253 extracted recommendations, see Fig. 1. All extracted recommendations were made generic by the researchers so they apply to any disease or country. This synthesis of results led to 147 recommendations to be included in the questionnaire, see Additional file 3. The recommendations were grouped in the following domains: governance, capacity building and maintenance, surveillance, risk-assessment, risk- and crisis management, post-event evaluation and implementation of lessons learned, see Fig. 2. The domain risk- and crisis management also includes risk communication.

**Table 1:** Included literature

Published by	Title	Year	Reference
ECDC	Handbook on simulation exercises in EU public health settings - How to develop simulation exercises within the framework of public health response to communicable diseases	2014	https://ecdc.europa.eu/sites/portal/files/media/en/publications/Publications/Simulation-exercise-manual.pdf
ECDC	Preparedness planning for respiratory viruses in EU Member States - Three case studies on MERS preparedness in the EU	2015	https://ecdc.europa.eu/sites/portal/files/media/en/publications/Publications/Preparedness%20planning%20against%20respiratory%20viruses%20-%20final.pdf
ECDC	Ebola emergency preparedness in EU Member States – Conclusions from peer-review visits to Belgium, Portugal and Romania	2015	https://ecdc.europa.eu/en/publications-data/ebola-emergency-preparedness-eu-member-states-conclusions-peer-review-visits
ECDC	Assessing communicable disease control and prevention in EU enlargement countries - Disease surveillance, preparedness and response, health governance and public health capacity development	2016	https://ecdc.europa.eu/sites/portal/files/media/en/publications/Publications/communicable-disease-control-assessment-EU-enlargement-countries.pdf
ECDC	Handbook on using the ECDC preparedness checklist tool to strengthen preparedness against communicable disease outbreaks at migrant reception/detention centres	2016	https://ecdc.europa.eu/en/publications-data/handbook-using-ecdc-preparedness-checklist-tool-strengthen-preparedness-against
ECDC	Zika virus disease epidemic: Preparedness planning guide for diseases transmitted by *Aedes aegypti* and *Aedes albopictus*	2016	Zika virus disease epidemic: Preparedness planning guide for diseases transmitted by Aedes aegypti and Aedes albopictus
WHO	Development, monitoring and evaluation of functional core capacity for implementing the International Health Regulations – Concept note	2005	https://www.who.int/ihr/publications/concept_note_201507/en/
WHO	Checklist and Indicators for Monitoring Progress in the Development of IHR Core Capacities in States Parties	2013	https://www.who.int/ihr/checklist/en/
WHO	Joint External Evaluation Tool: International Health Regulations (2005)	2016	https://apps.who.int/iris/handle/10665/204368
WHO	Ebola and Marburg virus disease epidemics: preparedness, alert, control and evaluation – Ebola Strategy	2014	https://www.who.int/csr/disease/ebola/manual_EVD/en/
WHO	Ebola Virus Disease – Consolidated Preparedness Checklist	2015	https://www.who.int/csr/resources/publications/ebola/ebola-preparedness-checklist/en/
WHO	Recommendations for Good Practice in Pandemic Preparedness - identified through evaluation of the response to pandemic (H1N1) 2009	2010	http://www.euro.who.int/en/health-topics/communicable-diseases/influenza/publications/2010/recommendations-for-good-practice-in-pandemic-preparedness-identified-through-evaluation-of-the-response-to-pandemic-h1n1-2009
WHO	Key changes to pandemic plans by Member States of the WHO European Region based on lessons learnt from the 2009 pandemic	2012	http://www.euro.who.int/en/health-topics/communicable-diseases/influenza/publications/2012/key-changes-to-pandemic-plans-by-member-states-of-the-who-european-region-based-on-lessons-learnt-from-the-2009-pandemic
WHO	Joint European Pandemic Preparedness Self-Assessment Indicators	2010	https://ecdc.europa.eu/sites/portal/files/media/en/publications/Publications/100326_Joint_European_Pandemic_Indicators.pdf
WHO	Pandemic Influenza Risk Management – WHO Interim Guidance	2013	https://www.who.int/influenza/preparedness/pandemic/influenza_risk_management/en/
CDC	Public Health Preparedness Capabilities – National Standards for State and Local Planning	2011	https://www.cdc.gov/cpr/readiness/00_docs/DSLR_capabilities_July.pdf
UNISDR	Developing Early Warning Systems: A Checklist	2006	https://www.unisdr.org/we/inform/publications/608
SGDSN	France – National Influenza Pandemic and Response Plan	2011	https://ecdc.europa.eu/en/seasonal-influenza/preparedness/influenza-pandemic-preparedness-plans
Unknown	Italy – National Plan for Preparedness and Response to an Influenza Pandemic	2010	https://ecdc.europa.eu/en/seasonal-influenza/preparedness/influenza-pandemic-preparedness-plans
DH Pandemic Influenza Preparedness team	UK – UK Influenza Pandemic Preparedness Strategy	2011	https://ecdc.europa.eu/en/seasonal-influenza/preparedness/influenza-pandemic-preparedness-plans

The seven domains are grouped into three phases: The pre-event phase spans all activities related to planning and anticipation, whereas the event phase focuses on the execution of existing preparedness plans in response to a (potential) public health threat. The post-event phase takes place after the recovery from a public health threat and emphasises the continuous improvement of all domains and elements represented in the PHEP process.

### Step 2: questionnaire core set of recommendations

Out of the 31 invited countries, 27 responded to the questionnaire. Two countries had two experts filling out the questionnaire from local and national level. Because every response was valued equally, we could only include one response per country to have a balanced panel. Therefore, we used the response of the respondent with national expertise and excluded the other responses. The represented countries are described in Table 2. The years of experience with preparedness planning ranged from 2 to 34 years. The experts worked at the national public health institute or at the Ministry of Health.

**Table 2:** Expert panels

	Panel Step 2	Panel Step 3	Panel Step 5
Austria	1	1	1
Belgium	1	1	1
Bulgaria	1		
Croatia			
Cyprus	1		
Czech Republic			1
Denmark	1		1
Estonia	1	1	1
Finland	1		1
France			1
Germany	1	1	1
Greece	1		1
Hungary			1
Iceland	1		1
Ireland	1		1
Italy	1	1	1
Latvia	1	1	
Liechtenstein	1		
Lithuania	1		1
Luxembourg	1		
Malta	1	1	1
Netherlands	1		1
Norway	1	1	
Poland	1	1	1
Portugal	1		1
Romania	1	1	1
Slovakia	1		
Slovenia	1	1	1
Spain	1	1	1
Sweden	1		1
United Kingdom	1		1
Other expert not representing a country		2	
Total	27	12 (14)	23

The expert group directly accepted 143 recommendations, one recommendation was rejected and three recommendations needed further consultation (see Additional File 3). The analysis of the open text boxes responses indicated that seven recommendations required textual adjustment. One new recommendation was suggested by the experts.

### Step 3: Face-to-face meeting core set of recommendations

Twelve country experts and two experts not representing a country attended the meeting, see Table 2. The two experts not representing a country attended the meeting because of their professional interest. Experts were able to discuss in plenary and vote for the inclusion and exclusion of recommendations. These included recommendations needing further consultation (resulting from the statistical analysis), recommendations with suggested textual adjustments, as well as proposals of new recommendations, based on the comments provided in the open text boxes.

In total, eleven recommendations were discussed in the face-to-face meeting (three needing further consultation, one new added recommendation and seven proposed textual adjustments). Out of the eleven recommendations discussed in the face-to-face meeting, eight were textually adjusted by the experts, two were rejected and one was added. Three recommendations were added by the researchers because we identified elements were missing when evaluating the set. In total, 149 recommendations were included in the core set of recommendations, see Table 3.

**Table 3:** Core set of recommendations (149 recommendations)

**Governance**	
1. Emergency preparedness should be integrated in national health strategies, financing and plans^d^.	
2. Multi-sectoral emergency risk management policies and legislation include public health treats^d^	
3. A national Public Health Emergency Preparedness Plan should be developed, kept updated or endorsed by e.g. National Competent Body.^a^	
4. The national Public Health Emergency Preparedness Plan should be implemented.	
5. Preparedness planning should include a self-assessment, involving identification of gaps and possible solutions, human resources capacity, relevant national stakeholders.	
6. This self-assessment should be integrated into the existing strategic, planning and financial mechanism.	
7. Preparedness planning should include assessing and strengthening existing capacities (structures/services, staff equipment, written plans for preparedness, standard operating procedures).	
8. Preparedness planning should include development of appropriate national stockpiles.	
9. Preparedness planning should include identification of suppliers for medical countermeasures, including delivery capacity and time.	
10. Preparedness planning should include the capacity to support operations at the intermediate and community/primary response levels during a public health emergency.	
11. Preparedness planning should include community preparedness to prepare for, resist, and recover from public health incidents.	
12. Preparedness should include: the capacity to prevent, detect and manage outbreaks, during large sudden influxes of migrants.	
13. Preparedness plans should be flexible and easy adaptable.	
14. Preparedness planning should ensure cross-sectorial collaboration and clearly defined roles and responsibilities for all stakeholders.	
15. Whole-of-government (i.e. formal and informal networks) biosafety and biosecurity system should be in place for human, animal, and agriculture facilities.	
16. Multi-sectorial and multi-stakeholder coordination, command and control should be based on established infrastructure and should be continually strengthened during the planning process.	
17. Priority public health risks and resources should be mapped and utilized.	
18. Countries should have public health, medical, and mental/behavioural health systems that support recovery.	
19. Preparedness plans for events of biological hazards should be in place jointly developed by the public health and non-health sectors such as civil protection, border control and customs.	
20. A specific national framework should be in place for priority threats (such as pandemic Influenza) across all sectors.	
21. Regarding pandemic preparedness, strong cross-government planning and coordination remains critical and should be led by the Department of Health.	
22. The pandemic plans should be consistent with international (e.g. WHO and EU) available guidance.	
23. Safety measures for the handling of pathogenic substances should be in place and known by health care workers.	
24. Infection prevention and control standards should be established and functioning at national and hospital levels.	
25. Antimicrobial stewardship (set of coordinated strategies to improve the use of antimicrobial medications) should be implemented.	
26. Laboratory services should be available to test for priority health threats.	
27. Laboratory biosafety and laboratory biosecurity (Biorisk management) practices should be in place and implemented.	
28. Preparedness should involve national, regional and global networks.	
29. Collaboration between countries should be in place to maintain high levels of preparedness.	
30. The preparedness and response system for public health emergencies (including communicable diseases) should meet EU best practices.	
31. National IHR Focal Points functions and operations should be in place as defined by the IHR (2005).	
32. IHR obligations regarding Points of Entry should be fulfilled.	
33. Preparedness should be independently evaluated, facilitated by the WHO.	
34. Preparedness plans should include a capacity building strategy.	
35. Availability of a competent public health workforce for a continuum of health services should be ensured.	
36. Human resources should be available to implement IHR core capacity requirements.	
37. For respondents that are assisting in a public health emergency abroad, a protocol should be in place for medical evacuation.^a^	
38. Public Health authorities (i.e. decision-makers) should establish communication policies and procedures to develop, coordinate, and disseminate information related to an event of public health concern.	
39. The communication strategy should ensure timely and effective communication before and during an event.	
40. The communication strategy should include a scale-up approach.	
41. Emergency communication plans should remain flexible and updated as needed.	
42. Emergency communication plans should be pragmatic and straightforward to implement.	
43. Emergency communications plans should be tested.	
44. Emergency communication plans should cover the possibility that certain events receive increased media attention.	
45. Emergency communication plans should cover the possibility that certain events lead to a higher demand from the public for information.	
46. Information related to an event should be disseminated to the public, in order to explain the outbreak, to establish confidence and to minimize the risk of infection.	
47. Communication to the public should be harmonized with other national and international organizations.	
48. Public Health authorities should create key messages for public communication.	
49. Information to the public should be meaningful, relevant and timely.	
50. Information to the public should be open and transparent.	
51. Information to the public should take into account risk perceptions of the public.^a^	
52. Communication to the public should take into account characteristics of the population such as language, social, religious, cultural, political and/or economic aspects.	
53. Public Health authorities should set up multiple risk communication channels (e.g. website, E-mail, subject-specific telephone lines).	
54. Public Health authorities should provide timely information and guidance about an event to health and other professionals, so they can appropriately respond to the public.	
55. Public Health authorities should prepare ad hoc information material for different stakeholders (e.g. simplified case definitions for community use).	
56. Public Health institutions should address ethical issues and specific needs of vulnerable populations (e.g. children, pregnant women, elderly people, malnourished people, people who are ill or immunocompromised, and migrants and refugees) in their preparedness plan.^a^	
57. Public Health organizations should counter misinformation and prevent stigma, even among educated hospital staff.	
**Capacity building & maintenance (Education, training & simulation exercise)**	
1. Skills and competences of public health personnel should be strengthened to sustain public health surveillance and response at all levels of the health system.	
2. Education, training and exercises should be part of an organization’s preparedness planning activities.	
3. Education, training and exercises should be supported at the strategic and operational level of an organization.	
4. Public Health authorities should assess the level of preparedness through simulation exercises.	
5. Relevant partner organizations should be involved in exercises to improve understanding of each other’s response plans.	
6. Simulation exercises should be performed to test procedures for the management of an event (e.g. key roles and decision-making).	
7. Exercises should be based on a scenario and tailored to the setting (e.g. local, regional, national, and international).	
8. In order to carry out a successful simulation exercise, the planning group should be granted a clear mandate and the authority to plan, conduct and evaluate the exercise.	
9. The purpose of a simulation exercise should be to identify areas for improvement.	
10. Initial aims and objectives of education, training, and exercises should be evaluated and lessons learned documented in a report.	
11. Public Health authorities should conduct exercises to test the actual functionality of IHR core capacities.^a^	
**Surveillance**	
1. Public Health authorities should have an indicator-based surveillance system in place (e.g. syndromic surveillance or mortality surveillance).	
2. These indicators should be defined in protocols to enable timely follow-up.	
3. Public Health authorities should have an event-based surveillance system in place (e.g. media surveillance).	
4. These events should be defined in protocols, to enable timely follow-up.	
5. Public Health authorities should participate in EU surveillance networks.	
6. The surveillance system should meet EU & WHO standards with regard to epidemiological data on all diseases under EU surveillance, their case definitions, and reporting protocols.	
7. The surveillance system should provide real-time reporting of surveillance data	
8. The surveillance system should generate an early warning signal of a possible event of public health concern.	
9. The surveillance system should be sensitive and flexible, to detect initial cases or events.	
10. The surveillance system should obtain information from a broad range of different and reliable resources.^a^	
11. The surveillance system should be able to provide the information necessary to inform and advice response.	
12. The surveillance network should include information from veterinary surveillance systems.	
13. The surveillance network should include information from entomological surveillance systems.	
14. The surveillance network should include information from environmental surveillance systems.	
15. The surveillance network should include information from meteorological surveillance systems.	
16. The surveillance network should include information from microbiological surveillance systems.	
17. All relevant surveillance systems should be integrated in a network that consistently exchanges information.	
18. Surveillance data should be systematically and regularly reported to the relevant sectors and stakeholders.	
19. Public Health authorities should have reporting networks and protocols in place.	
**Risk assessment**	
1. Alerts and early warnings should be assessed based on a joint analysis of the surveillance data.	
2. A risk assessment team should be assembled to assess the risks of a (possible) event of Public Health concern.	
3. The risk assessment team should include additional expertise (e.g. toxicology, animal health, food safety, etc.).	
4. Risk assessment should be used to aid preparedness planning of response activities.	
5. Clearly defined questions should be used as part of the risk assessment to help identify priority activities.	
6. Risk assessment should be used to identify risk areas.	
7. Risk assessment should be used to identify risk populations.	
8. Risk assessment should be used to identify and engage operational partners.	
9. Risk assessment should be used to identify and engage key policy partners.	
10. The level of risk assigned to an event should be based on the suspected (or known) hazard.	
11. The level of risk assigned to an event should be based on the possible exposure to the hazard.	
12. The level of risk assigned to an event should be based on the context in which the event is occurring.	
13. The level of risk assigned should be based on the disease characteristics (such as number of cases/deaths, proportion of severe disease in population, clinical groups most affected, etc.).	
14. The level of risk assigned should be based on the service capacity (e.g. number of patience presented at primary care services/admitted to hospital and intensive care specialist treatment).	
15. Risk characterization should incorporate information from quantitative model, if available and accessible, and on the expert opinion.	
16. Based on the disease characteristics, the risk assessment team should decide how frequently the risk assessment should be updated.	
**Risk and crisis management**	
1. Specific procedures should be in place for activation and deactivation (‘stand-down’) of the health emergency response.^d^	
2. An emergency operational program should be in place involving an Emergency Operations Centre, Operating Procedures and Plans, and the capacity to activate emergency operations.	
3. Countries should have a tested command and control structure with clear roles and responsibilities.	
4. Procedures for coordination of multi-sectorial activities between the ministries and sectors should be established.	
5. Coordination, command and control should be based on established infrastructure.	
6. Coordination, command and control should be continually strengthened.	
7. Procedures to coordinate all relevant partners of the health system should be established e.g. public health, medical, and mental/behavioural health services.	
8. Coordination should involve population-based care, resource mobilization, activation of support networks, advisory groups, partner networks and communication.	
9. Multidisciplinary and multisectorial Rapid Response Teams (RRT) should be established and available 24 h a day, 7 days a week.	
10. Public health system should be supported by crisis management teams on all levels.	
11. Case management procedures are implemented for IHR relevant hazards.	
12. Response decisions should take into account the following principles: precaution, proportionality and flexibility.	
13. Procedures for medical countermeasures, including implementation and dispensing, should be in place.	
14. Procedures should be in place for sending and receiving medical countermeasures during a public health emergency.	
15. Procedures for responding to foodborne disease and food contamination should be established and functional.	
16. Procedures for responding to zoonosis and potential zoonosis should be established and functional.	
17. In areas receptive for arbovirus transmission, standard operation procedures for field investigations and rapid vector control measures should be developed.	
18. Effective Public Health Response at Points of Entry, according to IHR, should be established.	
19. Public Health authorities should reinforce health monitoring systems.	
20. During the event, Public Health authorities should frequently evaluate health monitoring data related to the event.	
21. Health monitoring systems should monitor the evolving event (e.g. geographical and/or temporal distribution).	
22. Health monitoring systems should monitor the functioning of essential services.	
23. Health monitoring systems should be linked to laboratories and health facilities.	
24. Based on the gathered data, the effectiveness of response activities should be frequently evaluated.	
25. Response activities should constantly be adapted to the new situation.	
26. Information of the evolving event should be communicated to the relevant stakeholders and the public.	
27. Public Health authorities should identify, map and monitor critical communication networks.	
28. Public Health authorities should develop a comprehensive communication strategy to engage with all relevant stakeholders such as public health professionals, media and public, non-health sectors, etc.	
29. Chains of responsibility should be clearly identified to ensure effective communications within the national and international level.	
30. All relevant stakeholders should be engaged and well informed in advance, throughout and after an event.	
31. During an event, core messages given out by the different authorities need to be coordinated and standardized.	
32. During an event, consistent messages should be disseminated by a trusted authority.	
33. Information related to an event should be disseminated between all relevant stakeholders within the health sector.	
34. Information related to an event should be disseminated between all relevant stakeholders within non-health sectors.	
35. The expected behavioural response (e.g. levels of concern experienced by the population) should be taken into account in the decision process of the risk management. ^c^	
**Post-event evaluation**	
1. Public Health authorities should assess the level of preparedness by evaluating events of public health concern.	
2. Post-event evaluations should be part of an organization’s preparedness planning activities.	
3. The post-event evaluation should be conducted as soon as possible after the event.	
4. The post-event evaluation should be of qualitative nature.	
5. Post-event evaluations should consist of an internal audit, involving all national stakeholders responsible for essential public health functions.	
6. Lessons learned from all relevant sectors should be systematically recorded in a post-event report.	
**Implementation of lessons learned**	
1. Experiences and lessons learned, coming forth from post-event evaluation or exercises, should be reviewed across all relevant sectors.	
2. Experiences and lessons learned, coming forth from post-event evaluation or exercises, should be shared with the international community.	
3. Experiences and lessons learned, coming forth from post-event evaluation or exercises, should be used to improve preparedness and response activities.	
4. Experiences and lessons learned, coming forth from post-event evaluation or exercises, should be used to improve policies and practice.	
5. Nations are encouraged to write executive summary of evaluation report in English.^b^	

^a^The recommendation was textually adjusted by the experts

^b^Added by the experts

^c^Moved from other domain

^d^The recommendation was added by the researchers

The recommendations were grouped among seven domains: Governance (57 recommendations), Capacity building & maintenance (11 recommendations), Surveillance (19 recommendations), Risk assessment (16 recommendations), Risk and crisis management (35 recommendations), Post-event evaluation (6 recommendations) and Implementation of lessons learned (5 recommendations).

### Step 4: Preselection prioritization of recommendations

In total 38 recommendations from all domains were preselected to be included in the prioritized set of recommendations (Additional File 4).

### Step 5: Questionnaire prioritization of recommendations

Experts from 23 EU/EEA countries out of 31 completed the questionnaire, see Table 1. The experts represented the NFPs P&R. The experience of the experts ranged from < 3 years (1 expert), to > 10 years (9 experts). The experts worked at the national public health institute or at the Ministry of Health. Out of the 38 preselected recommendations, 36 were directly accepted by the expert panel, zero were rejected, and two needed further consultation, see Additional File 4. Three recommendations (added by the researchers in step 3) were assessed by experts of ten countries because they were added to the digital questionnaire when some expert already finished the questionnaire. Table 4 shows the prioritized set of recommendations. If all respondents scored the recommendation with a 7, 8 or 9 the recommendation is described as subject to ‘universal’ acceptance. If one or more respondents scored 6 or lower, the recommendation is described as subject to ‘majority’ acceptance. Given this method, there were 7 recommendations subject to universal acceptance and 36 to majority acceptance. All recommendations in Table 4 met the selection criteria.

**Table 4:** Prioritized set of recommendations

	Recommendation	Acceptance strength
**Governance**		
1	Emergency preparedness should be integrated in national health strategies, financing and plans.	Universal acceptance
2	National IHR Focal Points functions and operations should be in place as defined by the IHR (2005)	Universal acceptance
3	Public Health authorities (i.e. decision-makers) should establish communication policies and procedures to develop, coordinate, and disseminate information related to an event of public health concern.	Universal acceptance
4	Multi-sectoral emergency risk management policies and legislation include public health treats	Majority acceptance
5	A national Public Health Emergency Preparedness Plan should be developed, kept updated or endorsed by e.g. National Competent Body.	Majority acceptance
6	Preparedness planning should include a self-assessment, involving identification of gaps and possible solutions, human resources capacity, relevant national stakeholders.	Majority acceptance
7	Preparedness planning should include assessing and strengthening existing capacities (structures/services, staff equipment, written plans for preparedness, standard operating procedures).	Majority acceptance
8	Preparedness planning should include appropriate *medical countermeasures to protect the health of the Member States population*	Majority acceptance
9	Preparedness planning should ensure cross-sectorial collaboration and clearly defined roles and responsibilities for all stakeholders.	Majority acceptance
10	Priority public health risks and resources should be mapped and utilized	Majority acceptance
11	A specific national framework should be in place for priority threats (such as pandemic Influenza) across all sectors.	Majority acceptance
12	*Infection prevention and control standards should be established and functioning at national and hospital levels.*	Majority acceptance
13	Laboratory services should be available to test for priority health threats	Majority acceptance
14	Preparedness should involve national, regional and global networks	Majority acceptance
15	Collaboration between countries should be in place to maintain high levels of preparedness	Majority acceptance
16	Information related to an event should be disseminated to the public, in order to explain the outbreak, to establish confidence and to minimize the risk of infection	Majority acceptance
**Capacity building & maintenance (Education, training & simulation exercise)**		
1	Education, training and exercises should be part of an organization’s preparedness planning activities.	Universal acceptance
2	Skills and competences of public health personnel should be strengthened to sustain public health surveillance and response at all levels of the health system	Majority acceptance
3	Public Health authorities should assess the level of preparedness through simulation exercises.	Majority acceptance
4	*Training, exercises and incident reviews should be used to understand and improve risk management procedures and to strengthen capacities.*	Majority acceptance
5	Initial aims and objectives of education, training, and exercises should be evaluated and lessons learned documented in a report.	Majority acceptance
**Surveillance**		
1	Public Health authorities should have an indicator-based surveillance system in place (e.g. syndromic surveillance or mortality surveillance).	Majority acceptance
2	Public Health authorities should have *an epidemic intelligence* system in place.	Majority acceptance
3	Public Health authorities should participate in EU surveillance networks.	Majority acceptance
4	The surveillance system should meet EU & WHO standards with regard to epidemiological data on all diseases under EU surveillance, their case definitions, and reporting protocols.	Majority acceptance
5	*The surveillance system should generate an early warning signal of a possible event of public health concern.*	Majority acceptance
6	Surveillance data should be systematically and regularly reported to the relevant sectors and stakeholders	Majority acceptance
**Risk assessment**		
1	Alerts and early warnings should be assessed based on a joint analysis of the surveillance *and other available* data	Universal acceptance
2	Risk assessment should be used to aid preparedness planning of response activities.	Universal acceptance
3	A risk assessment team should be assembled to assess the risks of a (possible) event of Public Health concern.	Majority acceptance
**Risk- and crisis management**		Majority acceptance
1	An emergency operational program should be in place involving an Emergency Operations Centre, Operating Procedures and Plans, and the capacity to activate emergency operations.	Universal acceptance
2	Specific procedures should be in place for activation and deactivation (‘stand-down’) of the health emergency response.	Majority acceptance
3	Countries should have a tested command and control structure with clear roles and responsibilities.	Majority acceptance
4	Multidisciplinary and multi-sectorial *Rapid Response* should be established and available 24 h a day, 7 days a week.	Majority acceptance
5	Based on the gathered data, the effectiveness of response activities should be frequently evaluated	Majority acceptance
6	Public Health authorities should develop a comprehensive communication strategy to engage with all relevant stakeholders such as public health professionals, media and public, non-health sectors, etc.	Majority acceptance
7	During an event, consistent messages should be disseminated by a trusted authority.	Majority acceptance
**Post-event evaluation**		
1	Public Health authorities should assess the level of preparedness by evaluating events of public health concern.	Majority acceptance
2	Post-event evaluations should be part of an organization’s preparedness planning activities.	Majority acceptance
3	Lessons learned from all relevant sectors should be systematically recorded in a post-event report.	Majority acceptance
**Implementation of lessons learned**		
1	Experiences and lessons learned, coming forth from post-event evaluation or exercises, should be used to improve preparedness and response activities.	Majority acceptance
2	Experiences and lessons learned, coming forth from post-event evaluation or exercises, should be used to improve policies and practice.	Majority acceptance

The italic words were changed by the expert group. The non-bold recommendations are the ones accepted without changes

Universally = All respondents scored the recommendation as a 7, 8 or 9

Majority = The recommendations is selected according to the criteria but not all respondents scored the recommendation as a 7,8 or 9

### Step 6: Face-to-face meeting prioritization

The step 6 face-to-face meeting was a part of a regular NFP&PR meeting. Nineteen countries participated in the face-to-face meeting. The experts present were the NFP&PR members or their alternates. There were several parallel sessions and experts could choose to attend the session of their interest. In the meeting, five selected recommendations were altered textually and two recommendations were added to the prioritized set. In total, 42 recommendations were prioritized (Table 4).

## Discussion

In this consensus procedure, EU/EEA country preparedness experts reached consensus on core priority principles of public health emergency preparedness. Experts selected 149 core recommendations and prioritized 42. The recommendations selected support EU/EEA countries in preparing for public health emergencies by providing guidance across the full spectrum of preparedness, describing governance, capacity building & maintenance, surveillance, risk assessment, risk and crisis management, post-event evaluation and implementation of lessons learned.

In the selection of the core set of recommendations (step 2 and 3), practically all recommendations were accepted by the panel and only one recommendation was added. In the prioritization, experts accepted most preselected recommendations (36 out of 38). Experts could add recommendations from the core set to the prioritized set if they thought this was needed. However, the aim of this consensus procedure was to select recommendations that were accepted and applicable for the majority of countries. Differences between countries regarding current level of preparedness, available resources and healthcare organizations can influence the score a country gives for a recommendation. In the questionnaire and consensus meeting, experts had the opportunity to provide comments or suggestions to add, delete or modify the recommendations. The recommendations in our consensus procedure are formulated in a way that they can be used by all countries.

The selected recommendations in this consensus procedure are in line with public health emergency indicators developed by other organisations. Khan et al. [11] performed a Rand Modified Delphi with Canadian experts to develop indicators for public health emergency preparedness. The selected recommendations in the paper of Khan are similar to the recommendations selected in our consensus procedure. This implies that the selected recommendations in our consensus procedure are not only suitable for EU/EEA countries but may be valuable for countries worldwide. WHO developed a strategic framework including elements of preparedness on different levels [12]. Our recommendations are in line with the elements of preparedness in this framework, but provide more detail on the practical application.

In the face-to-face meetings most discussion concentrated on terminology. Terminology in public health preparedness is not always uniform and countries interpret terms differently. When aiming to achieve consensus on a set of recommendations that is applicable to multiple countries, it is very important that everyone interprets terms and concepts in the same way. Hence, discussion on terminology was not unexpected. During the face-to-face discussion the recommendations were reformulated by the participating experts in a way that the recommendation was clear to all experts and possible double interpretation unlikely. Countries can have various reporting systems, coordination structures and a different organization of healthcare. Therefore, one term can mean different things for different countries. In such a multinational context, achieving consensus requires a face-to-face meeting, to clarify recommendations and concepts behind them.

Another pattern is the content of the recommendations. The majority of the recommendations describe the domains ‘governance’, ‘surveillance’ and ‘risk assessment’. While the domains ‘post –event evaluation’ and ‘implementation of lessons learned’ contain only small amount of recommendations. In the included literature, there is a strong focus on the content based recommendations. This could be explained by the difficulty of defining recommendations on when and how to implement lessons learned. However, we believe that post-event evaluation and implementing lessons learnt are highly important for quality improvement. Training, exercises, threats and events should be used to learn valuable lessons for future outbreaks. Moreover, preparedness plans and future trainings should be updated regularly based on lessons learnt.

In this consensus procedure solely grey literature was included since the scientific evidence available on outbreak preparedness has a low evidence base, is mostly disease specific and often does not describe preparedness from a national perspective [11, 13].

In a previous literature review [14] and modified Delphi procedure [13] we systematically reviewed scientific literature and selected recommendations for generic preparedness from the perspective of first responders (among others hospitals, general practitioners, municipal health services, ambulance services). These recommendations describe a wide range of preparedness activities from developing a preparedness plan, preparing for control measures, coordination and collaboration, and evaluation. The previous study and the process described in this paper are complementary. A country that is well prepared for public health emergencies is prepared on all levels, from the local level to the national level. When assessing the level of preparedness within a country, both the set targeted at the national level and the set targeted at the first responders should be used.

Our consensus procedure has several strengths and limitations. In this consensus procedure we selected recommendations using a systematic approach. One of the strengths is that all EU/EEA countries were invited for the panels and their responses were valued equally. The value of a face-to-face meeting can be found in building a common understanding of preparedness needs and priorities among countries. The bottom-up approach of this consensus procedure contributes to a widely accepted set of recommendations and a common understanding of preparedness. In both our panels, a very high number of countries were represented (27 and 23). Although in both face-to-face meetings the number of countries represented was lower (12 and 19), both groups contained a sufficient number of participants, as compared to the recommended 7–15 participants for a Delphi panel [10].

One of the limitations is that the basis for the recommendations selected in this consensus procedure lies within grey literature because these documents were used to extract the recommendations from, and that no scientific literature was used. We did not include scientific literature because the evidence base in scientific literature regarding preparedness planning is low [11, 13]. We therefore included literature from international organizations that have a leading role in preparedness planning and are developed not by one single author but by a group of international experts.

The recommendations extracted from literature formed the basis of the first questionnaire. This could potentially have pushed the experts in a certain direction because the experts to assess a list of recommendations and not to come up with a set of recommendations from a blank page. However, experts had the opportunity in the online questionnaires and during the face-to-face consultation meetings to add, remove or adjust recommendations. After our Delphi procedure, new relevant documents were published that could not be included because the data collection was already finished. An example of these relevant documents are a report describing competencies for individuals who work in emergency preparedness [15], and the WHO framework for emergency preparedness [12]. The recommendations selected in our consensus procedure provide a detailed description of the elements of preparedness as described in the WHO strategic framework [12].

## Conclusions

The recommendations identified in our consensus procedure can be a useful guidance for preparedness planning in EU/EEA countries. This can be done in various ways, for example providing a framework for development of preparedness evaluation, and incorporating the recommendations in guidelines. A tool aiming to assess a country’s level of preparedness was developed based on the recommendations selected and prioritized in this consensus procedure [16]. The recommendations represent the criteria on which the level of preparedness is evaluated. This tool comprises the core set of recommendations and the prioritized set of recommendations. Countries can use this tool to assess their level of preparedness and identify gaps and priorities in preparedness planning. The prioritized set provides direction for the most urgent recommendations to implement.

## Supplementary information

**Additional file 1.** Questionnaire core set of recommendations. This file contains a PDF file of the questionnaire as it was send to the participating experts.

**Additional file 2.** Questionnaire baseline set. This file contains a PDF file of the questionnaire as it was send to the participating experts.

**Additional file 3:.** Core set of recommendations.

**Additional file 4:.** Baseline (prioritized) set of recommendations.

**Additional file 5:.** Interview guide step 3.

**Additional file 6:.** Interview guide step 6.

## Data Availability

The datasets used and/or analysed during the current study are available from the corresponding author on reasonable request.

## Abbreviations

EU/EEA countries: The European Union (EU), European Economic Area (EEA)
NFP P&R: The National Focal Points for Preparedness and Response
WHO: World Health Organisation
ECDC: European Centre for Disease Prevention and Control

## References

[1] WHO (2016). 2014 Ebola outbreak in West Africa - reported cases graphs.

[2] Semenza JC (2016). Determinants and drivers of infectious disease threat events in Europe. Emerg Infect Dis.

[3] Weiss RA, McMichael AJ (2004). Social and environmental risk factors in the emergence of infectious diseases. Nat Med.

[4] Decision No 1082/2013/EU of the European Parliament and of the Council of 22 October 2013 on serious cross-border threats to health and repealing Decision No 2119/98/EC Text with EEA relevance. 2013. OJ L 293, 5.11.2013, p. 1–15. (BG, ES, CS, DA, DE, ET, EL, EN, FR, HR, IT, LV, LT, HU, MT, NL, PL, PT, RO, SK, SL, FI, SV).

[5] Nelson C, Lurie N, Wasserman J (2007). Assessing public health emergency preparedness: concepts, tools, and challenges. Annu Rev Public Health.

[6] Asch SM (2005). A review of instruments assessing public health preparedness. Public Health Rep.

[7] Khan Y (2015). The evidence base of primary research in public health emergency preparedness: a scoping review and stakeholder consultation. BMC Public Health.

[8] Khan (2018). Public health emergency preparedness: a framework to promote resilience. BMC Public Health.

[9] Haeberer M, Tsolova S., Riley P, Rexroth U, Cano-Portero R, Ciotti M, Fraser G, Tools for Assessment of Country Preparedness for Public Health Emergencies: a Critical Review. Disaster Med Public Health Prep. 2020;1–11. 10.1017/dmp.2020.13. 10.1017/dmp.2020.13 PMC8532124 32366350

[10] Fitch K, Berstein SJ, Aguilar MD, Burnand B, LaCalle JR, Lazaro P, van het Loo M, McDonnell J, Vader J, Kahan JP (2001). The RAND/UCLA appropriateness method user’s manual.

[11] Khan Y, Brown AD, Gagliardi AR, O'Sullivan T, Lacarte S, Henry B (2019). Are we prepared? The development of performance indicators for public health emergency preparedness using a modified Delphi approach. PLoS One.

[12] WHO. A strategic framework for emergency preparedness. Geneva: World Health Organization; 2016. Licence: CC BY-NC-SA 3.0 IGO.

[13] Belfroid E (2017). Which recommendations are considered essential for outbreak preparedness by first responders?. BMC Infect Dis.

[14] Huis ABE, Klein Breteler J, van Steenbergen J, Hulscher M (2016). Defining and improving healthcare system's preparedness for infectious disease outbreaks: a systematic review identifying generic key recommendations and their connections to continuous quality improvement.

[15] ECDC (2017). Public health emergency preparedness – Core competencies for EU Member States.

[16] ECDC (2018). HEPSA – health emergency preparedness self-assessment tool.

