# Supplementary material for: Towards defining core principles of public health emergency preparedness: scoping review and Delphi consultation among European Union country experts
Source: BMC Public Health. 2020 Oct 1;20:1482. doi: 10.1186/s12889-020-09307-y (PMC7527265; doi:10.1186/s12889-020-09307-y)
Supplement: Supplementary file 1 — Additional file 1. Questionnaire core set of recommendations. This file contains a PDF file of the questionnaire as it was send to the participating experts. [file 12889_2020_9307_MOESM1_ESM.docx]

Strategic Planning for Public Health Emergency

Preparedness

Fields marked with * are mandatory.

**Strategic Planning for Public Health Emergency Preparedness**

Thank you very much for taking the time to fill out this questionnaire. This will take about one hour.

**Background**

The European Centre for Disease Prevention and Control (ECDC) with the collaboration of the Dutch

National Coordination Centre for Communicable Disease Control (LCI) are developing a comprehensive handbook to support EU/EEA countries through a strategic preparedness planning process. This would serve the countries in: evaluating their level of preparedness, identifying potential gaps and vulnerabilities, and strengthening their capacities when needed.

This questionnaire has been designed to ensure feedback from EU/EEA countries, in order to inform the development of the Handbook and to increase its applicability and utility. In order to accomplish this, we request that a National Focal Point for Preparedness and Response member/alternate or another expert with at least three years of experience in preparedness planning fill in this questionnaire. In the case of federal countries, an additional expert from regional/local level is encouraged to fill in the questionnaire.

Statements were extracted from a systematic review on high quality outbreak preparedness and scoping review on preparedness planning documents (including ECDC reports, other reports from international organizations and legal documents).

**Instructions**

The questionnaire consists of eight sections. Most questions are based on a 9 point Likert Scale. At the end of each section, there is also the opportunity to provide open-text comments, or to add new statements (for example: statements that are in your countries preparedness plan but not included in this questionnaire).

It is possible to save your response to the questionnaire and restart at a later moment.

We thank you very much for your time!

# 1 - General information

***Which country do you represent?**

***For which organisation do you work?**

**What is your position?**

*****

***How many years of experience do you have in preparedness planning?**

# 2 - Preparedness

## 2.1 General aspects

Please appraise the relevance of the following statements as concerns strategic planning for public health emergency preparedness.


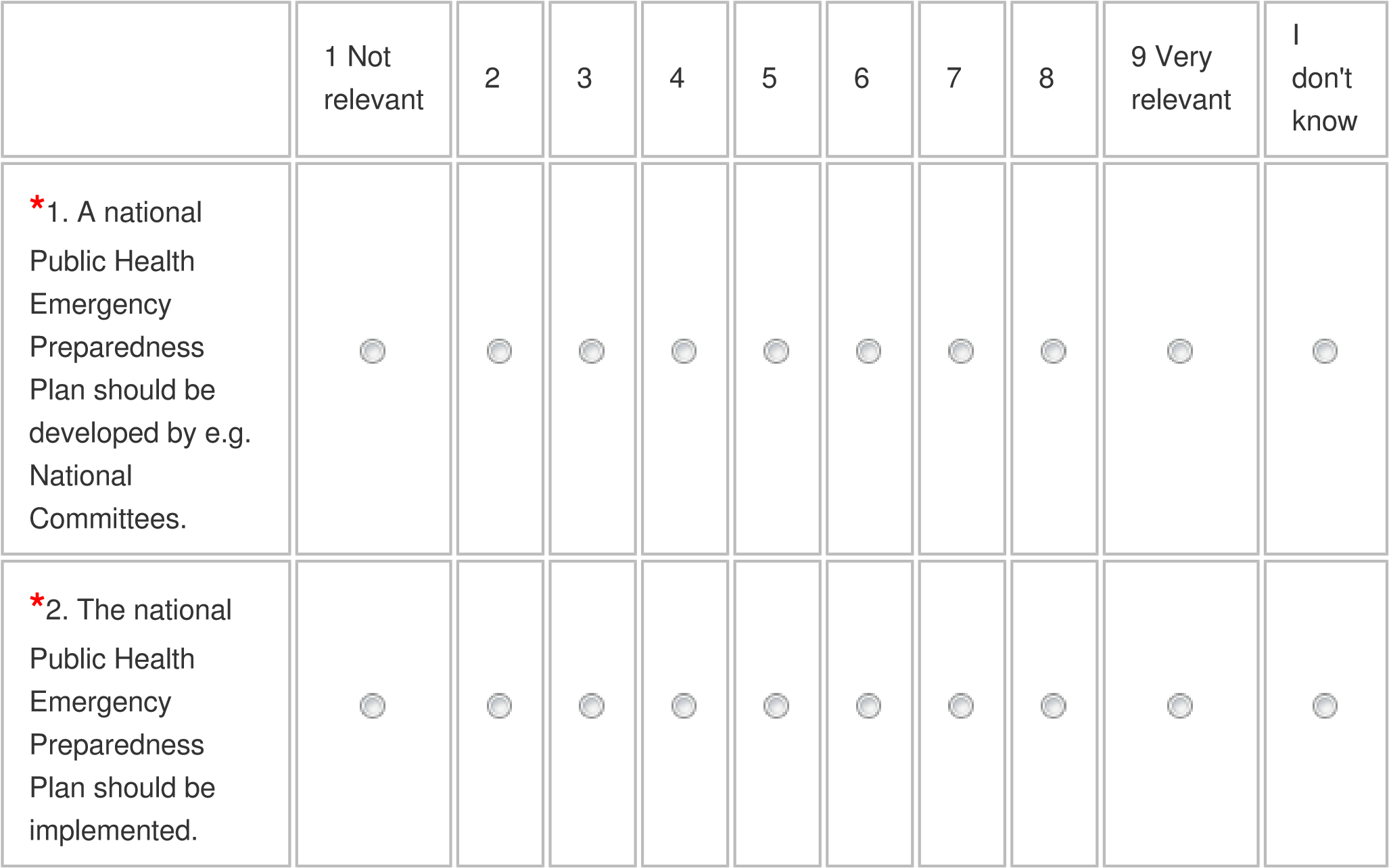


. Preparedness

3

planning should

include a self-

assessment,

involving

identification of

gaps and

possible

solutions, human

resources

capacity, relevant

national

stakeholders.


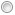

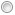

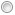

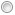

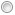

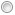

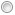

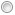

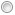

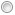


4

. This self

-

assessment

should be

integrated into

the existing

strategic,

planning and

financial

mechanism.


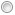

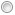

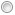

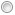

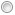

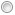

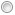

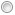

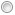

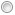


*****


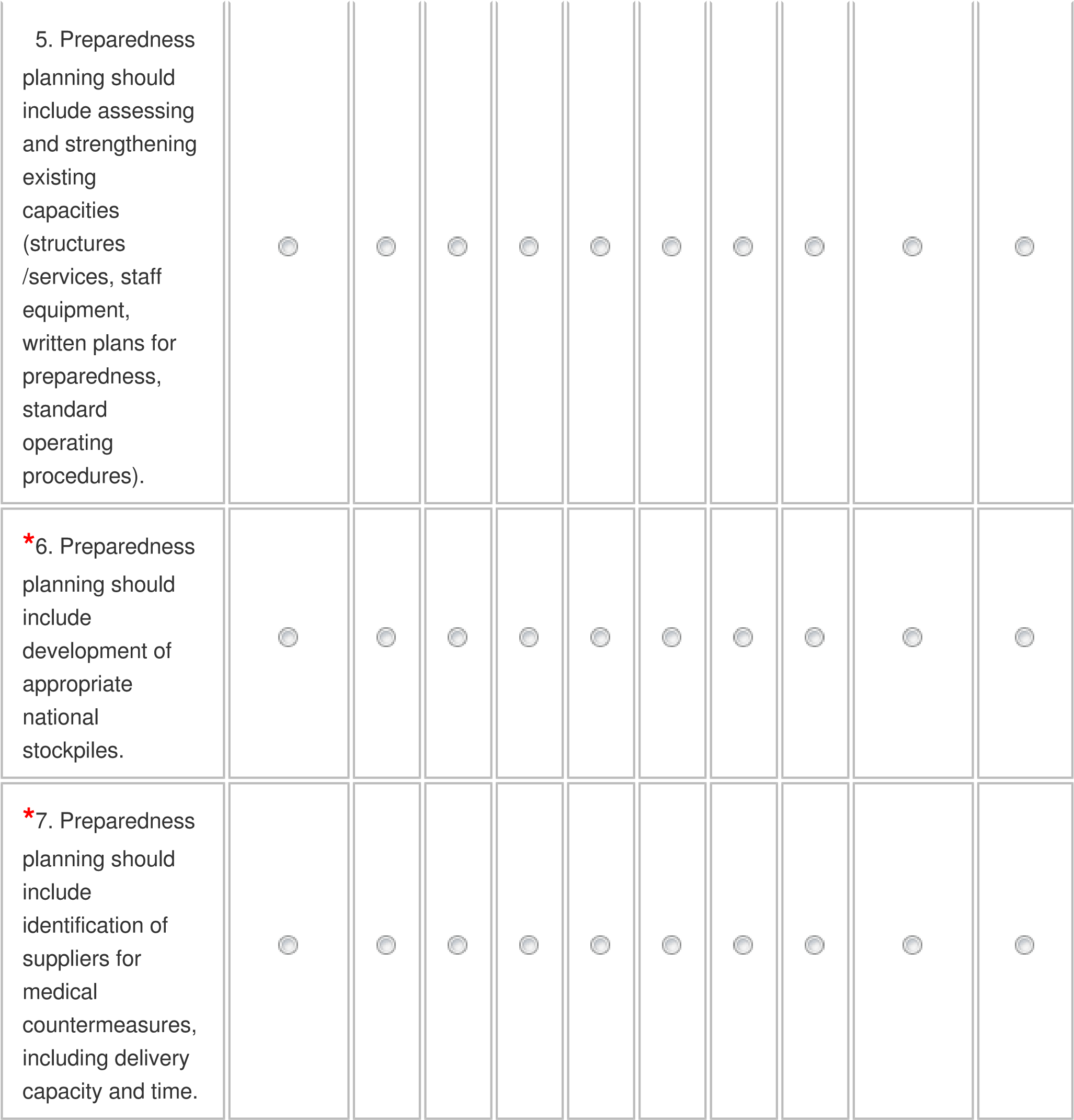


8

. Preparedness

planning should

include the

capacity to

support

operations at the

intermediate and

community

/primary response

levels during a

public health

emergency.


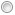

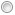

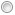

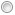

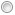

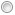

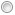

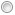

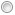

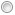


9

. Preparedness

planning should

include

community

preparedness to

prepare for,

resist, and

recover from

public health

incidents.


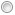

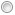

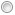

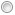

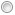

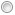

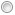

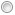

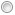

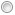


*****


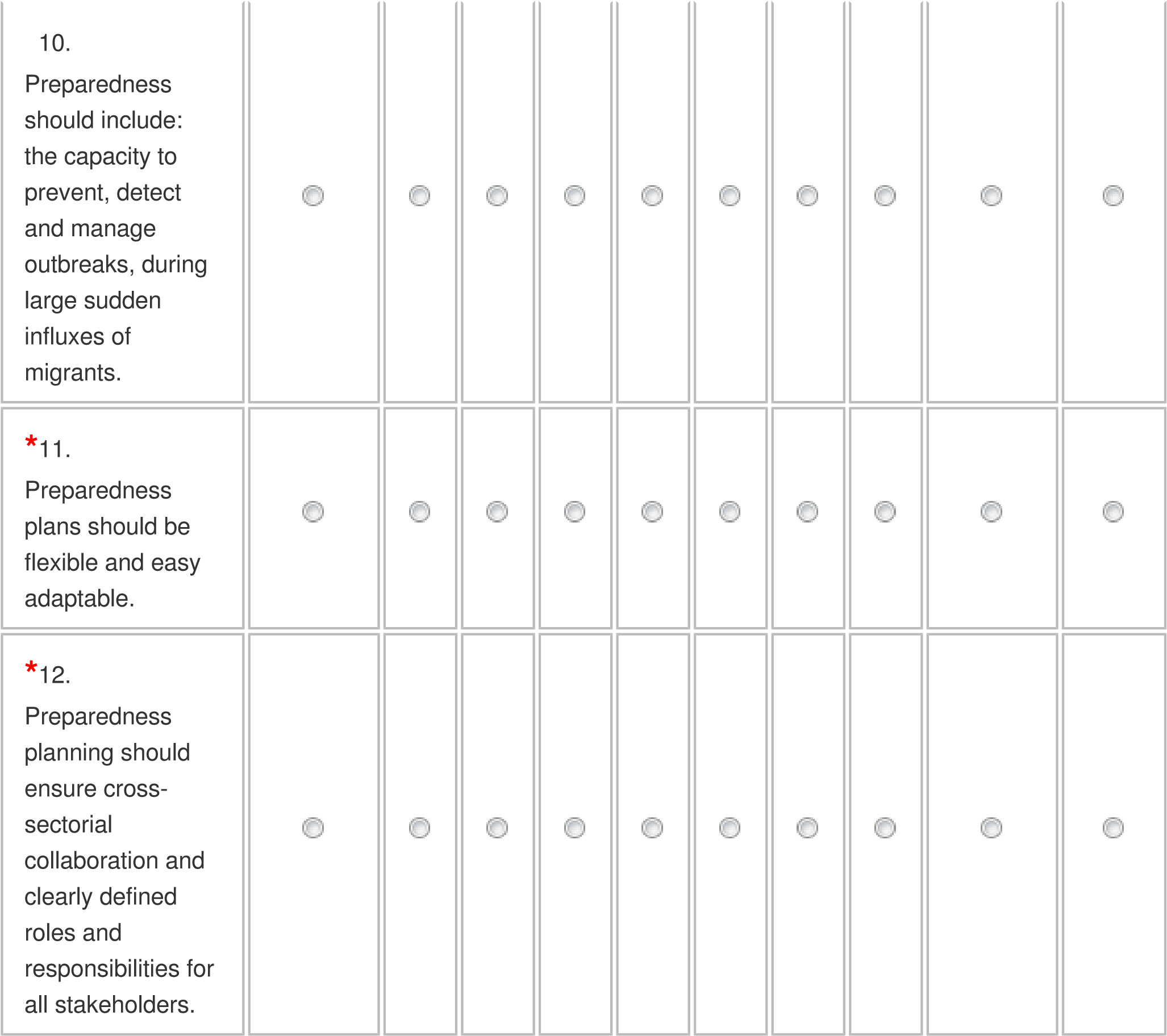


-

. Whole-of

13

government (i.e.

formal and

informal

networks)

biosafety and

biosecurity

system should be

in place for

human, animal,

and agriculture

facilities.


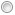

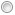

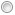

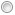

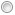

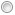

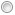

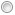

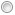

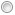


-

14

. Multi

sectorial and

multi-stakeholder

coordination,

command and

control should be

based on

established

infrastructure and

should be

continually

strengthened

during the

planning process.


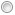

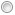

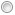

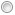

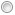

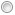

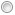

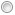

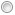

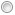


*****


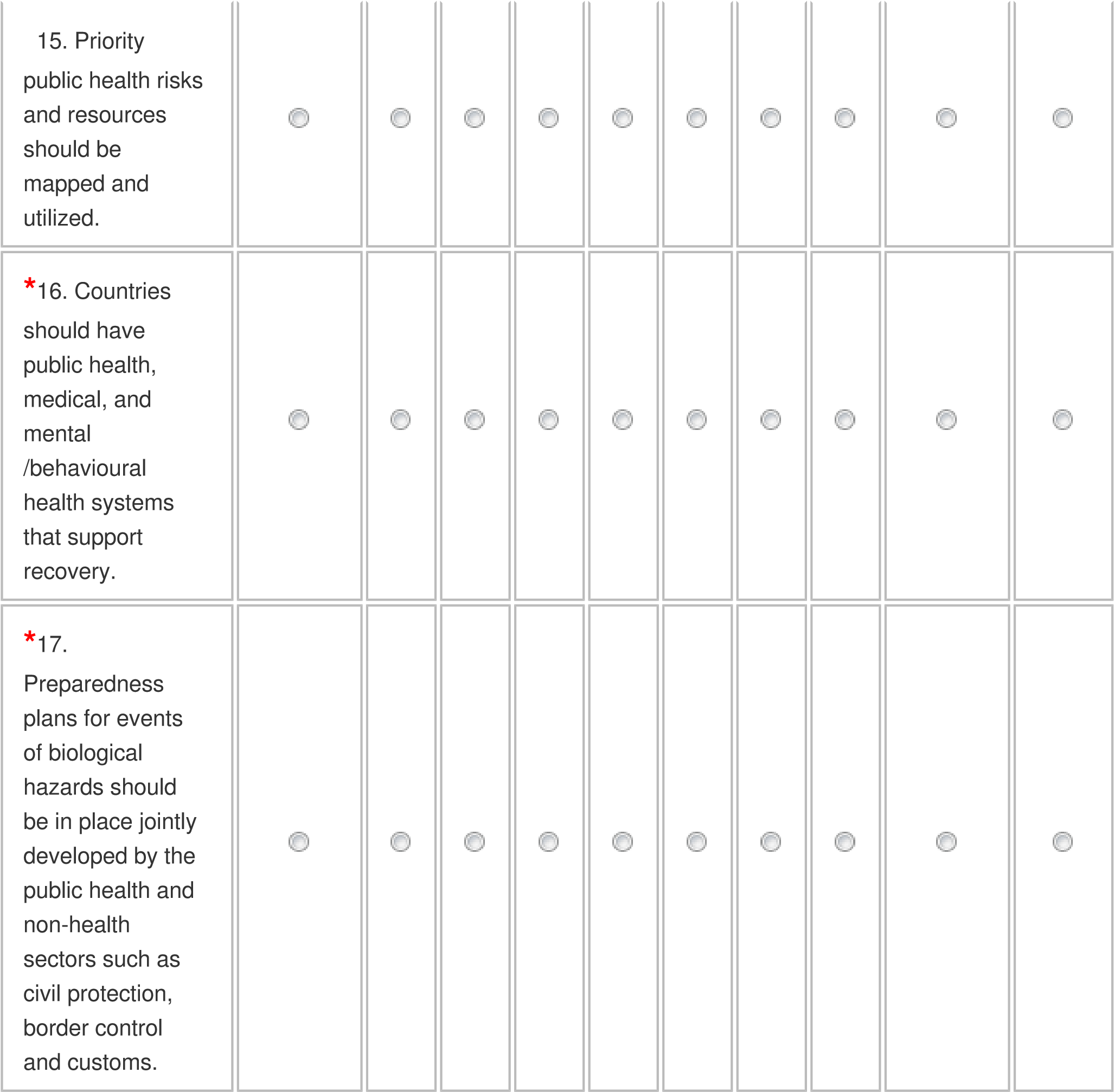


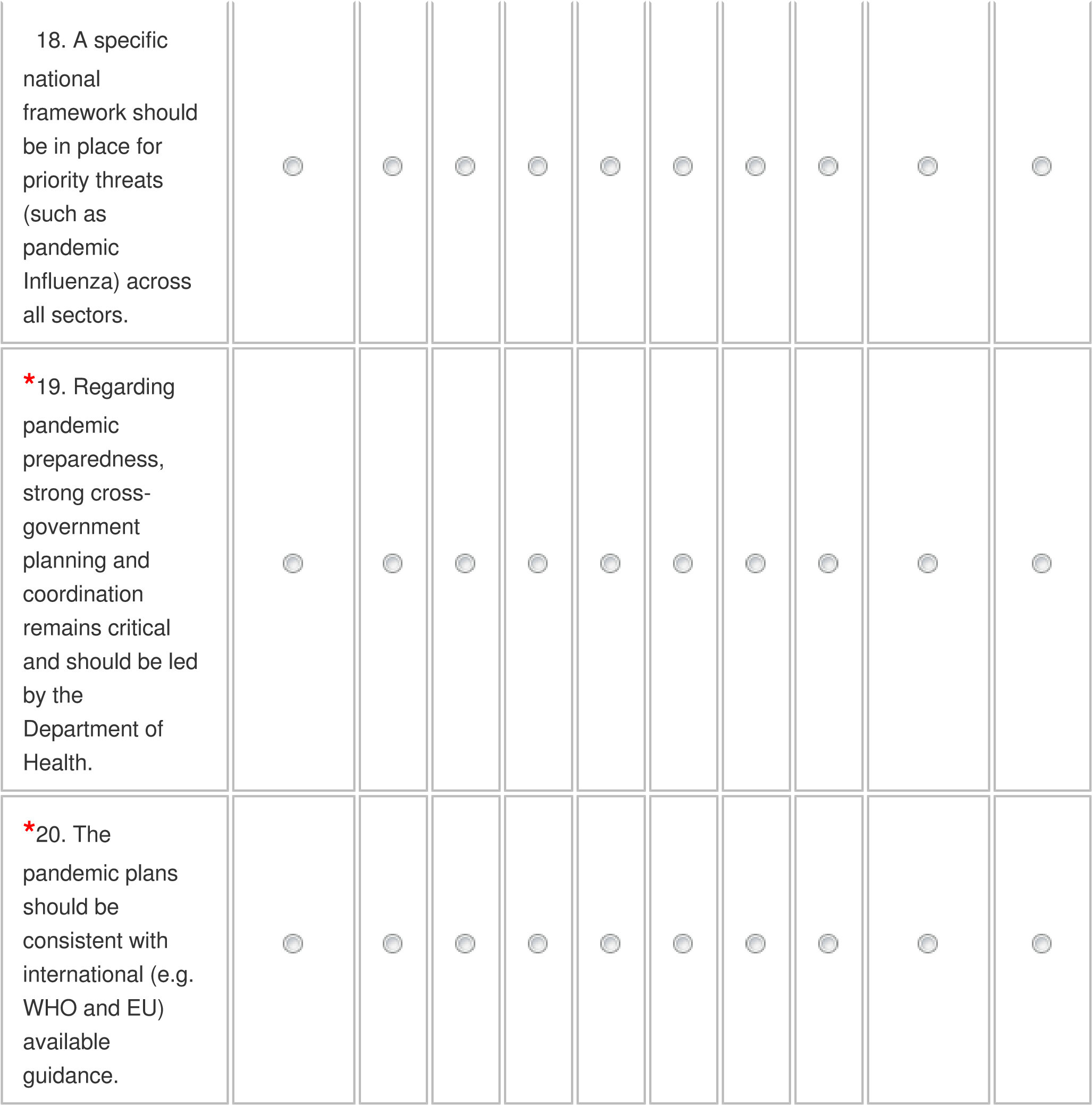


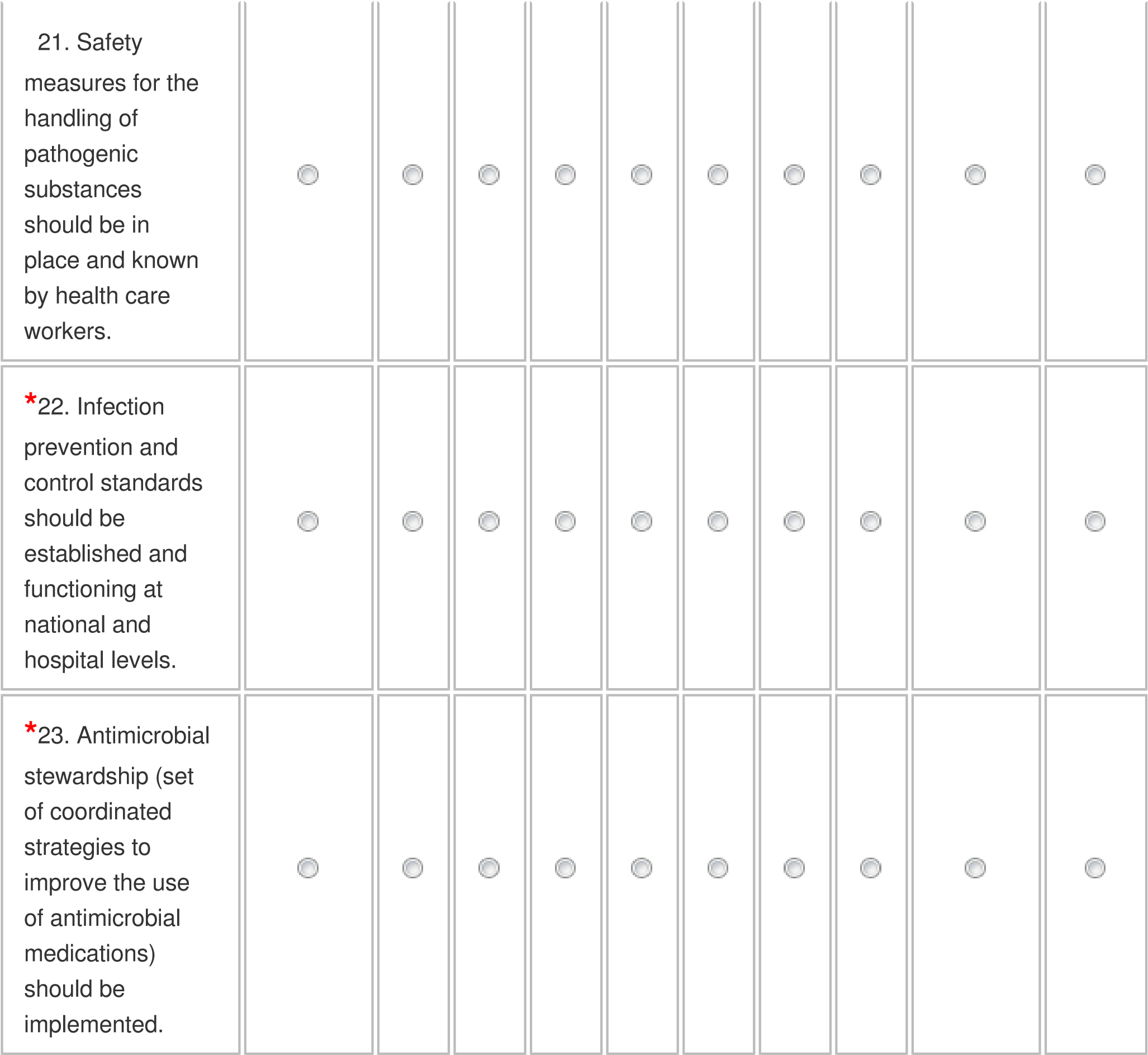


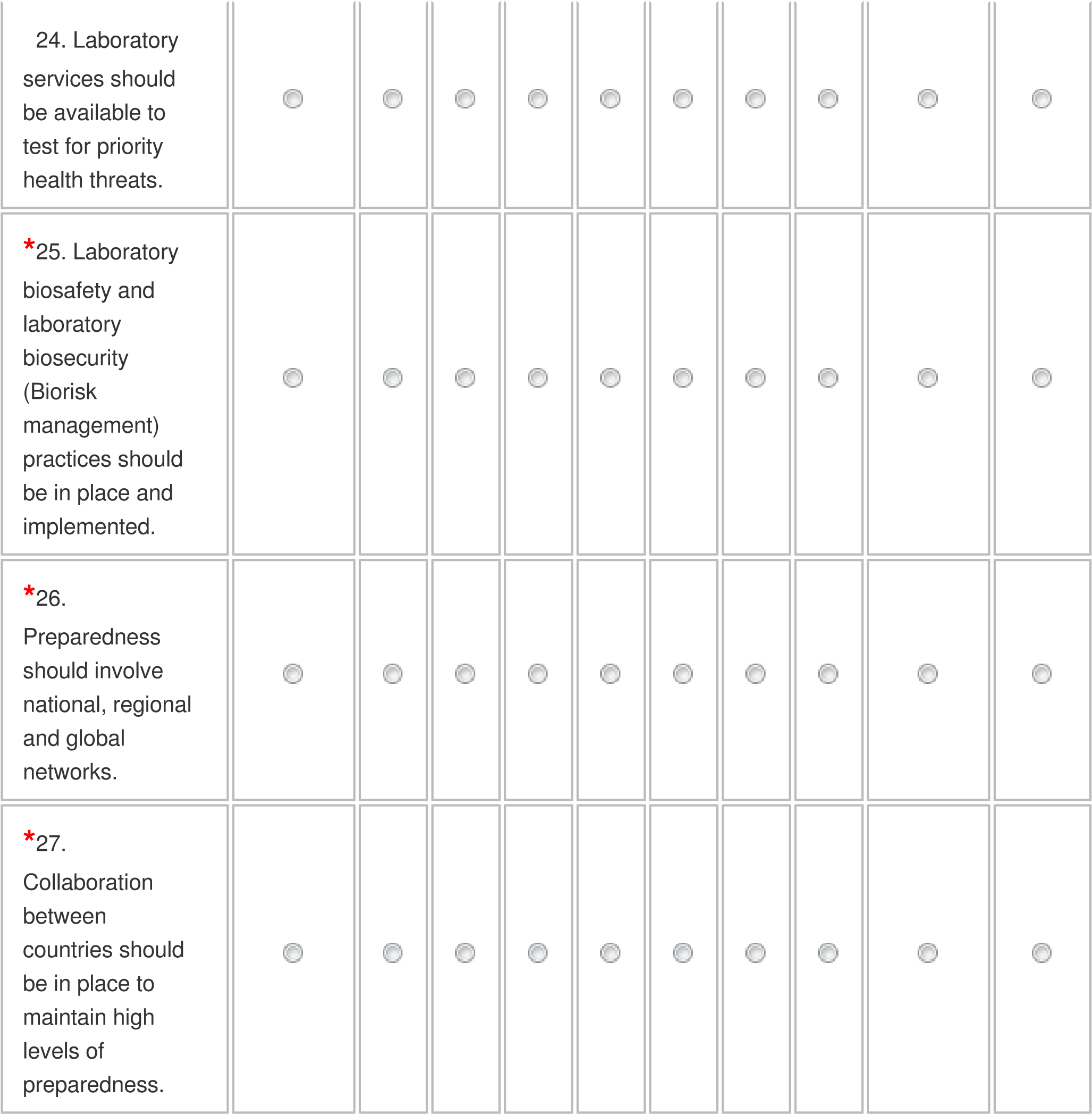


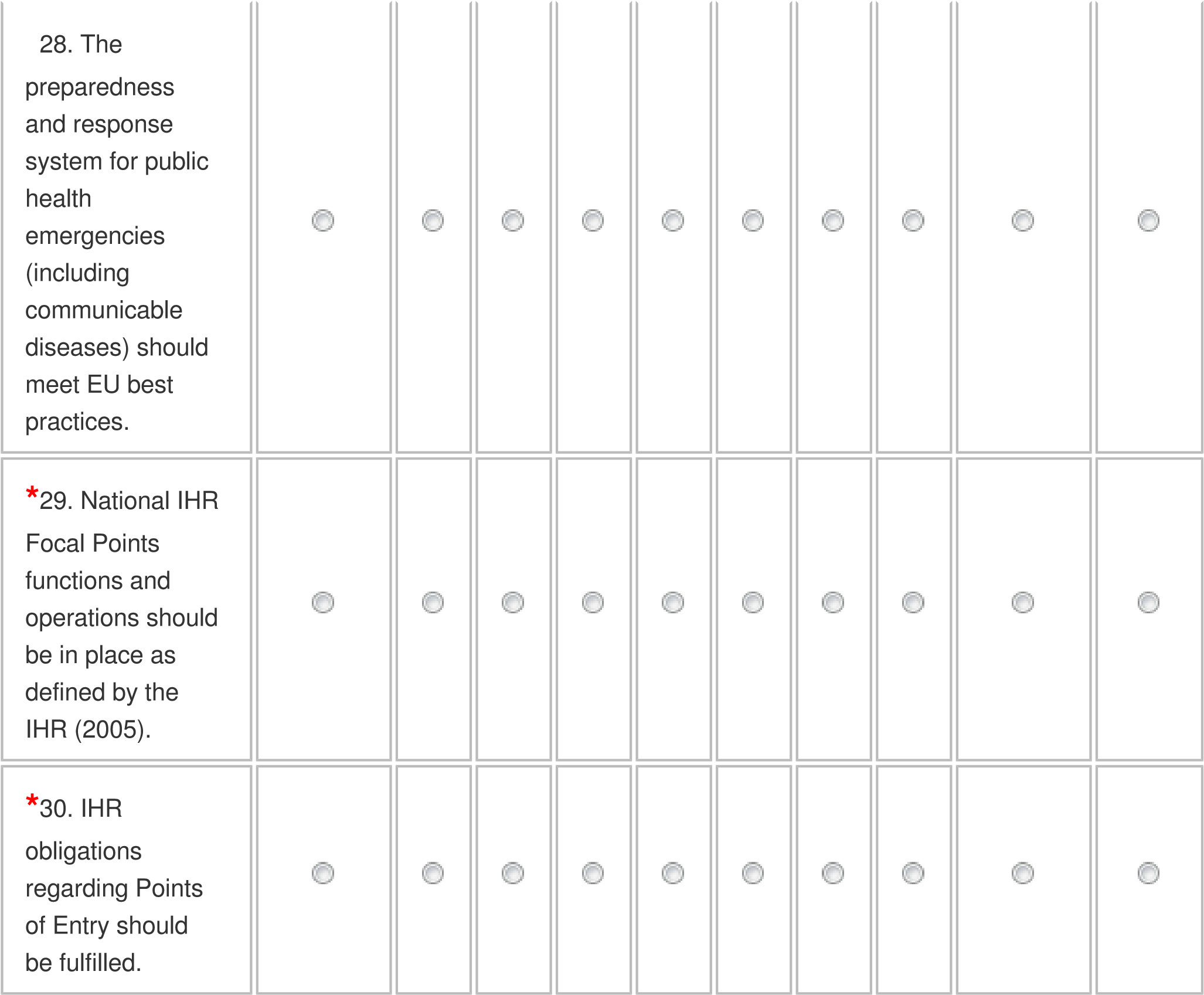


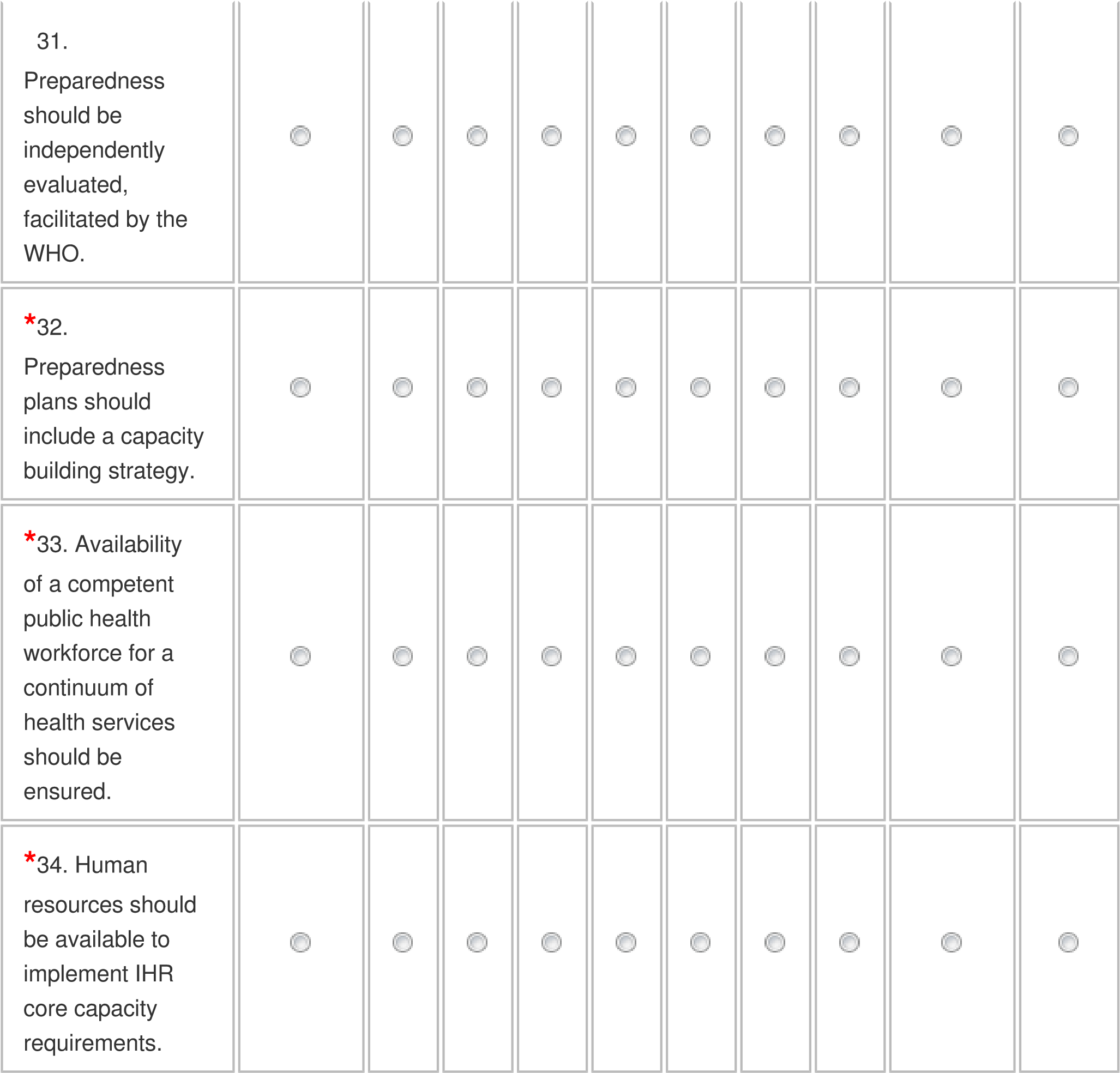


35

. A system

should be in

place for medical

evacuation of

health personnel

abroad during a

public health

emergency.


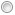

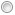

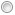

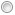

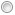

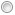

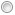

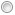

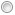

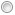


## 2.2 Emergency Risk Communication

Please appraise the relevance of the following statements as concerns strategic planning for public health emergency preparedness.

Not

1

relevant

2

3

4

5

6

7

8

9

Very

relevant

I

don't

know

. Public

36

Health

authorities (i.e.

decision-

makers)should

establish

communication

policies and

procedures to

develop,

coordinate, and

disseminate

information

related to an

event of public

health concern.


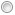

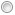

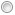

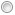

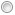

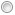

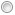

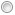

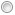

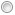


*****


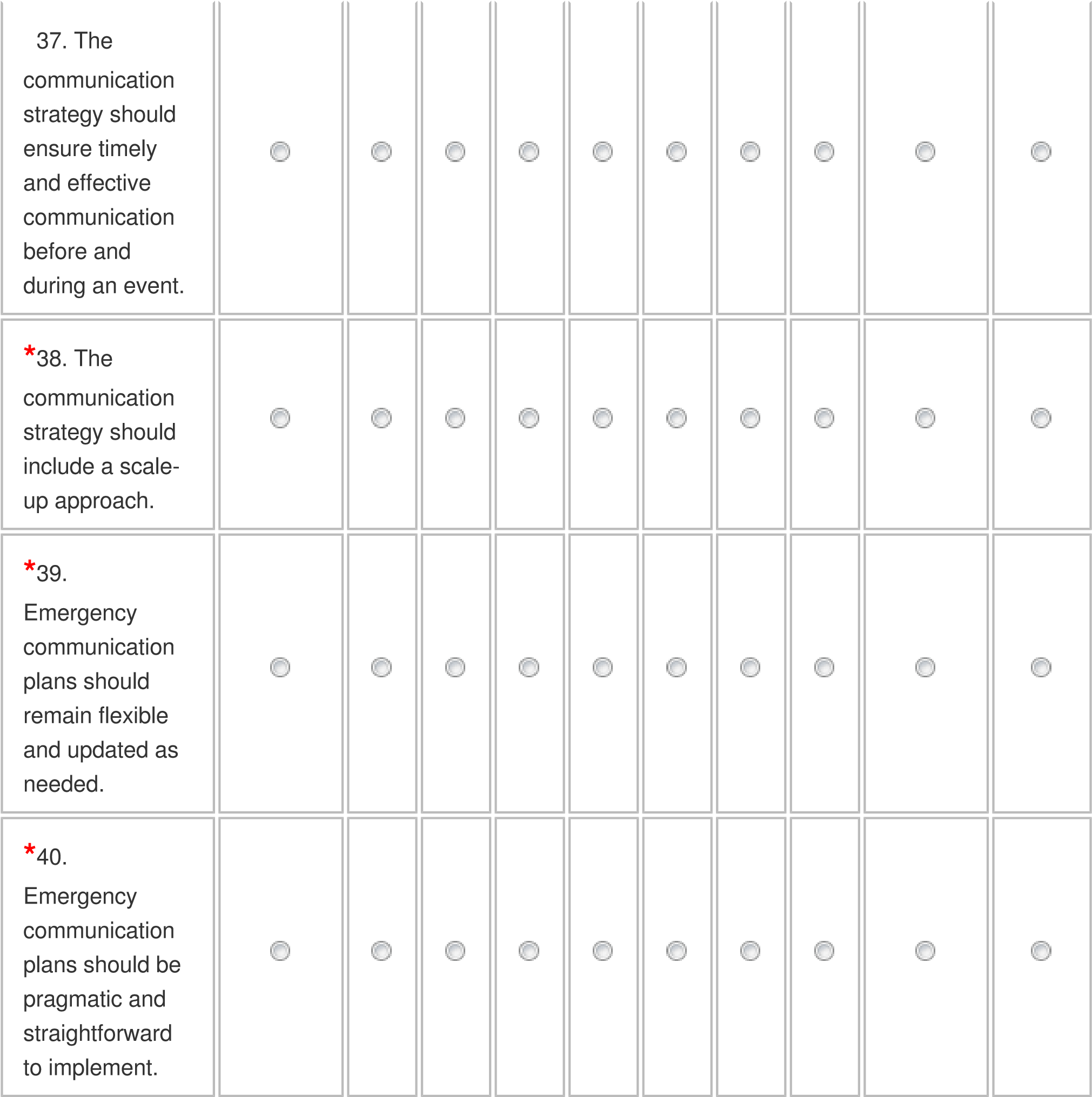


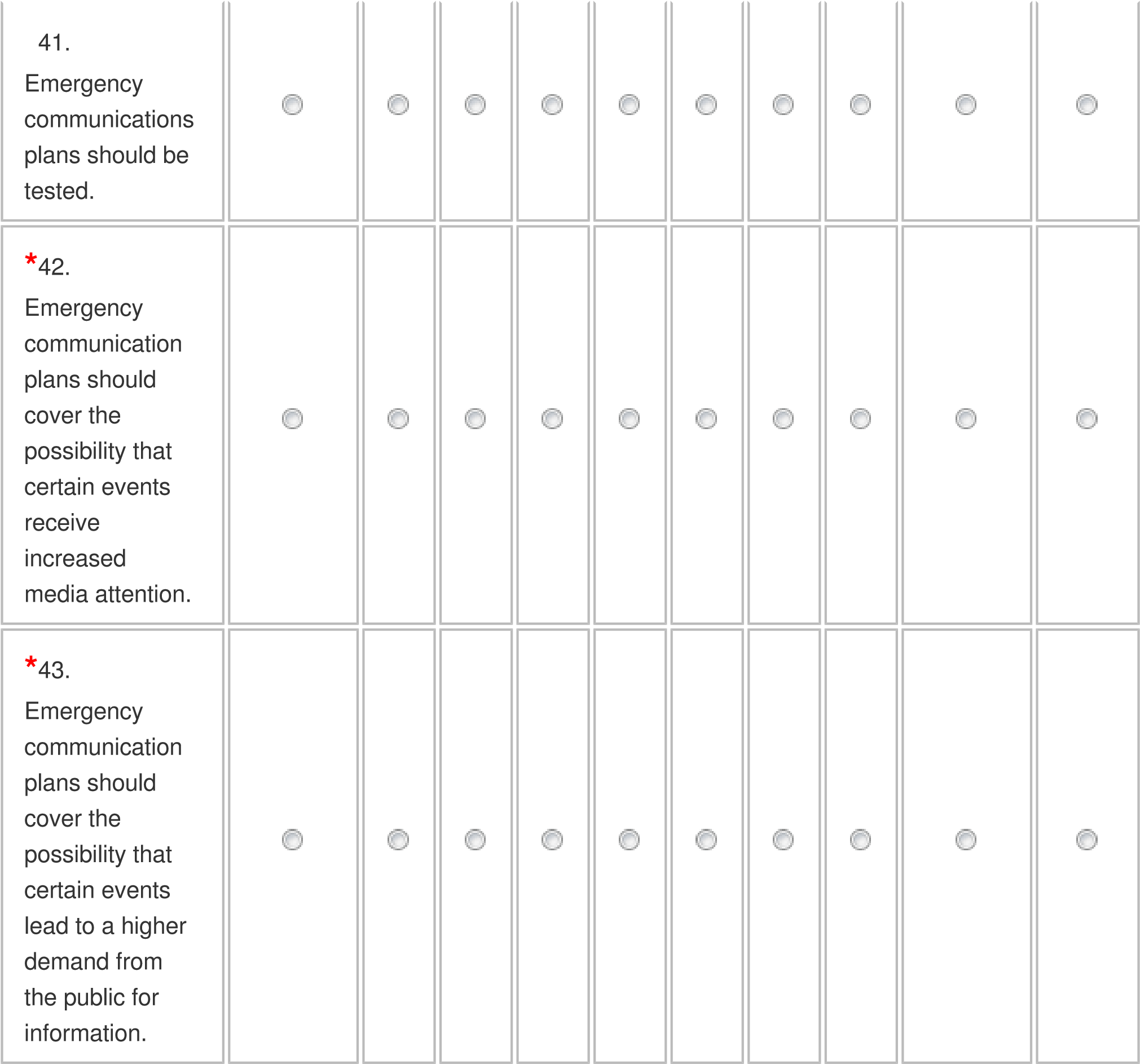


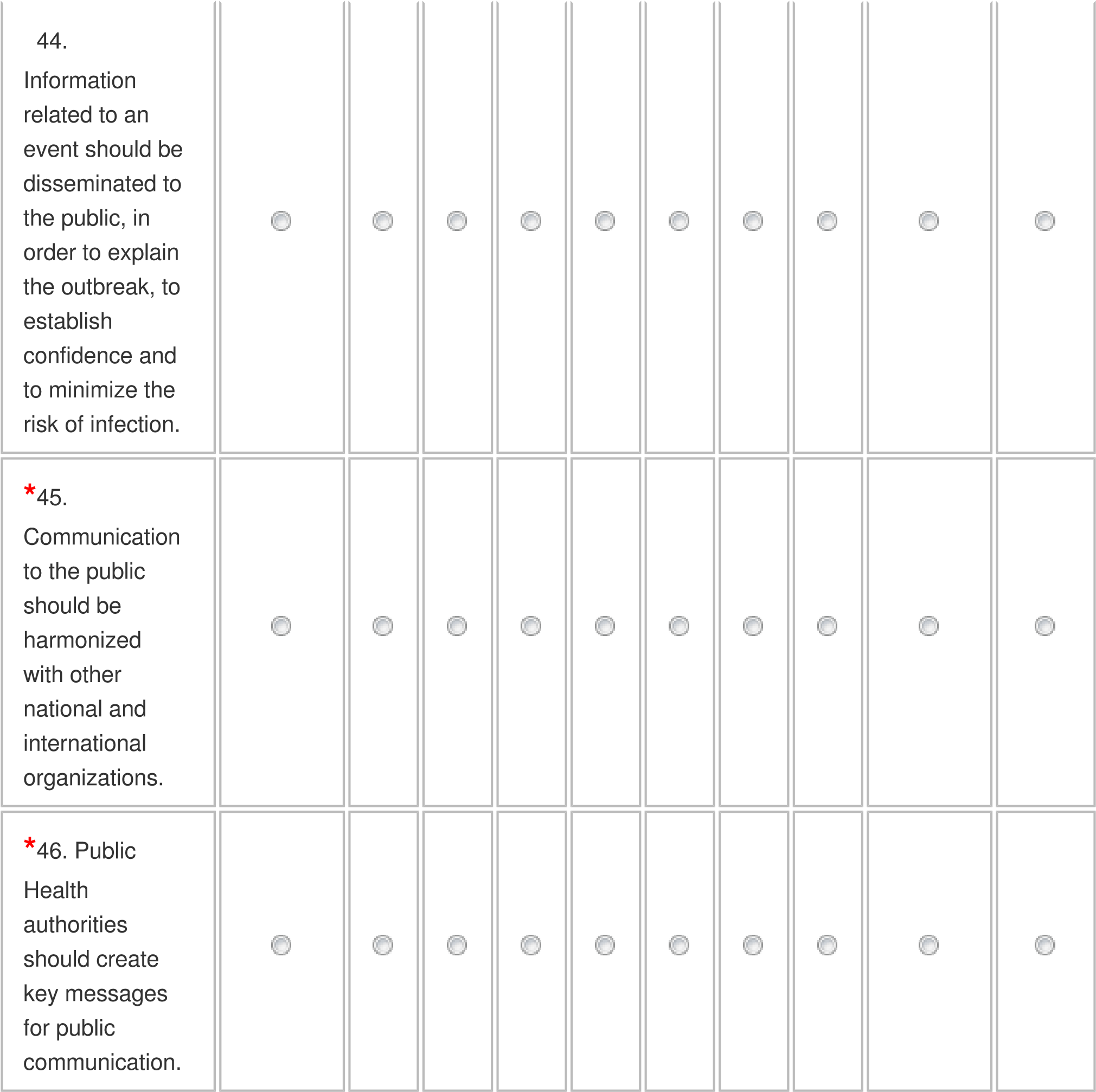


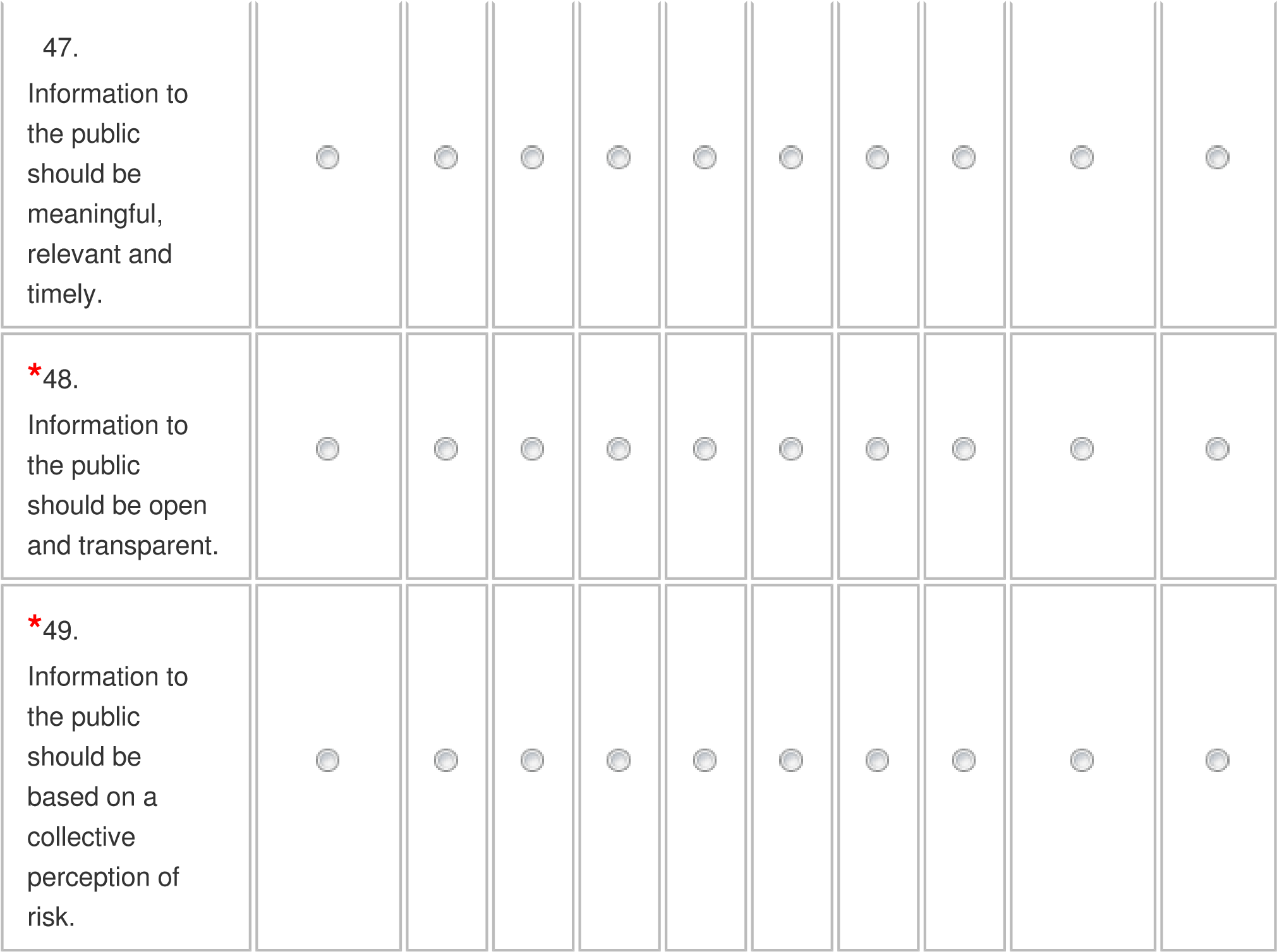


50

.

Communication

to the public

should take into

account

characteristics

of the

population such

as language,

social, religious,

cultural, political

and/or

economic

aspects.


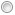

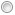

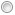

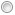

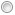

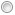

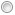


51

. Public

Health

authorities

should set up

multiple risk

communication

channels (e.g.

website, E-mail,

subject-specific

telephone lines).

*****

. Public

52

Health

authorities

should provide

timely

information and

guidance about

an event to

health and

other

professionals,

so they can

appropriately

respond to the

public.

53

. Public

Health

authorities

should prepare

*ad hoc*

information

material for

different

stakeholders (e.

g. simplified

case definitions

for community

use).

*****

. Public

54

Health

organizations (i.

e. scientific

advisors)

should address

ethical issues

and produce

plans for

vulnerable

populations.

55

. Public

Health

organizations

should counter

misinformation

and prevent

stigma, even

among

educated

hospital staff.

*****

If you have any questions remarks about the statements above, please use the box below. If not, just write "No remarks".

If you would like to add any expertise or statements (e.g. statements that are in your countries preparedness plan but not in this questionnaire) please use the box below. If not, just write "No additional statements".

# 3 - Capacity building & maintenance (Education, training & simulation exercise)

Please appraise the relevance of the following statements as concerns strategic planning for public health emergency preparedness.

If you have any questions remarks about the statements above, please use the box below. If not, just write "No remarks".

If you would like to add any expertise or statements (e.g. statements that are in your countries preparedness plan but not in this questionnaire) please use the box below. If not, just write "No additional statements".

11

. Public

Health

authorities

should

conduct

exercises to

test the actual

functionality

of IHR

capacity.

1

Not

relevant

2

3

4

5

6

7

8

9

Very

relevant

I

don't

know

**4 - Surveillance**

Please appraise the relevance of the following statements as concerns strategic planning for public health emergency preparedness.

If you have any questions remarks about the statements above, please use the box below. If not, just write "No remarks".

If you would like to add any expertise or statements (e.g. statements that are in your countries preparedness plan but not in this questionnaire) please use the box below. If not, just write "No additional statements".

19

. Public

Health

authorities

should have

reporting

networks and

protocols in

place

**5**

**- Risk assessment**

Please appraise the relevance of the following recommendations to qualify as generic elements of the guidance on strategic planning for public health emergency preparedness

1

Not

relevant

2

3

4

5

6

7

8

9

Very

relevant

I

don't

know

17

. Based on

the disease

characteristics,

the risk

assessment

team should

decide how

frequently the

risk

assessment

should be

updated.

If you have any questions remarks about the statements above, please use the box below. If not, just write "No remarks".

If you would like to add any expertise or statements (e.g. statements that are in your countries preparedness plan but not in this questionnaire) please use the box below. If not, just write "No additional statements".

**6**

**- Risk and crisis management**

## 6.1 General aspects

Please appraise the relevance of the following statements to qualify as generic elements of the guidance on strategic planning for public health emergency preparedness

Please appraise the relevance of the following statement as concerns strategic planning for public health emergency preparedness.

1

Not

relevant

2

3

4

5

6

7

8

9

Very

relevant

I

don't

know

Please appraise the relevance of the following recommendations to qualify as generic elements of the guidance on strategic planning for public health emergency preparedness

1

Not

relevant

2

3

4

5

6

7

8

9

Very

relevant

I

don't

know

If you have any questions remarks about the statements above, please use the box below. If not, just write "No remarks".

If you would like to add any expertise or statements (e.g. statements that are in your countries preparedness plan but not in this questionnaire) please use the box below. If not, just write "No additional statements".

**7**

**- Post-event evaluation**

Please appraise the relevance of the following statements as concerns strategic planning for public health emergency preparedness.

Not

1

relevant

2

3

4

5

6

7

8

Very

9

relevant

I

don't

know

1

. Public

Health

authorities

should

assess the

level of

preparedness

by evaluating

events of

public health

concern.

*****

7

. Post

-

event

evaluations

should

consist of an

external peer

review,

inviting

another IHR

State Party

and the WHO

secretariat to

participate

and EU

relevant

agencies.

8

. Lessons

learned from

all relevant

sectors

should be

systematically

recorded in a

post-event

report.

*****

*****

If you have any questions remarks about the statements above, please use the box below. If not, just write "No remarks".

If you would like to add any expertise or statements (e.g. statements that are in your countries preparedness plan but not in this questionnaire) please use the box below. If not, just write "No additional statements".

# 8 - Implementation of lessons learned

Please appraise the relevance of the following statements as concerns strategic planning for public health emergency preparedness.

1

Not

relevant

2

3

4

5

6

7

8

9

Very

relevant

I

don't

know

.

1

Experiences

and lessons

learned,

coming forth

from post-

event

evaluation or

exercises,

should be

reviewed

across all

relevant

sectors.

*****

If you have any questions remarks about the statements above, please use the box below. If not, just write "No remarks".

If you would like to add any expertise or statements (e.g. statements that are in your countries preparedness plan but not in this questionnaire) please use the box below. If not, just write "No additional statements".

Thank you very much for completing the survey!
