# Supplementary material for: Towards defining core principles of public health emergency preparedness: scoping review and Delphi consultation among European Union country experts
Source: BMC Public Health. 2020 Oct 1;20:1482. doi: 10.1186/s12889-020-09307-y (PMC7527265; doi:10.1186/s12889-020-09307-y)
Supplement: Supplementary file 2 — Additional file 2. Questionnaire baseline set. This file contains a PDF file of the questionnaire as it was send to the participating experts. [file 12889_2020_9307_MOESM2_ESM.docx]

Contribution ID: 34edfeaa-cbdf-48d1-8130-ee33d6f41208 Date: 09/05/2017 21:12:36

Health Emergency Preparedness Self-Assessment

(HEPSA) tool - Selection of a Baseline Set of Indicators

Fields marked with * are mandatory.

**Baseline Set of Indicators for the HEPSA-tool**

Thank you very much for taking the time to fill out this questionnaire. This will take between twenty to thirty minutes.

**Background**

The European Centre for Disease Prevention and Control (ECDC) with the collaboration of the Dutch

National Coordination Centre for Communicable Disease Control (LCI) are developing a Health Emergency Preparedness Self Assessment tool (HEPSA-tool). With this tool, EU/EEA countries have the ability to self-assess their own level of preparedness, and to identify potential gaps and vulnerabilities.

The HEPSA-tool will consist of two sets of indicators: a Baseline Set of Indicators and a Comprehensive Set of Indicators. The Baseline Set of Indicators represents the minimum set of indicators that are needed to achieve preparedness, applicable for all countries, irrespective of the organization of their health system. The Comprehensive Set of Indicators represents optimal preparedness, and is likely to require more time and resources.

The Comprehensive Set of Indicators was subject of an earlier questionnaire and serves as input for the *Handbook on Strategic Planning for Public Health Emergency Preparedness*. Therefore, if you filled in the questionnaire for the Handbook, the items of the current questionnaire may be familiar to you. The Baseline Set of Indicators were preselected from the Comprehensive Set of Indicators.


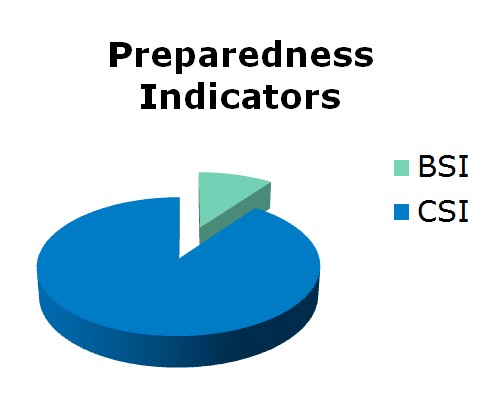


The Baseline Set of Indicators was selected based on the following requirements:

- The indicator is **essential** for preparedness;
- The indicator is a **core function** of preparedness;
- The indicator is **feasible** for all countries;
- The indicator functions as an **enabler** for other indicators.

This questionnaire is designed to obtain feedback from EU/EEA countries, in order to assess whether the selected Baseline Set of Indicators is indeed perceived to represent this minimum set of indicators. To accomplish this, we request that a National Focal Point for Preparedness and Response member/alternate or another expert with at least three years of experience in preparedness planning fills in this questionnaire.

**Instructions**

The questionnaire consists of seven domains (demonstrated on the next page). Per domain, the Baseline Set of Indicators is highlighted in yellow. Along with the presented additional indicators they form the Comprehensive Set of Indicators as selected in the previous survey related to the development of the Handbook on Strategic Planning (subject of the *Handbook on Strategic Planning*

*for Public Health Emergency Preparedness*, under development).

We ask you to indicate, per domain, if you feel the selected indicators are indeed representing the minimum requirements for preparedness. Per recommendation, you are asked to score the appropriateness of that recommendation as as minimum requirement for the domain, on a scale from 1 to 9. We kindly ask you to bear in mind at all times that for the self-assessment tool, we are looking for a **minimum set of indicators that are needed to achieve preparedness, applicable for all countries, irrespective of the organization of their health system.** At the end of each section, there is also the opportunity to provide open-text comments.

It is possible to save your response to the questionnaire and restart at a later moment.

We thank you very much for your time!

# Overwiew of domains

The table below demonstrates the current domains with the number of recommendations of the CSI and the number of the proposed recommendations for the BSI. This questionnaire will address every domain systematically.


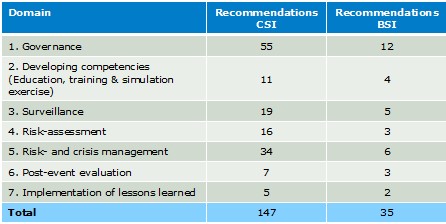


Please find below an overview of pre-selected recommendations for the Baseline Set of Indicators, which will be demonstrated throughout this questionnaire as well.

Handbook_on_Strategic_planning_for_Public_Health_Emergency_Preparedness.pdf

# 1 - General information

- **Which country do you represent?**
- **For which organisation do you work?**

***What is your position?**

NFP


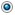

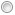

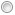


National expert Other

- **How many years of experience do you have in preparedness planning?**

< 3


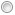

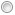

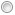

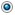


3 - 5

5 - 10

> 10

# 1 - Governance


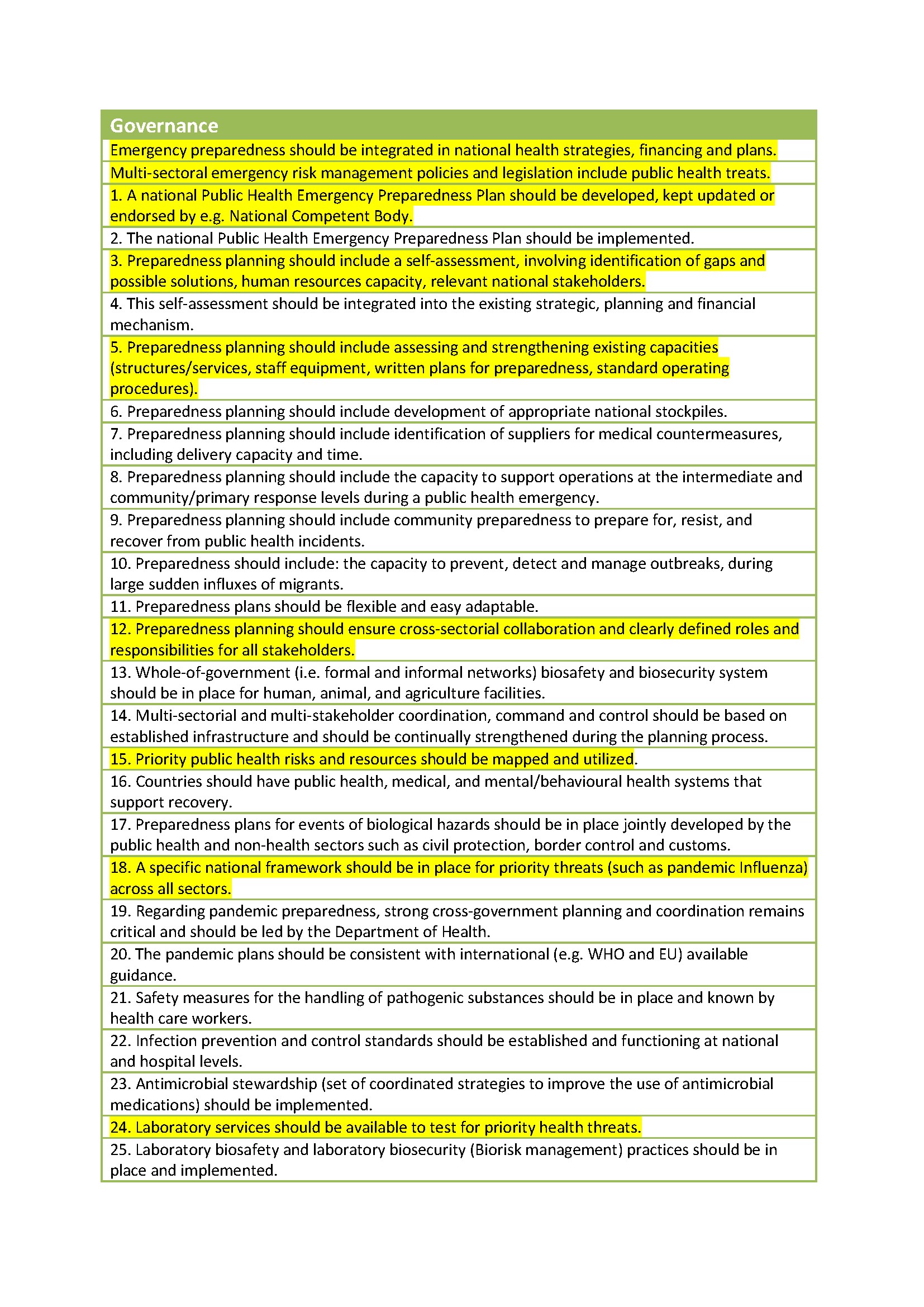


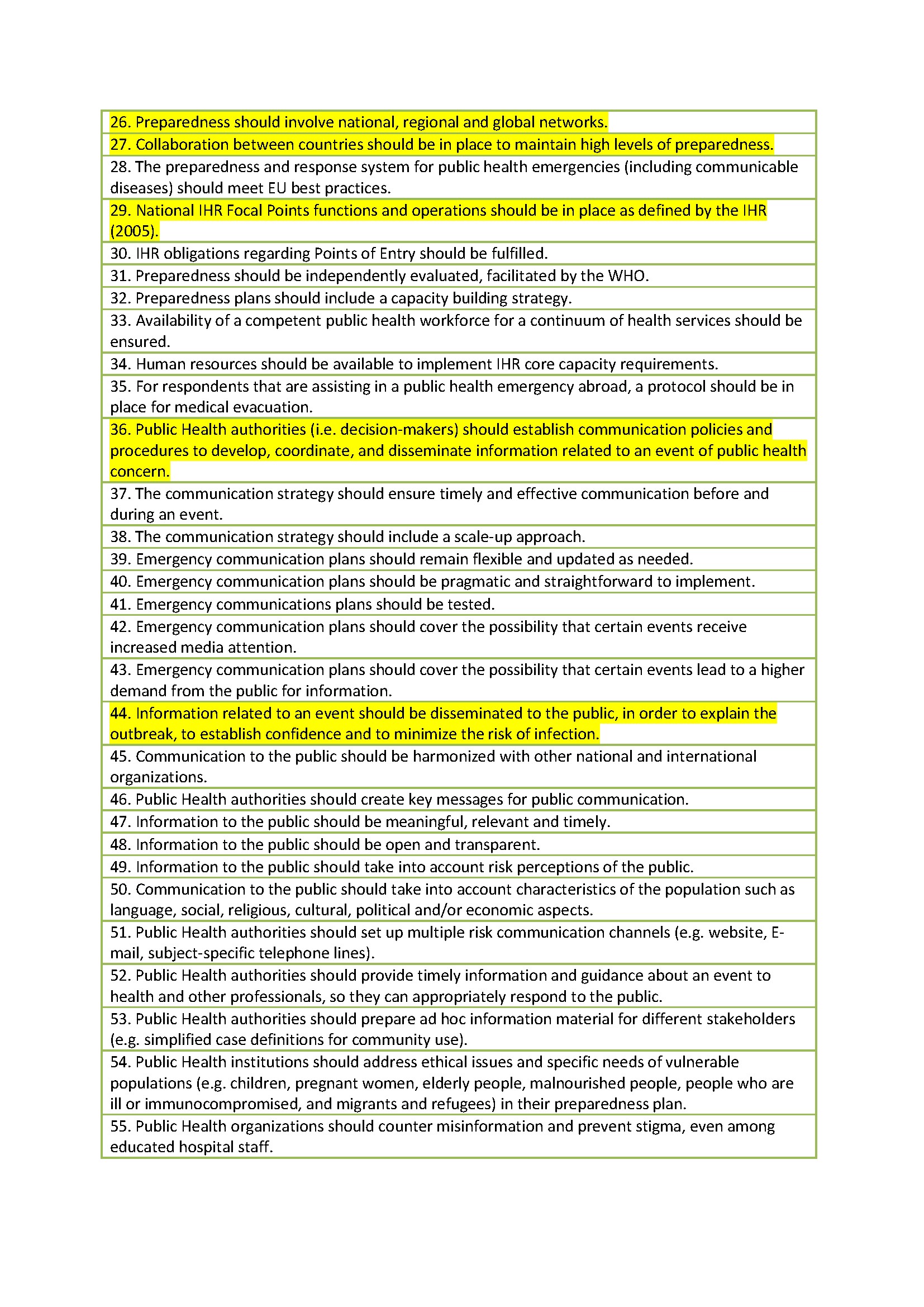


Please indicate below the appropriateness of inlcuding the selected indicators as a **baseline set of indicators, needed to achieve preparedness, applicable for all countries** (1 = not appropriate, 9 = very appropriate). **Score 1 indicates not appropriate and thus exclusion** of the indicator in the baseline set of indicators.


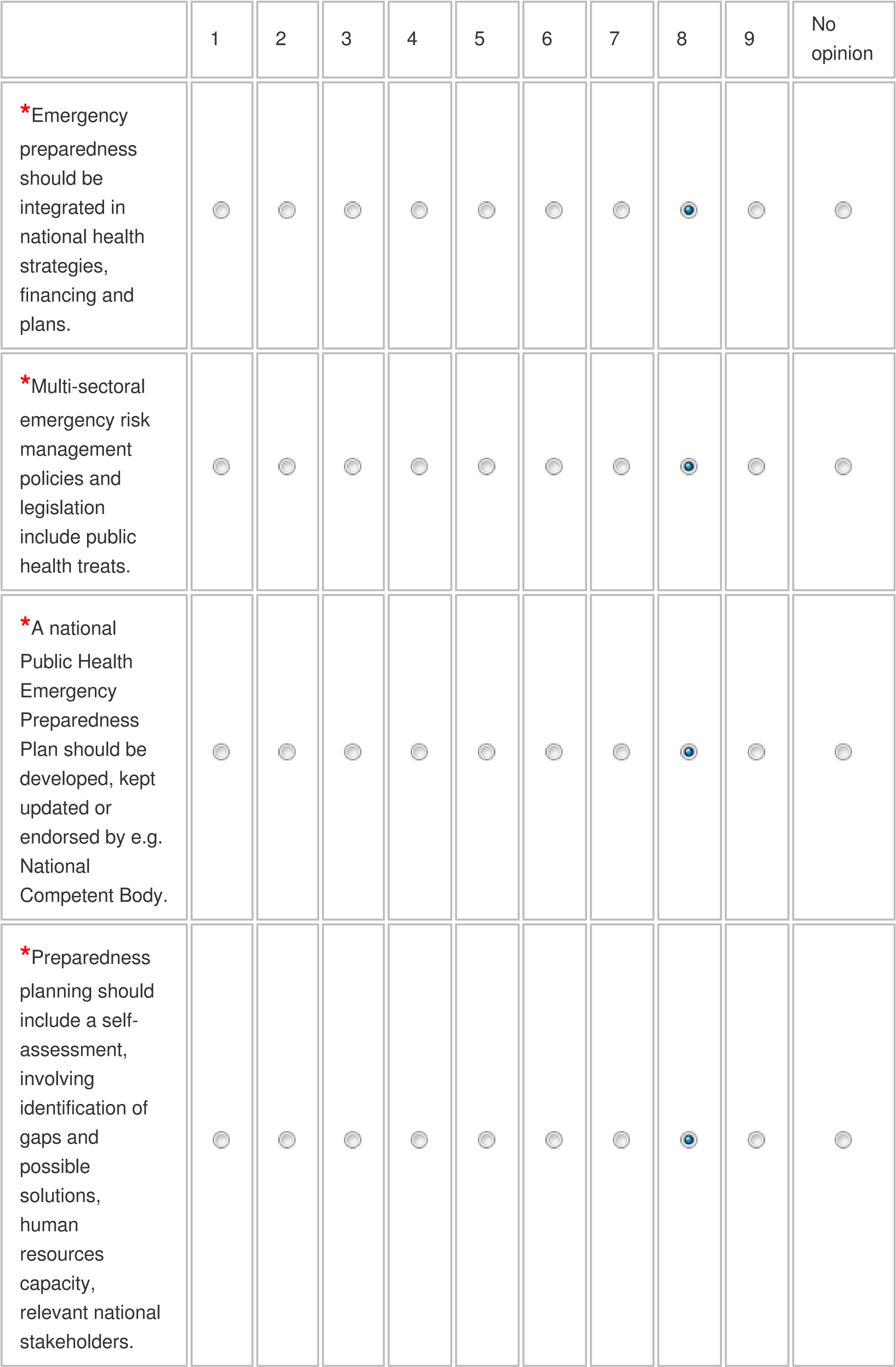


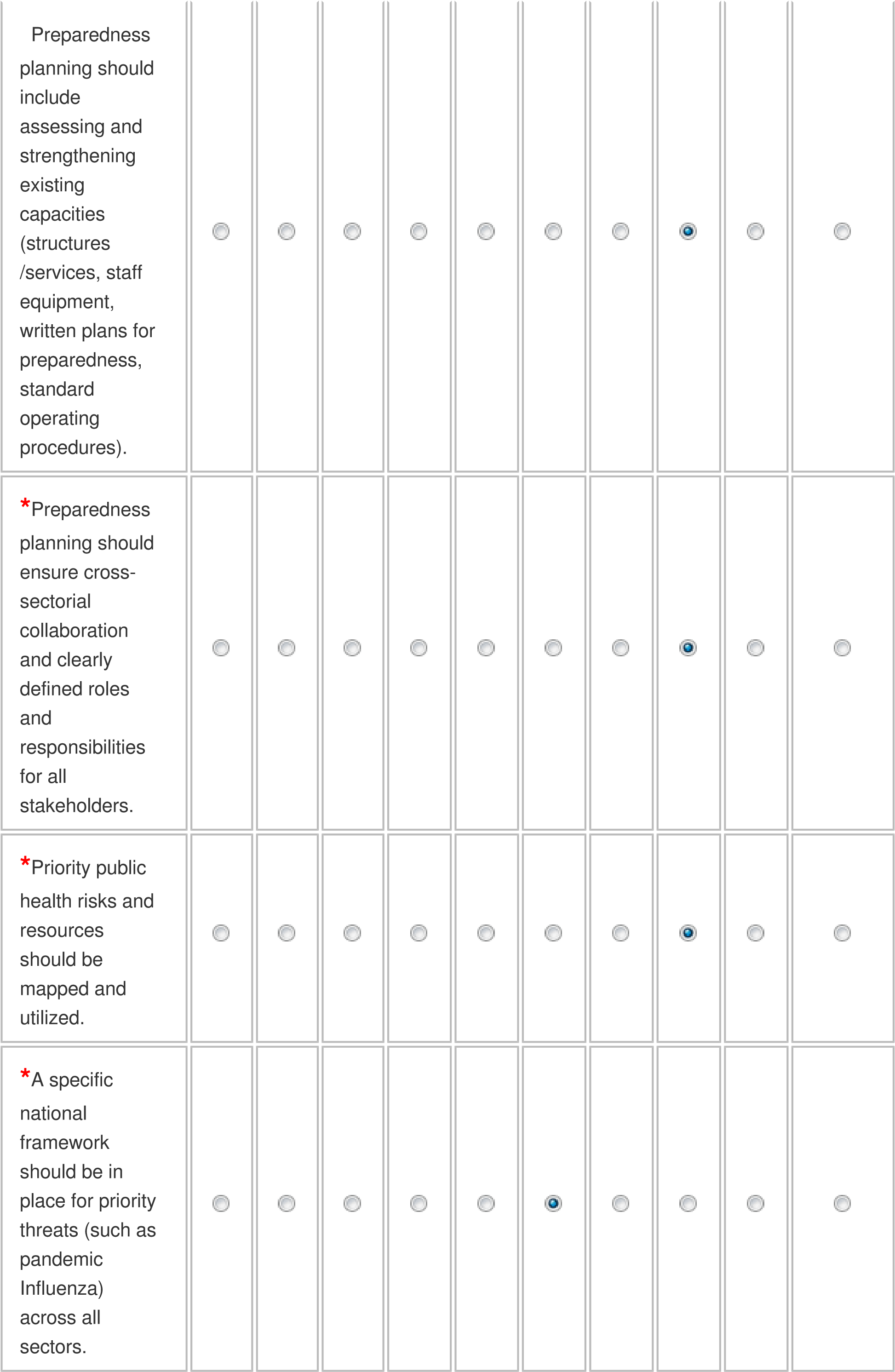


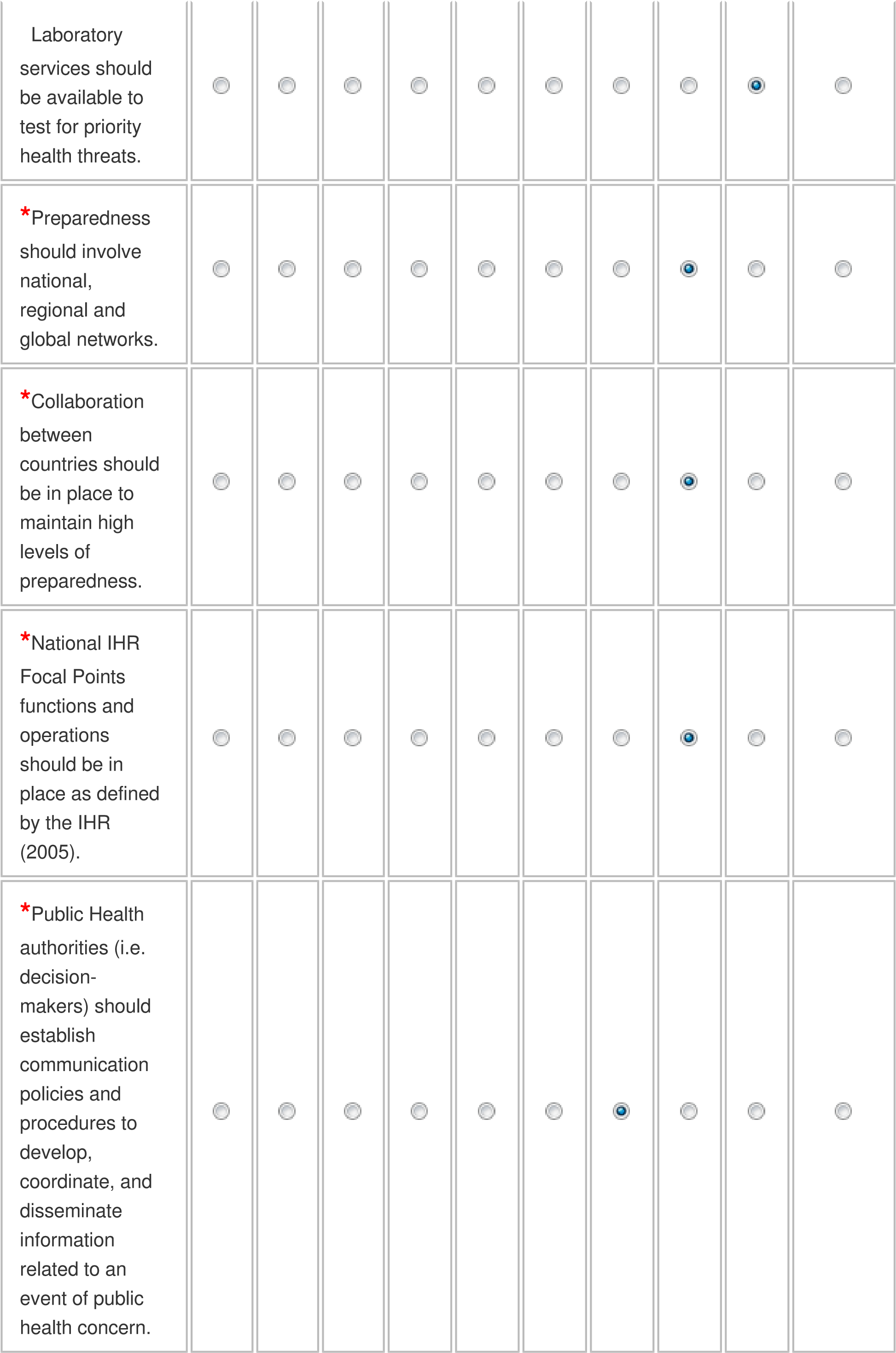


If you have any questions or remarks about the Baseline Set of Indicators in this domain, please write them down here:

If in your opinion other indicators of the complete list (as presented above) should be included in the Baseline Set of Indicators as well, please write the number of this indicator down here:

Information

related to an

event should be

disseminated to

the public, in

order to explain

the outbreak, to

establish

confidence and

to minimize the

risk of infection.


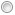

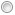

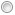

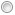

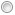

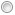

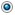

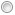

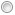

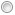


# 2 - Developing competencies (Education, training & simulation exercise)


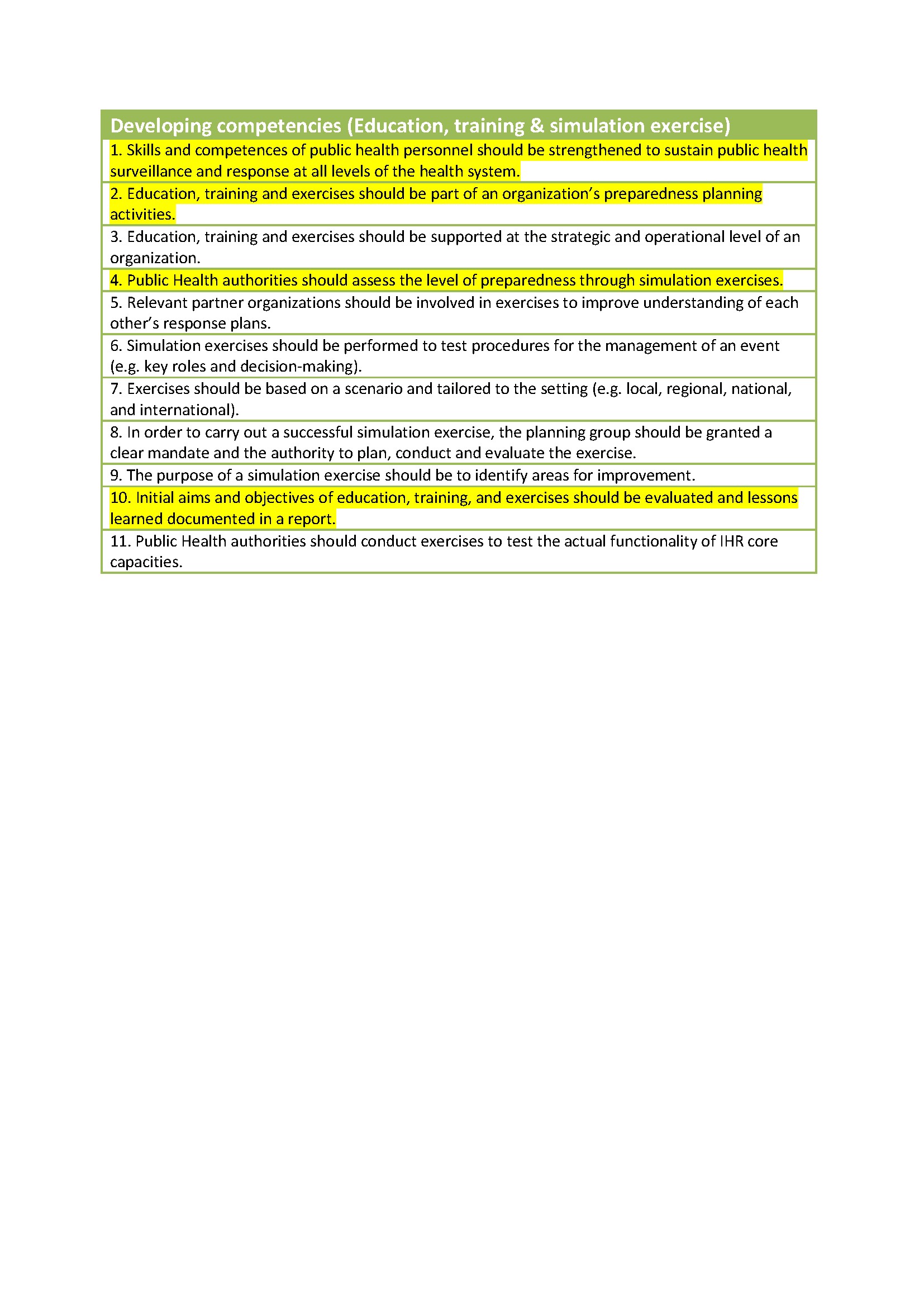


Please indicate below the appropriateness of inlcuding the selected indicators as a **baseline set of indicators, needed to achieve preparedness, applicable for all countries** (1 = not appropriate, 9 = very appropriate). **Score 1 indicates not appropriate and thus exclusion** of the indicator in the baseline set of indicators.


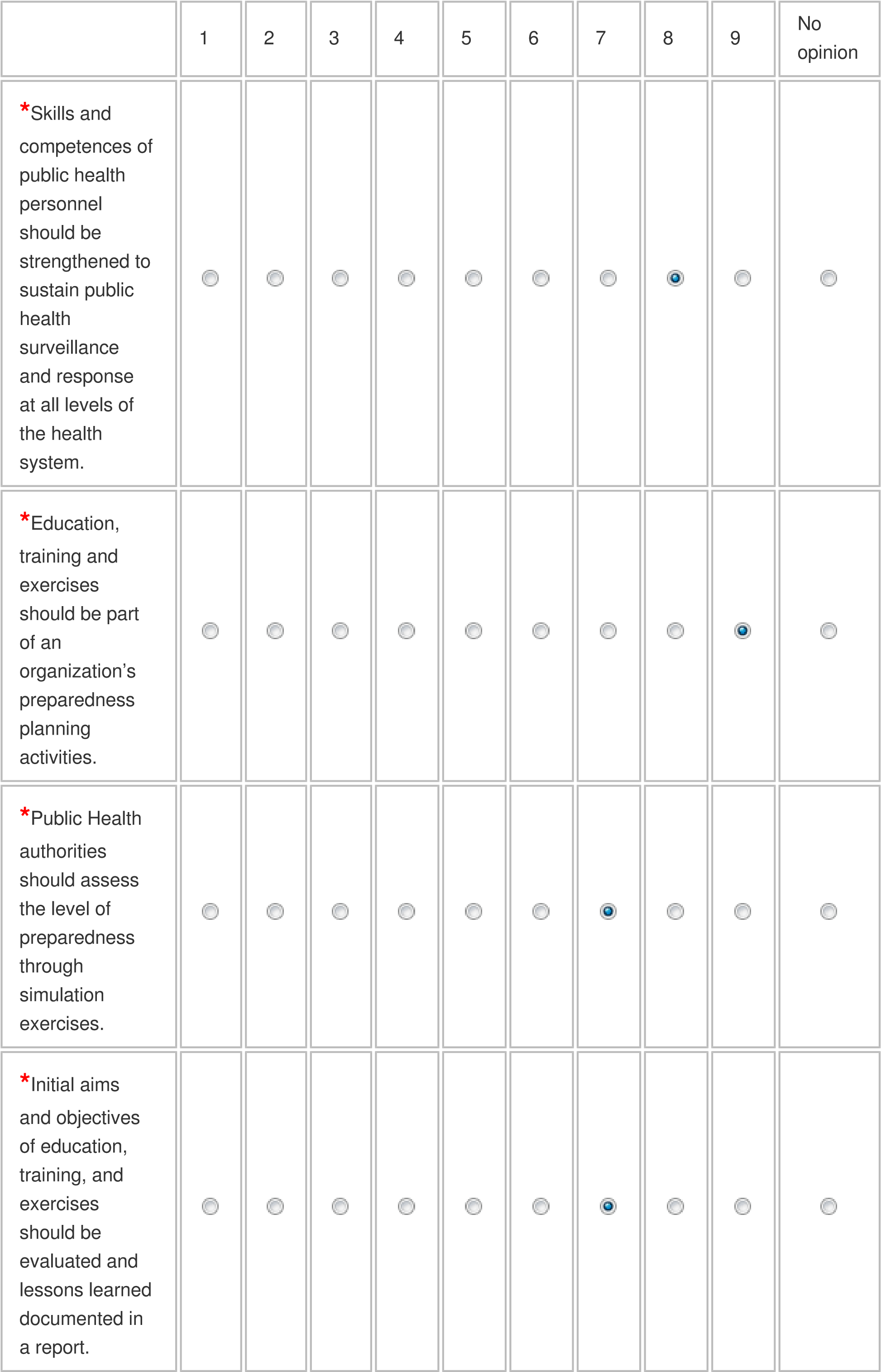


If you have any questions or remarks about the Baseline Set of Indicators in this domain, please write them down here:

If in your opinion other indicators of the complete list (as presented above) should be included in the Baseline Set of Indicators as well, please write the number of this indicator down here:

# 3 - Surveillance


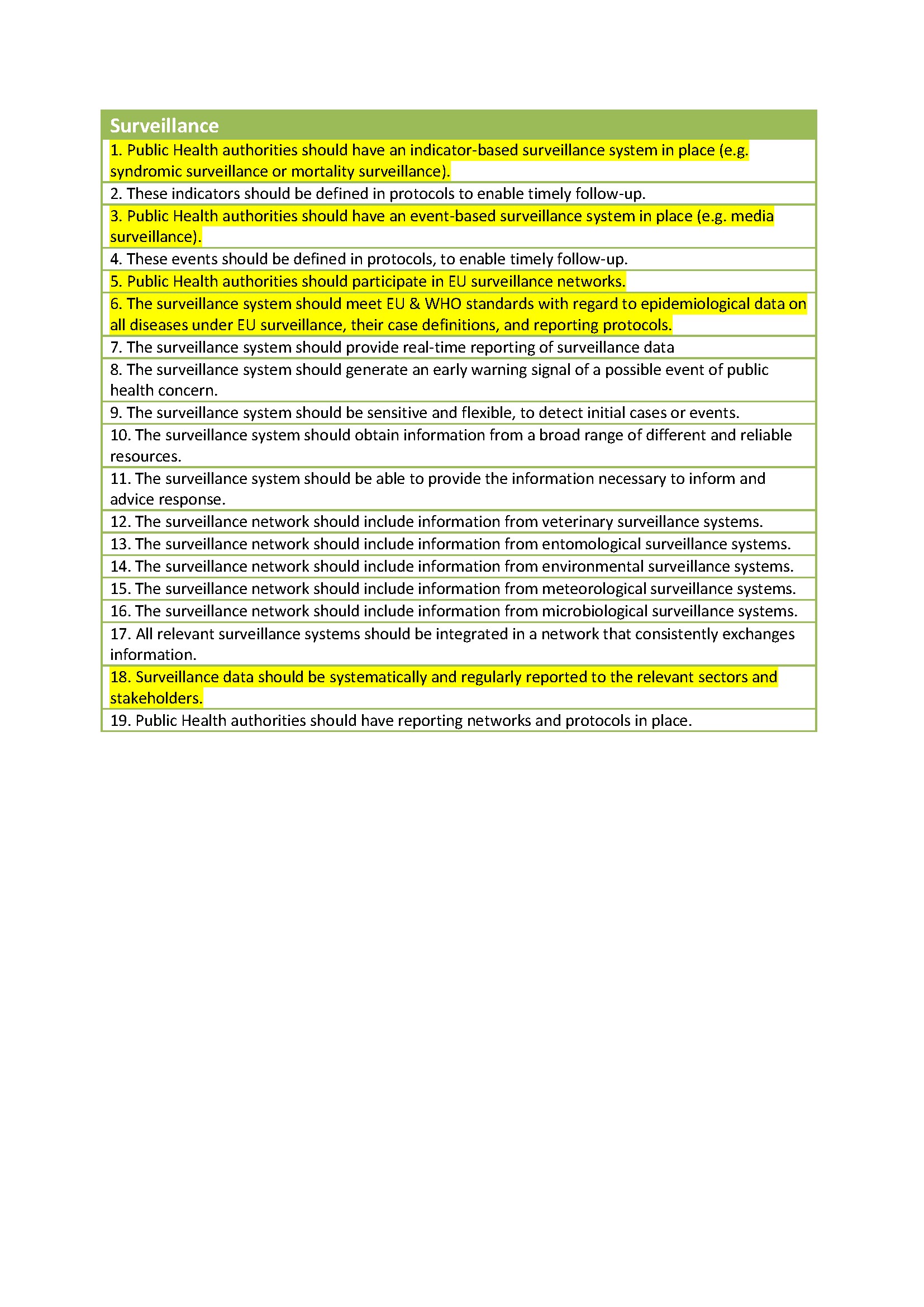


Please indicate below the appropriateness of inlcuding the selected indicators as a **baseline set of indicators, needed to achieve preparedness, applicable for all countries** (1 = not appropriate, 9 = very appropriate). **Score 1 indicates not appropriate and thus exclusion** of the indicator in the baseline set of indicators.


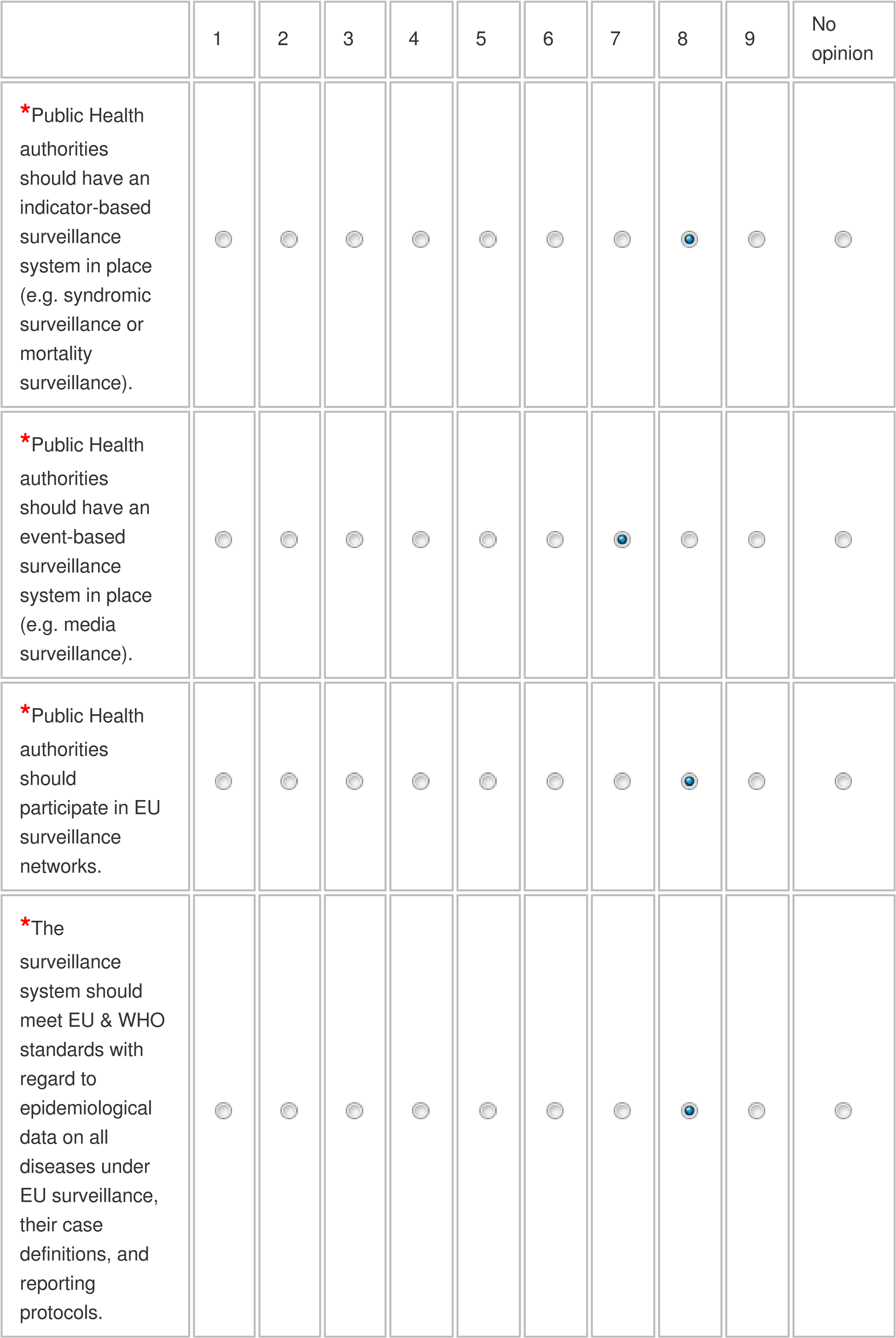


Surveillance

data should be

systematically

and regularly

reported to the

relevant sectors

and

stakeholders.


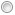

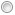

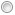

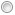

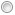

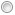

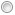

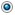

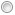

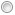


*****

If you have any questions or remarks about the Baseline Set of Indicators in this domain, please write them down here:

If in your opinion other indicators of the complete list (as presented above) should be included in the Baseline Set of Indicators as well, please write the number of this indicator down here:

# 4 - Risk assessment


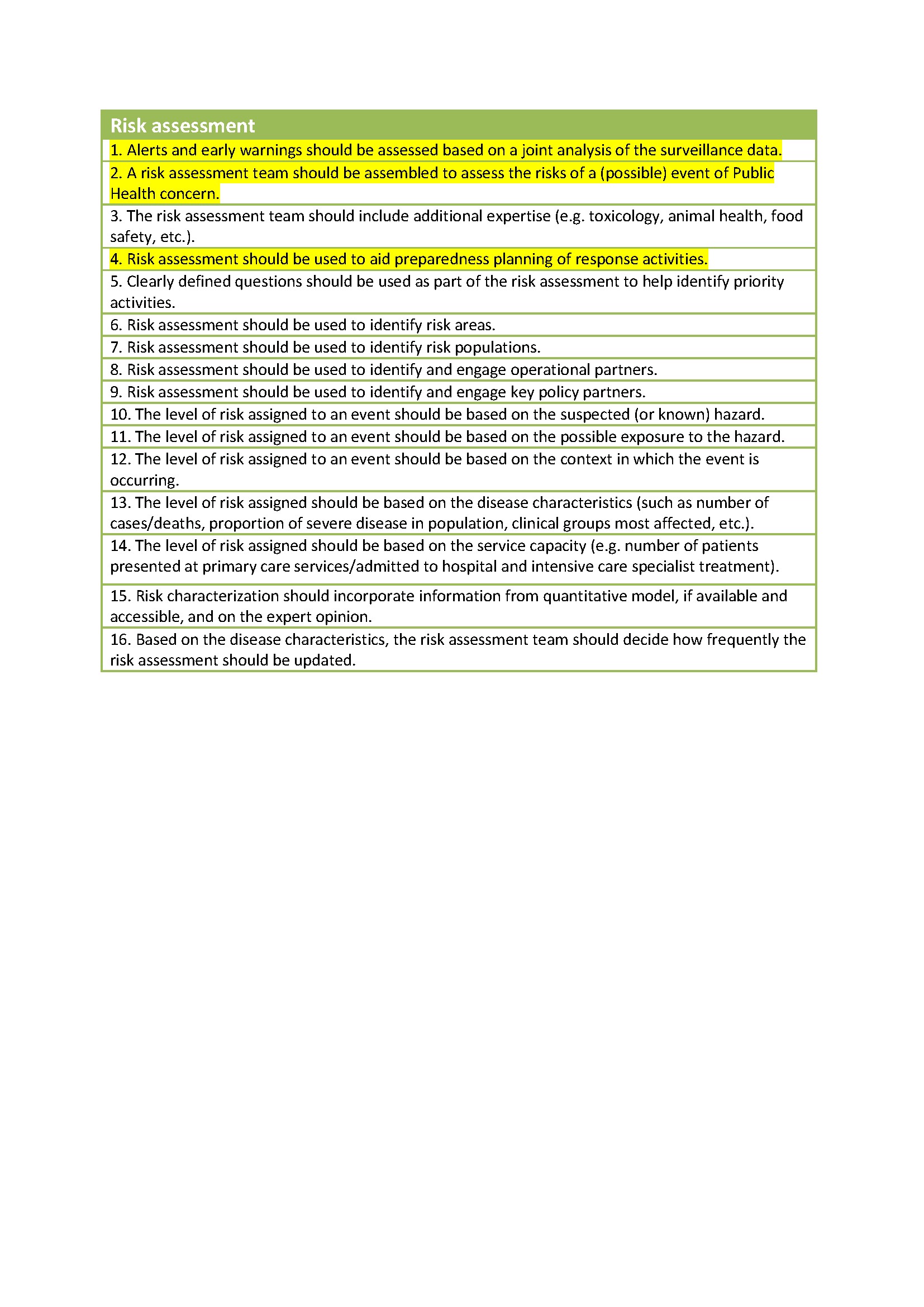


Please indicate below the appropriateness of inlcuding the selected indicators as a **baseline set of indicators, needed to achieve preparedness, applicable for all countries** (1 = not appropriate, 9 = very appropriate). **Score 1 indicates not appropriate and thus exclusion** of the indicator in the baseline set of indicators.


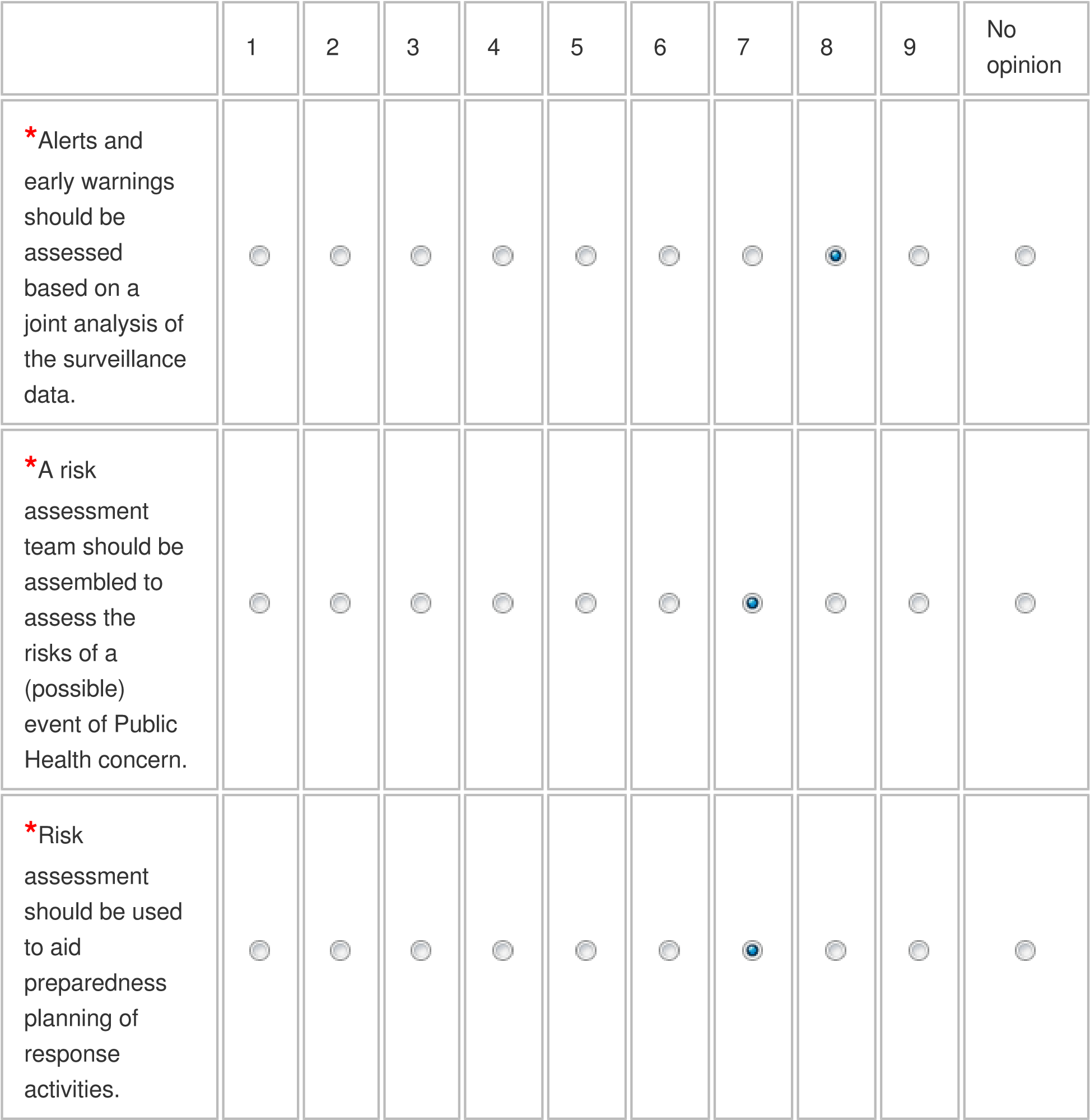


If you have any questions or remarks about the Baseline Set of Indicators in this domain, please write them down here:

If in your opinion other indicators of the complete list (as presented above) should be included in the Baseline Set of Indicators as well, please write the number of this indicator down here:

# 5 - Risk and crisis management


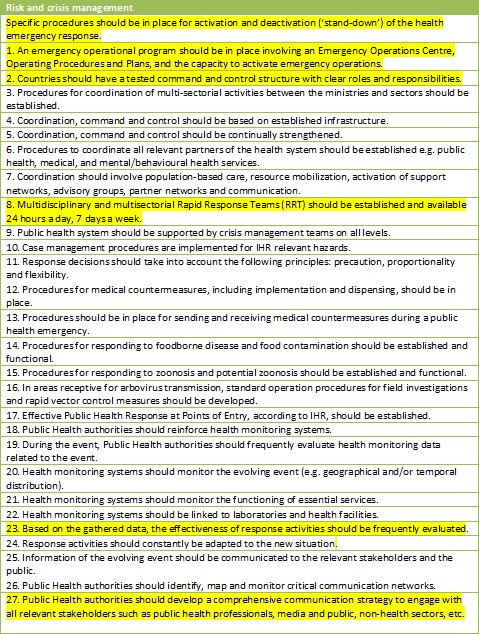


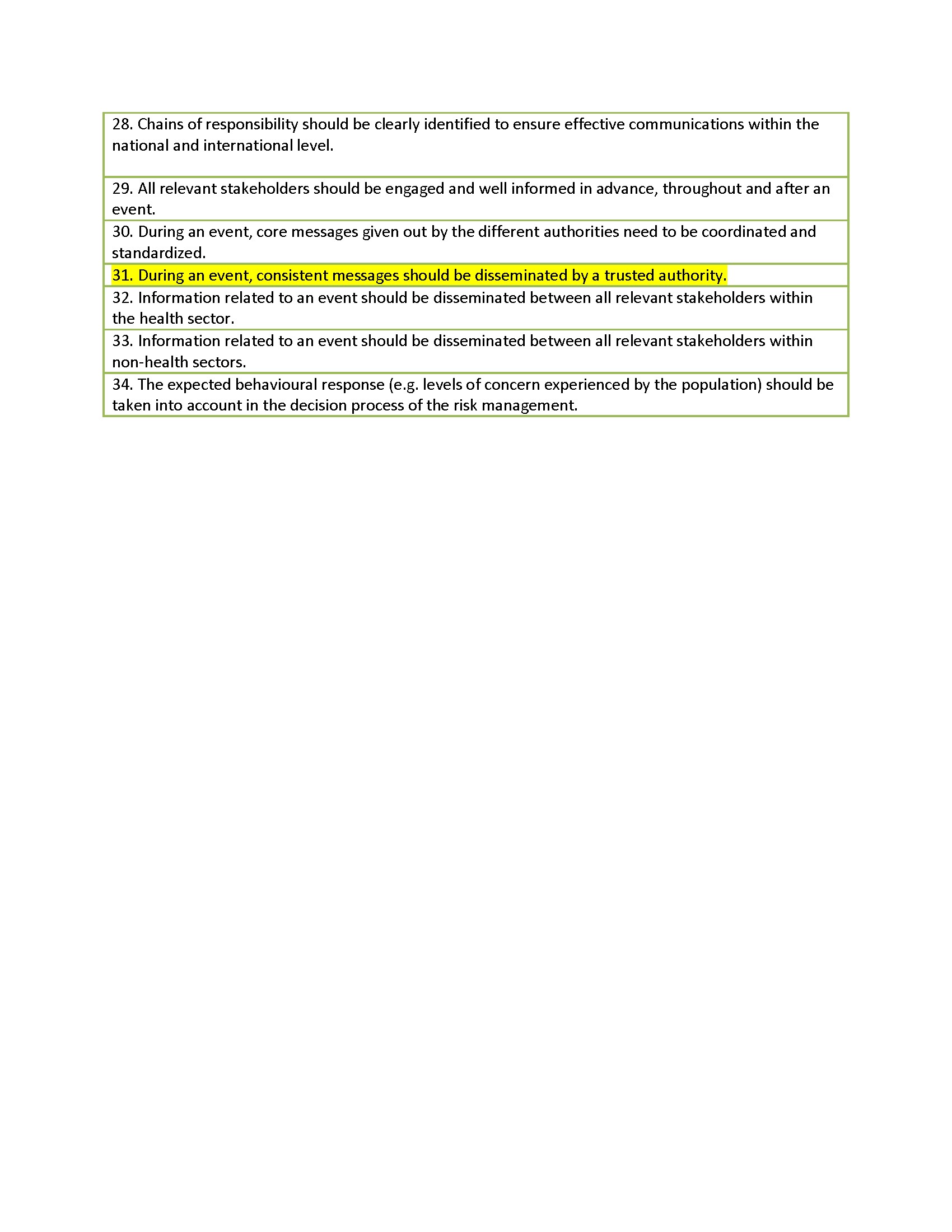


Please indicate below the appropriateness of inlcuding the selected indicators as a **baseline set of indicators, needed to achieve preparedness, applicable for all countries** (1 = not appropriate, 9 = very appropriate). **Score 1 indicates not appropriate and thus exclusion** of the indicator in the baseline set of indicators.

1

2

3

4

5

6

7

8

9

No

opinion


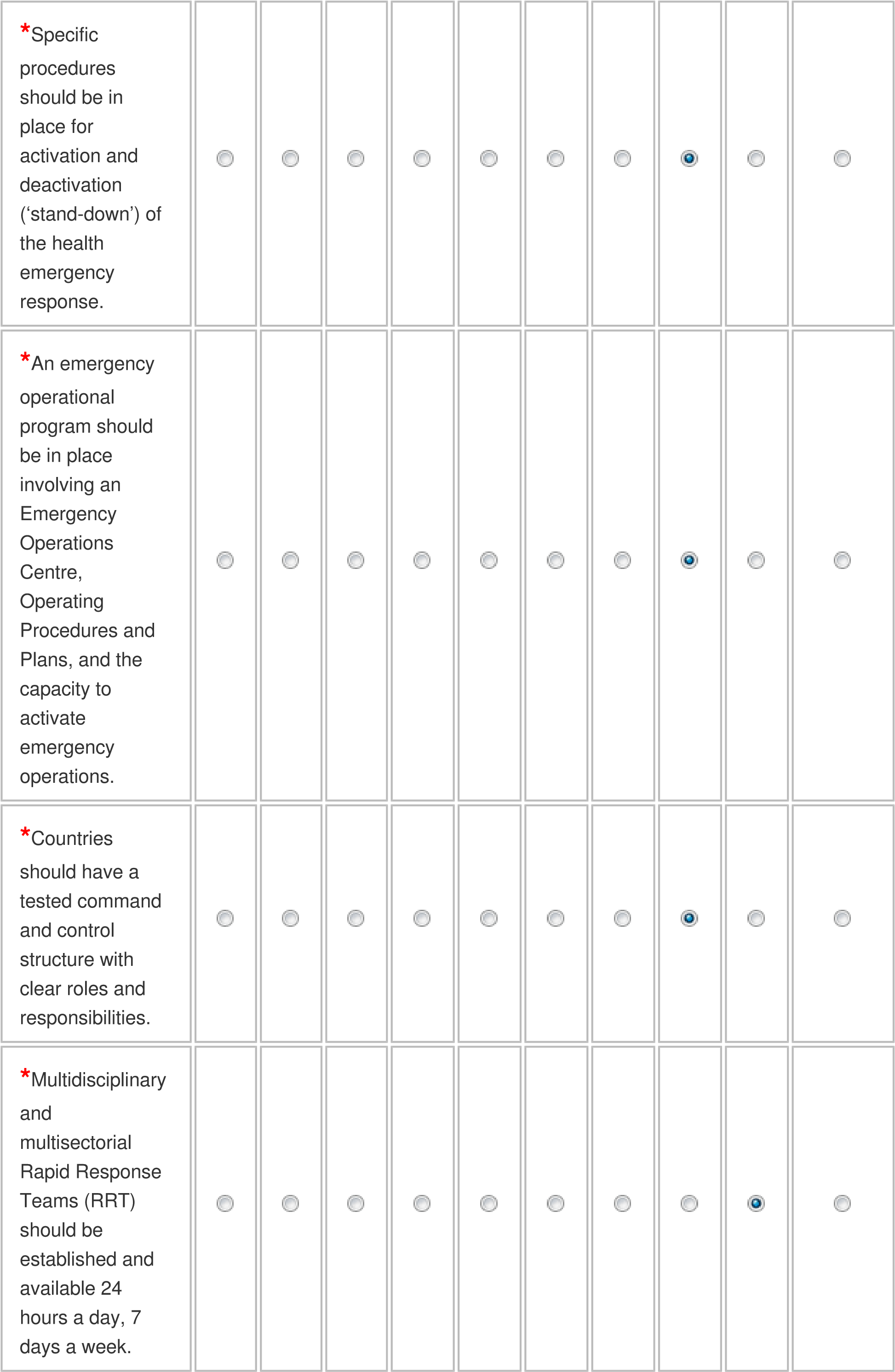


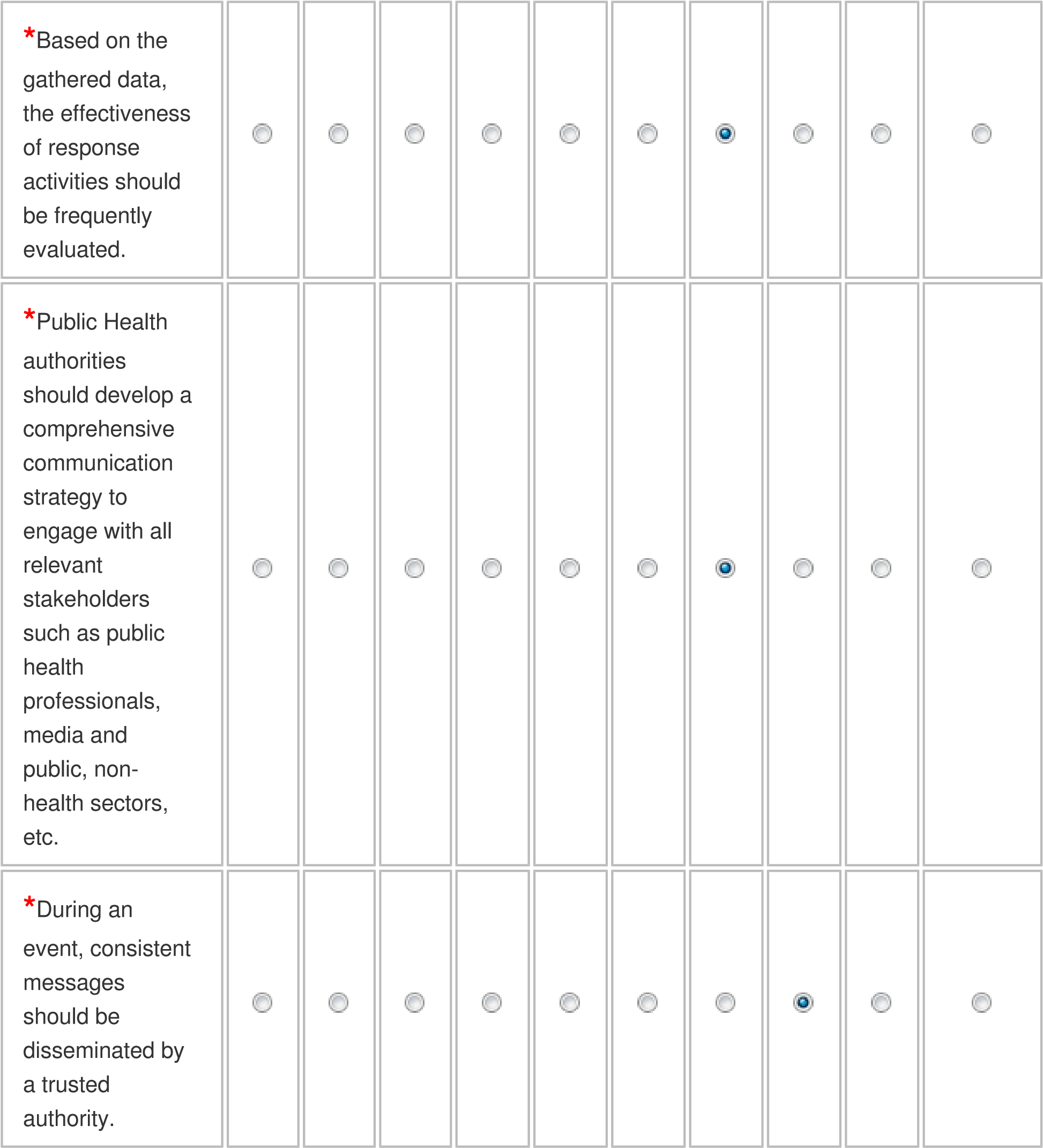


If you have any questions or remarks about the Baseline Set of Indicators in this domain, please write them down here:

If in your opinion other indicators of the complete list (as presented above) should be included in the Baseline Set of Indicators as well, please write the number of this indicator down here:

# 6 - Post-event evaluation


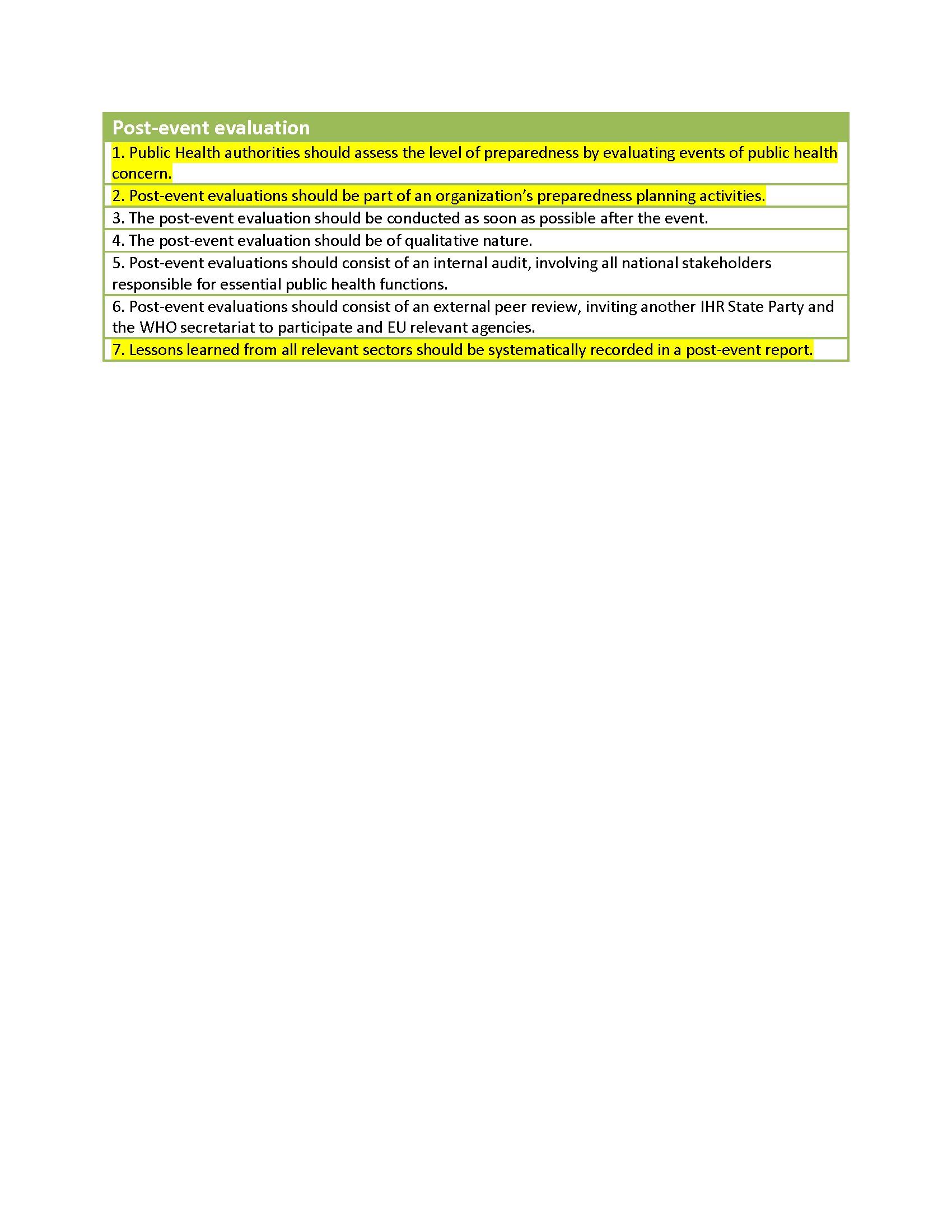


Please indicate below the appropriateness of inlcuding the selected indicators as a **baseline set of indicators, needed to achieve preparedness, applicable for all countries** (1 = not appropriate, 9 = very appropriate). **Score 1 indicates not appropriate and thus exclusion** of the indicator in the baseline set of indicators.


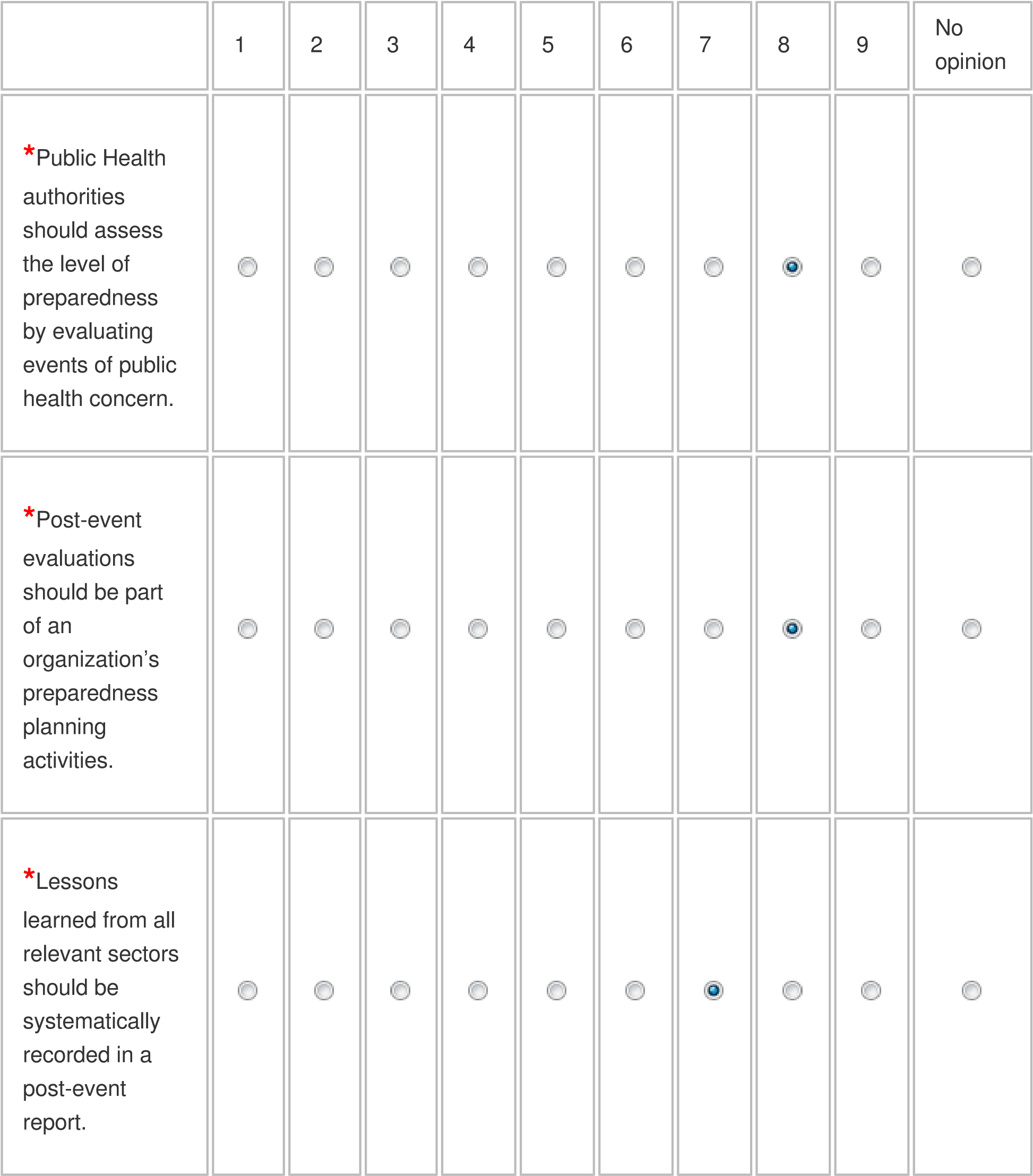


If you have any questions or remarks about the Baseline Set of Indicators in this domain, please write them down here:

If in your opinion other indicators of the complete list (as presented above) should be included in the Baseline Set of Indicators as well, please write the number of this indicator down here:

# 7 - Implementation lessons learned


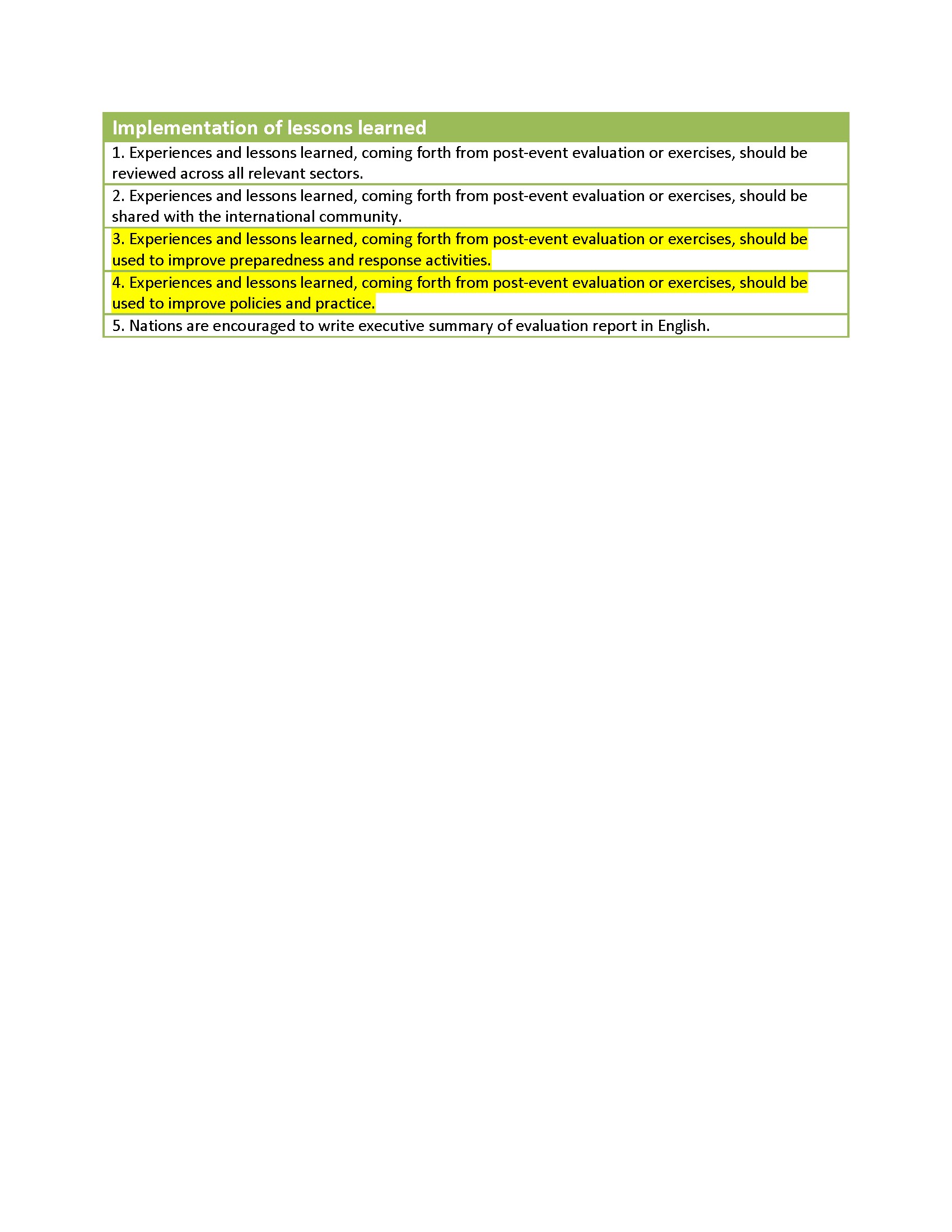


Please indicate below the appropriateness of inlcuding the selected indicators as a **baseline set of indicators, needed to achieve preparedness, applicable for all countries** (1 = not appropriate, 9 = very appropriate). **Score 1 indicates not appropriate and thus exclusion** of the indicator in the baseline set of indicators.


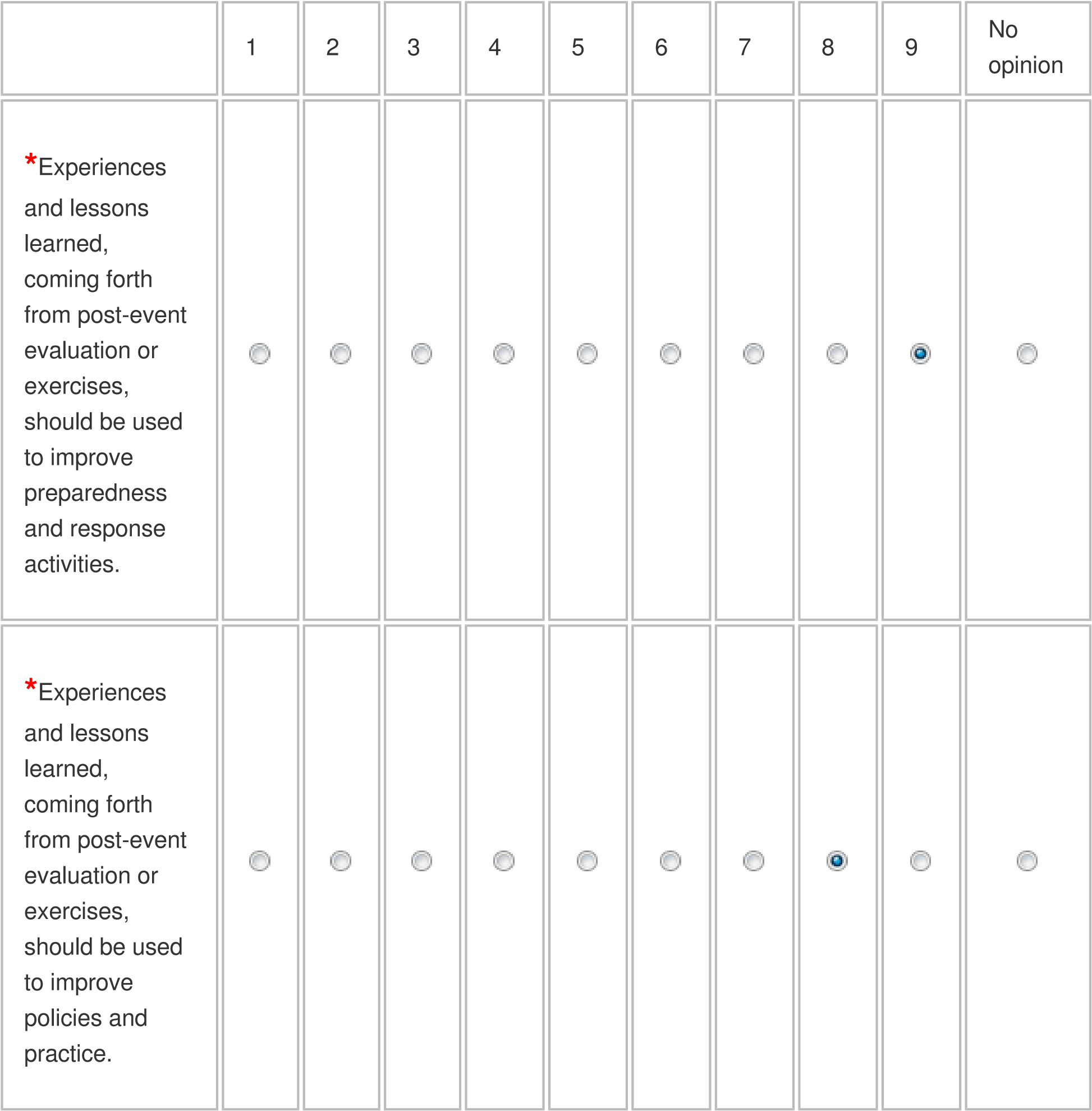


If you have any questions or remarks about the Baseline Set of Indicators in this domain, please write them down here:

If in your opinion other indicators of the complete list (as presented above) should be included in the Baseline Set of Indicators as well, please write the number of this indicator down here:

This is the end of the questionnaire. Thank you very much for your time!

**Contact**

dorothee.rosskamp@rivm.nl
