# Supplementary material for: Towards defining core principles of public health emergency preparedness: scoping review and Delphi consultation among European Union country experts
Source: BMC Public Health. 2020 Oct 1;20:1482. doi: 10.1186/s12889-020-09307-y (PMC7527265; doi:10.1186/s12889-020-09307-y)
Supplement: Supplementary file 3 — Additional file 3:. Core set of recommendations. [file 12889_2020_9307_MOESM3_ESM.docx]

**Additional file 3**

The table in this appendix shows the recommendations and corresponding scores of the panel.

Table 1: Core set of recommendations

| Recommendation | 1 Not relevant | 2 | 3 | 4 | 5 | 6 | 7 | 8 | 9 Very relevant | I don’t know | Median | Percentage 1-3 | Percentage 4-6 | Percentage 7-9 | Conclusion |
| --- | --- | --- | --- | --- | --- | --- | --- | --- | --- | --- | --- | --- | --- | --- | --- |
| Governance |  |  |  |  |  |  |  |  |  |  |  |  |  |  |  |
| 1. A national Public Health Emergency Preparedness Plan should be developed by e.g. National Committees. | 0 | 0 | 1 | 1 | 2 | 0 | 2 | 3 | 18 | 0 | 9 | 3,7% | 11,1% | 85,2% | Selected |
| 2. The national Public Health Emergency Preparedness Plan should be implemented. | 0 | 0 | 0 | 0 | 0 | 0 | 0 | 2 | 25 | 0 | 9 | 0,0% | 0,0% | 100,0% | Selected |
| 3. Preparedness planning should include a self-assessment, involving identification of gaps and possible solutions, human resources capacity, relevant national stakeholders. | 0 | 0 | 0 | 0 | 1 | 1 | 4 | 7 | 14 | 0 | 9 | 0,0% | 7,4% | 92,6% | Selected |
| 4. This self-assessment should be integrated into the existing strategic, planning and financial mechanism. | 0 | 0 | 0 | 1 | 0 | 3 | 4 | 8 | 11 | 0 | 9 | 0,0% | 14,8% | 85,2% | Selected |
| 5. Preparedness planning should include assessing and strengthening existing capacities (structures/services, staff equipment, written plans for preparedness, standard operating procedures). | 0 | 0 | 0 | 0 | 1 | 0 | 3 | 9 | 14 | 0 | 9 | 0,0% | 3,7% | 96,3% | Selected |
| 6. Preparedness planning should include development of appropriate national stockpiles. | 0 | 0 | 0 | 0 | 1 | 3 | 3 | 9 | 11 | 0 | 9 | 0,0% | 14,8% | 85,2% | Selected |
| 7. Preparedness planning should include identification of suppliers for medical countermeasures, including delivery capacity and time. | 0 | 0 | 0 | 0 | 1 | 1 | 6 | 11 | 8 | 0 | 9 | 0,0% | 7,4% | 92,6% | Selected |
| 8. Preparedness planning should include the capacity to support operations at the intermediate and community/primary response levels during a public health emergency. | 0 | 0 | 0 | 1 | 0 | 3 | 2 | 11 | 10 | 0 | 9 | 0,0% | 14,8% | 85,2% | Selected |
| 9. Preparedness planning should include community preparedness to prepare for, resist, and recover from public health incidents. | 0 | 0 | 1 | 0 | 3 | 2 | 6 | 9 | 6 | 0 | 8 | 3,7% | 18,5% | 77,8% | Selected |
| 10. Preparedness should include: the capacity to prevent, detect and manage outbreaks, during large sudden influxes of migrants. | 0 | 0 | 1 | 0 | 3 | 2 | 6 | 9 | 6 | 0 | 9 | 3,7% | 18,5% | 77,8% | Selected |
| 11. Preparedness plans should be flexible and easy adaptable. | 0 | 0 | 0 | 0 | 0 | 0 | 1 | 11 | 15 | 0 | 9 | 0,0% | 0,0% | 100,0% | Selected |
| 12. Preparedness planning should ensure cross-sectorial collaboration and clearly defined roles and responsibilities for all stakeholders. | 0 | 0 | 0 | 0 | 0 | 0 | 1 | 8 | 18 | 0 | 9 | 0,0% | 0,0% | 100,0% | Selected |
| 13. Whole-of-government (i.e. formal and informal networks) biosafety and biosecurity system should be in place for human, animal, and agriculture facilities. | 0 | 0 | 0 | 0 | 1 | 5 | 1 | 11 | 7 | 2 | 9 | 0,0% | 22,2% | 70,4% | Selected |
| 14. Multi-sectorial and multi-stakeholder coordination, command and control should be based on established infrastructure and should be continually strengthened during the planning process. | 0 | 0 | 1 | 0 | 0 | 2 | 2 | 10 | 12 | 0 | 9 | 3,7% | 7,4% | 88,9% | Selected |
| 15. Priority public health risks and resources should be mapped and utilized. | 0 | 0 | 0 | 1 | 2 | 2 | 2 | 12 | 8 | 0 | 9 | 0,0% | 18,5% | 81,5% | Selected |
| 16. Countries should have public health, medical, and mental/behavioural health systems that support recovery. | 0 | 0 | 0 | 0 | 1 | 1 | 8 | 9 | 8 | 0 | 9 | 0,0% | 7,4% | 92,6% | Selected |
| 17. Preparedness plans for events of biological hazards should be in place jointly developed by the public health and non-health sectors such as civil protection, border control and customs. | 0 | 0 | 0 | 0 | 0 | 1 | 5 | 12 | 9 | 0 | 9 | 0,0% | 3,7% | 96,3% | Selected |
| 18. A specific national framework should be in place for priority threats (such as pandemic Influenza) across all sectors. | 0 | 0 | 0 | 0 | 1 | 1 | 6 | 8 | 11 | 0 | 9 | 0,0% | 7,4% | 92,6% | Selected |
| 19. Regarding pandemic preparedness, strong cross-government planning and coordination remains critical and should be led by the Department of Health. | 0 | 1 | 0 | 0 | 1 | 1 | 0 | 10 | 13 | 1 | 9 | 3,7% | 7,4% | 85,2% | Selected |
| 20. The pandemic plans should be consistent with international (e.g. WHO and EU) available guidance. | 0 | 0 | 0 | 0 | 1 | 3 | 3 | 11 | 9 | 0 | 9 | 0,0% | 14,8% | 85,2% | Selected |
| 21. Safety measures for the handling of pathogenic substances should be in place and known by health care workers. | 0 | 0 | 0 | 0 | 1 | 0 | 2 | 8 | 16 | 0 | 9 | 0,0% | 3,7% | 96,3% | Selected |
| 22. Infection prevention and control standards should be established and functioning at national and hospital levels. | 0 | 0 | 0 | 0 | 0 | 1 | 1 | 10 | 15 | 0 | 9 | 0,0% | 3,7% | 96,3% | Selected |
| 23. Antimicrobial stewardship (set of coordinated strategies to improve the use of antimicrobial medications) should be implemented. | 0 | 0 | 0 | 1 | 2 | 2 | 3 | 9 | 10 | 0 | 9 | 0,0% | 18,5% | 81,5% | Selected |
| 24. Laboratory services should be available to test for priority health threats. | 0 | 0 | 0 | 0 | 0 | 1 | 3 | 6 | 17 | 0 | 9 | 0,0% | 3,7% | 96,3% | Selected |
| 25. Laboratory biosafety and laboratory biosecurity (Biorisk management) practices should be in place and implemented. | 0 | 0 | 0 | 0 | 1 | 0 | 2 | 9 | 15 | 0 | 9 | 0,0% | 3,7% | 96,3% | Selected |
| 26. Preparedness should involve national, regional and global networks. | 0 | 0 | 0 | 0 | 0 | 0 | 1 | 12 | 14 | 0 | 9 | 0,0% | 0,0% | 100,0% | Selected |
| 27. Collaboration between countries should be in place to maintain high levels of preparedness. | 0 | 0 | 0 | 0 | 0 | 2 | 3 | 11 | 11 | 0 | 9 | 0,0% | 7,4% | 92,6% | Selected |
| 28. The preparedness and response system for public health emergencies (including communicable diseases) should meet EU best practices. | 0 | 0 | 0 | 0 | 3 | 2 | 5 | 11 | 5 | 1 | 8 | 0,0% | 18,5% | 77,8% | Selected |
| 29. National IHR Focal Points functions and operations should be in place as defined by the IHR (2005). | 0 | 0 | 0 | 0 | 0 | 1 | 2 | 8 | 15 | 1 | 9 | 0,0% | 3,7% | 92,6% | Selected |
| 30. IHR obligations regarding Points of Entry should be fulfilled. | 0 | 0 | 0 | 0 | 0 | 0 | 1 | 14 | 11 | 1 | 9 | 0,0% | 0,0% | 96,3% | Selected |
| 31. Preparedness should be independently evaluated, facilitated by the WHO. | 0 | 1 | 1 | 0 | 3 | 2 | 8 | 6 | 6 | 0 | 8 | 7,4% | 18,5% | 74,1% | Selected |
| 32. Preparedness plans should include a capacity building strategy. | 0 | 0 | 0 | 1 | 1 | 3 | 7 | 8 | 7 | 0 | 9 | 0,0% | 18,5% | 81,5% | Selected |
| 33. Availability of a competent public health workforce for a continuum of health services should be ensured. | 1 | 0 | 0 | 0 | 1 | 2 | 1 | 10 | 11 | 1 | 9 | 3,7% | 11,1% | 81,5% | Selected |
| 34. Human resources should be available to implement IHR core capacity requirements. | 0 | 0 | 0 | 0 | 1 | 1 | 4 | 11 | 10 | 0 | 9 | 0,0% | 7,4% | 92,6% | Selected |
| 35. A system should be in place for medical evacuation of health personnel abroad during a public health emergency. | 0 | 1 | 1 | 1 | 1 | 0 | 8 | 8 | 6 | 1 | 8 | 7,4% | 7,4% | 81,5% | Selected |
| 36. Public Health authorities (i.e. decision-makers)should establish communication policies and procedures to develop, coordinate, and disseminate information related to an event of public health concern. | 0 | 0 | 0 | 1 | 0 | 0 | 2 | 10 | 14 | 0 | 9 | 0,0% | 3,7% | 96,3% | Selected |
| 37. The communication strategy should ensure timely and effective communication before and during an event. | 0 | 0 | 0 | 0 | 0 | 1 | 2 | 6 | 18 | 0 | 9 | 0,0% | 3,7% | 96,3% | Selected |
| 38. The communication strategy should include a scale-up approach. | 0 | 0 | 0 | 0 | 1 | 3 | 5 | 8 | 8 | 2 | 9 | 0,0% | 14,8% | 77,8% | Selected |
| 39. Emergency communication plans should remain flexible and updated as needed. | 0 | 0 | 0 | 1 | 0 | 0 | 2 | 8 | 16 | 0 | 9 | 0,0% | 3,7% | 96,3% | Selected |
| 40. Emergency communication plans should be pragmatic and straightforward to implement. | 0 | 1 | 0 | 0 | 0 | 0 | 4 | 9 | 13 | 0 | 9 | 3,7% | 0,0% | 96,3% | Selected |
| 41. Emergency communications plans should be tested. | 0 | 0 | 1 | 1 | 0 | 1 | 3 | 4 | 17 | 0 | 9 | 3,7% | 7,4% | 88,9% | Selected |
| 42. Emergency communication plans should cover the possibility that certain events receive increased media attention. | 1 | 0 | 0 | 0 | 0 | 2 | 6 | 7 | 11 | 0 | 9 | 3,7% | 7,4% | 88,9% | Selected |
| 43. Emergency communication plans should cover the possibility that certain events lead to a higher demand from the public for information. | 1 | 0 | 0 | 0 | 0 | 4 | 3 | 7 | 12 | 0 | 9 | 3,7% | 14,8% | 81,5% | Selected |
| 44. Information related to an event should be disseminated to the public, in order to explain the outbreak, to establish confidence and to minimize the risk of infection. | 0 | 0 | 0 | 0 | 2 | 0 | 1 | 7 | 16 | 1 | 9 | 0,0% | 7,4% | 88,9% | Selected |
| 45. Communication to the public should be harmonized with other national and international organizations. | 0 | 0 | 0 | 0 | 0 | 4 | 2 | 11 | 10 | 0 | 9 | 0,0% | 14,8% | 85,2% | Selected |
| 46. Public Health authorities should create key messages for public communication. | 0 | 0 | 0 | 0 | 0 | 2 | 3 | 12 | 10 | 0 | 9 | 0,0% | 7,4% | 92,6% | Selected |
| 47. Information to the public should be meaningful, relevant and timely. | 0 | 0 | 0 | 0 | 0 | 0 | 1 | 6 | 20 | 0 | 9 | 0,0% | 0,0% | 100,0% | Selected |
| 48. Information to the public should be open and transparent. | 0 | 0 | 0 | 0 | 0 | 0 | 2 | 6 | 19 | 0 | 9 | 0,0% | 0,0% | 100,0% | Selected |
| 49. Information to the public should be based on a collective perception of risk. | 0 | 0 | 1 | 1 | 0 | 3 | 3 | 7 | 12 | 0 | 9 | 3,7% | 14,8% | 81,5% | Selected |
| 50. Communication to the public should take into account characteristics of the population such as language, social, religious, cultural, political and/or economic aspects. | 0 | 0 | 0 | 0 | 1 | 1 | 2 | 10 | 13 | 0 | 9 | 0,0% | 7,4% | 92,6% | Selected |
| 51. Public Health authorities should set up multiple risk communication channels (e.g. website, E-mail, subject-specific telephone lines). | 0 | 0 | 0 | 1 | 1 | 2 | 2 | 10 | 10 | 1 | 9 | 0,0% | 14,8% | 81,5% | Selected |
| 52. Public Health authorities should provide timely information and guidance about an event to health and other professionals, so they can appropriately respond to the public. | 0 | 0 | 0 | 0 | 0 | 0 | 2 | 9 | 16 | 0 | 9 | 0,0% | 0,0% | 100,0% | Selected |
| 53. Public Health authorities should prepare ad hoc information material for different stakeholders (e.g. simplified case definitions for community use). | 0 | 0 | 0 | 0 | 2 | 3 | 5 | 11 | 6 | 0 | 8 | 0,0% | 18,5% | 81,5% | Selected |
| 54. Public Health organizations (i.e. scientific advisors) should address ethical issues and produce plans for vulnerable populations. | 0 | 0 | 0 | 0 | 3 | 3 | 5 | 6 | 8 | 2 | 9 | 0,0% | 22,2% | 70,4% | Selected |
| 55. Public Health organizations should counter misinformation and prevent stigma, even among educated hospital staff. | 0 | 0 | 0 | 0 | 0 | 2 | 3 | 13 | 9 | 0 | 9 | 0,0% | 7,4% | 92,6% | Selected |
| Capacity building & maintenance (Education, training & simulation exercise) |  |  |  |  |  |  |  |  |  |  |  |  |  |  |  |
| 1. Skills and competences of public health personnel should be strengthened to sustain public health surveillance and response at all levels of the health system. | 0 | 0 | 1 | 0 | 0 | 3 | 1 | 12 | 10 | 0 | 9 | 3,7% | 11,1% | 85,2% | Selected |
| 2. Education, training and exercises should be part of an organization’s preparedness planning activities. | 0 | 0 | 0 | 0 | 1 | 1 | 0 | 11 | 14 | 0 | 9 | 0,0% | 7,4% | 92,6% | Selected |
| 3. Education, training and exercises should be supported at the strategic and operational level of an organization. | 0 | 0 | 0 | 1 | 0 | 2 | 1 | 7 | 16 | 0 | 9 | 0,0% | 11,1% | 88,9% | Selected |
| 4. Public Health authorities should assess the level of preparedness through simulation exercises. | 0 | 0 | 0 | 0 | 1 | 2 | 5 | 9 | 10 | 0 | 9 | 0,0% | 11,1% | 88,9% | Selected |
| 5. Relevant partner organizations should be involved in exercises to improve understanding of each other’s response plans. | 0 | 0 | 0 | 0 | 1 | 1 | 2 | 12 | 11 | 0 | 9 | 0,0% | 7,4% | 92,6% | Selected |
| 6. Simulation exercises should be performed to test procedures for the management of an event (e.g. key roles and decision-making). | 0 | 0 | 0 | 0 | 1 | 0 | 3 | 12 | 11 | 0 | 9 | 0,0% | 3,7% | 96,3% | Selected |
| 7. Exercises should be based on a scenario and tailored to the setting (e.g. local, regional, national, and international). | 0 | 0 | 1 | 0 | 1 | 1 | 2 | 10 | 12 | 0 | 9 | 3,7% | 7,4% | 88,9% | Selected |
| 8. In order to carry out a successful simulation exercise, the planning group should be granted a clear mandate and the authority to plan, conduct and evaluate the exercise. | 0 | 0 | 0 | 0 | 2 | 0 | 2 | 9 | 13 | 1 | 9 | 0,0% | 7,4% | 88,9% | Selected |
| 9. The purpose of a simulation exercise should be to identify areas for improvement. | 0 | 0 | 0 | 0 | 1 | 2 | 2 | 8 | 14 | 0 | 9 | 0,0% | 11,1% | 88,9% | Selected |
| 10. Initial aims and objectives of education, training, and exercises should be evaluated and lessons learned documented in a report. | 0 | 0 | 0 | 0 | 0 | 1 | 3 | 9 | 13 | 1 | 9 | 0,0% | 3,7% | 92,6% | Selected |
| 11. Public Health authorities should conduct exercises to test the actual functionality of IHR capacity. | 0 | 0 | 0 | 2 | 0 | 1 | 3 | 8 | 11 | 2 | 9 | 0,0% | 11,1% | 81,5% | Selected |
| Surveillance |  |  |  |  |  |  |  |  |  |  |  |  |  |  |  |
| 1. Public Health authorities should have an indicator-based surveillance system in place (e.g. syndromic surveillance or mortality surveillance). | 0 | 0 | 0 | 0 | 2 | 0 | 3 | 5 | 16 | 1 | 9 | 0,0% | 7,4% | 88,9% | Selected |
| 2. These indicators should be defined in protocols to enable timely follow-up. | 0 | 0 | 0 | 0 | 1 | 0 | 4 | 9 | 12 | 1 | 9 | 0,0% | 3,7% | 92,6% | Selected |
| 3. Public Health authorities should have an event-based surveillance system in place (e.g. media surveillance). | 0 | 0 | 0 | 1 | 3 | 2 | 2 | 9 | 10 |  | 9 | 0,0% | 22,2% | 77,8% | Selected |
| 4. These events should be defined in protocols, to enable timely follow-up. | 0 | 0 | 2 | 1 | 1 | 0 | 9 | 8 | 5 | 1 | 8 | 7,4% | 7,4% | 81,5% | Selected |
| 5. Public Health authorities should participate in EU surveillance networks. | 0 | 0 | 0 | 0 | 0 | 1 | 3 | 10 | 13 | 0 | 9 | 0,0% | 3,7% | 96,3% | Selected |
| 6. The surveillance system should meet EU & WHO standards with regard to epidemiological data on all diseases under EU surveillance, their case definitions, and reporting protocols. | 0 | 0 | 0 | 0 | 2 | 0 | 2 | 10 | 13 | 0 | 9 | 0,0% | 7,4% | 92,6% | Selected |
| 7. The surveillance system should provide real-time reporting of surveillance data | 0 | 0 | 0 | 0 | 0 | 2 | 2 | 11 | 10 | 2 | 9 | 0,0% | 7,4% | 85,2% | Selected |
| 8. The surveillance system should generate an early warning signal of a possible event of public health concern. | 0 | 0 | 0 | 0 | 0 | 1 | 4 | 8 | 14 | 0 | 9 | 0,0% | 3,7% | 96,3% | Selected |
| 9. The surveillance system should be sensitive and flexible, to detect initial cases or events. | 0 | 0 | 0 | 0 | 1 | 1 | 2 | 12 | 11 | 0 | 9 | 0,0% | 7,4% | 92,6% | Selected |
| 10. The surveillance system should obtain information from a large amount of resources. | 0 | 0 | 0 | 2 | 2 | 2 | 3 | 9 | 5 | 4 | 8 | 0,0% | 22,2% | 63,0% | Selected |
| 11. The surveillance system should be able to provide the information necessary to inform and advice response. | 0 | 0 | 0 | 0 | 1 | 2 | 2 | 9 | 12 | 1 | 9 | 0,0% | 11,1% | 85,2% | Selected |
| 12. The surveillance network should include information from veterinary surveillance systems. | 0 | 0 | 0 | 0 | 1 | 2 | 5 | 10 | 7 | 2 | 9 | 0,0% | 11,1% | 81,5% | Selected |
| 13. The surveillance network should include information from entomological surveillance systems. | 0 | 0 | 0 | 0 | 4 | 2 | 4 | 8 | 6 | 3 | 9 | 0,0% | 22,2% | 66,7% | Selected |
| 14. The surveillance network should include information from environmental surveillance systems. | 0 | 0 | 0 | 2 | 1 | 3 | 6 | 7 | 6 | 2 | 9 | 0,0% | 22,2% | 70,4% | Selected |
| 15. The surveillance network should include information from meteorological surveillance systems. | 0 | 1 | 1 | 2 | 2 | 1 | 7 | 6 | 5 | 2 | 8 | 7,4% | 18,5% | 66,7% | Selected |
| 16. The surveillance network should include information from microbiological surveillance systems. | 0 | 0 | 0 | 0 | 0 | 1 | 3 | 10 | 12 | 1 | 9 | 0,0% | 3,7% | 92,6% | Selected |
| 17. All relevant surveillance systems should be integrated in a network that consistently exchanges information. | 0 | 0 | 1 | 0 | 0 | 3 | 5 | 11 | 7 | 0 | 8 | 3,7% | 11,1% | 85,2% | Selected |
| 18. Surveillance data should be systematically and regularly reported to the relevant sectors and stakeholders. | 0 | 0 | 0 | 0 | 0 | 0 | 0 | 14 | 13 | 0 | 9 | 0,0% | 0,0% | 100,0% | Selected |
| 19. Public Health authorities should have reporting networks and protocols in place | 0 | 0 | 0 | 0 | 0 | 1 | 2 | 14 | 10 | 0 | 9 | 0,0% | 3,7% | 96,3% | Selected |
| Risk assessment |  |  |  |  |  |  |  |  |  |  |  |  |  |  |  |
| 1. Alerts and early warnings should be assessed based on a joint analysis of the surveillance data. | 0 | 0 | 0 | 0 | 0 | 0 | 1 | 14 | 11 | 1 | 9 | 0,0% | 0,0% | 96,3% | Selected |
| 2. A risk assessment team should be assembled to assess the risks of a (possible) event of Public Health concern. | 0 | 0 | 0 | 1 | 0 | 0 | 2 | 10 | 13 | 1 | 9 | 0,0% | 3,7% | 92,6% | Selected |
| 3. The risk assessment team should include additional expertise (e.g. toxicology, animal health, food safety, etc.). | 0 | 0 | 0 | 0 | 0 | 1 | 3 | 11 | 10 | 2 | 9 | 0,0% | 3,7% | 88,9% | Selected |
| 4. Risk assessment should be used to aid preparedness planning of response activities. | 0 | 0 | 0 | 0 | 1 | 3 | 0 | 11 | 12 | 0 | 9 | 0,0% | 14,8% | 85,2% | Selected |
| 5. Clearly defined questions should be used as part of the risk assessment to help identify priority activities. | 0 | 0 | 0 | 0 | 1 | 1 | 4 | 10 | 9 | 2 | 9 | 0,0% | 7,4% | 85,2% | Selected |
| 6. Risk assessment should be used to identify risk areas. | 0 | 0 | 0 | 0 | 1 | 1 | 3 | 11 | 11 | 0 | 9 | 0,0%% | 7,4% | 92,6% | Selected |
| 7. Risk assessment should be used to identify risk populations. | 0 | 0 | 0 | 0 | 0 | 1 | 2 | 11 | 13 | 0 | 9 | 0,0% | 3,7% | 96,3% | Selected |
| 8. Risk assessment should be used to identify and engage operational partners. | 0 | 0 | 0 | 0 | 2 | 3 | 6 | 10 | 5 | 1 | 8 | 0,0% | 18,5% | 77,8% | Selected |
| 9. Risk assessment should be used to identify and engage key policy partners. | 0 | 0 | 0 | 1 | 3 | 1 | 5 | 11 | 5 | 1 | 8 | 0,0%% | 18,5% | 77,8% | Selected |
| 10. The level of risk assigned to an event should be based on the suspected (or known) hazard. | 0 | 0 | 0 | 0 | 0 | 2 | 5 | 9 | 11 | 0 | 9 | 0,0% | 7,4% | 92,6% | Selected |
| 11. The level of risk assigned to an event should be based on the possible exposure to the hazard. | 0 | 0 | 0 | 0 | 1 | 1 | 3 | 11 | 11 | 0 | 9 | 0,0% | 7,4% | 92,6% | Selected |
| 12. The level of risk assigned to an event should be based on the context in which the event is occurring. | 0 | 0 | 0 | 0 | 0 | 0 | 5 | 14 | 8 | 0 | 9 | 0,0% | 0,0% | 100,0% | Selected |
| 13. The level of risk assigned should be based on the disease characteristics (such as number of cases/deaths, proportion of severe disease in population, clinical groups most affected, etc.). | 0 | 0 | 0 | 0 | 0 | 0 | 2 | 11 | 13 | 10 | 9 | 0,0% | 0,0% | 72,2% | Selected |
| 14. The level of risk assigned should be based on the service capacity (e.g. number of patience presented at primary care services/admitted to hospital and intensive care specialist treatment). | 0 | 0 | 1 | 0 | 0 | 2 | 3 | 12 | 8 | 1 | 9 | 3,7% | 7,4% | 85,2% | Selected |
| 15. The level of risk assigned should be based on the expected behavioural response (e.g. levels of concern experienced by the population). | 0 | 1 | 1 | 0 | 2 | 3 | 8 | 9 | 3 | 0 | 7 | 7,4% | 18,5% | 74,1% | Discussion |
| 16. Risk characterization should be based primary on quantitative model and secondary on the expert opinion of the team. | 1 | 0 | 0 | 2 | 3 | 1 | 5 | 6 | 5 | 4 | 8 | 3,7% | 22,2% | 59,3% | Discussion |
| 17. Based on the disease characteristics, the risk assessment team should decide how frequently the risk assessment should be updated. | 0 | 0 | 0 | 0 | 2 | 1 | 7 | 10 | 7 | 0 | 8 | 0,0% | 11,1% | 88,9% | Selected |
| Risk and crisis management |  |  |  |  |  |  |  |  |  |  |  |  |  |  |  |
| 1. An emergency operational program should be in place involving an Emergency Operations Centre, Operating Procedures and Plans, and the capacity to activate emergency operations. | 0 | 0 | 0 | 0 | 0 | 1 | 1 | 8 | 17 | 0 | 9 | 0,0% | 3,7% | 96,3% | Selected |
| 2. Countries should have a tested command and control structure with clear roles and responsibilities. | 0 | 0 | 0 | 0 | 0 | 1 | 0 | 8 | 18 | 0 | 9 | 0,0% | 3,7% | 96,3% | Selected |
| 3. Procedures for coordination of multi-sectorial activities between the ministries and sectors should be established. | 0 | 0 | 0 | 0 | 0 | 0 | 2 | 10 | 15 | 0 | 9 | 0,0% | 0,0% | 100,0% | Selected |
| 4. Coordination, command and control should be based on established infrastructure. | 0 | 0 | 0 | 0 | 0 | 1 | 2 | 10 | 14 | 0 | 9 | 0,0% | 3,7% | 96,3% | Selected |
| 5. Coordination, command and control should be continually strengthened. | 0 | 1 | 0 | 0 | 1 | 1 | 3 | 9 | 11 | 1 | 9 | 3,7% | 7,4% | 85,2% | Selected |
| 6. Procedures to coordinate all relevant partners of the health system should be established e.g. public health, medical, and mental/behavioural health services. | 0 | 0 | 0 | 0 | 1 | 0 | 7 | 6 | 13 | 0 | 9 | 0,0% | 3,7% | 96,3% | Selected |
| 7. Coordination should involve population-based care, resource mobilization, activation of support networks, advisory groups, partner networks and communication. | 0 | 0 | 0 | 0 | 2 | 0 | 4 | 9 | 11 | 1 | 9 | 0,0% | 7,4% | 88,9% | Selected |
| 8. Multidisciplinary and multisectorial Rapid Response Teams (RRT) should be established and available 24 hours a day, 7 days a week. | 1 | 0 | 0 | 1 | 2 | 1 | 5 | 4 | 12 | 1 | 9 | 3,7% | 14,8% | 77,8% | Selected |
| 9. Public health system should be supported by crisis management teams on all levels. | 0 | 0 | 1 | 1 | 2 | 2 | 4 | 9 | 8 | 0 | 9 | 3,7% | 18,5% | 77,8% | Selected |
| 10. Case management procedures are implemented for IHR relevant hazards. | 0 | 1 | 0 | 0 | 1 | 1 | 2 | 12 | 8 | 2 | 9 | 3,7% | 7,4% | 81,5% | Selected |
| 11. Response decisions should take into account the following principles: precaution, proportionality and flexibility. | 0 | 0 | 0 | 0 | 1 | 1 | 3 | 9 | 12 | 1 | 9 | 0,0% | 7,4% | 88,9% | Selected |
| 12. Procedures for medical countermeasures, including implementation and dispensing, should be in place. | 0 | 0 | 0 | 0 | 1 | 1 | 1 | 13 | 10 | 1 | 9 | 0,0% | 7,4% | 88,9% | Selected |
| 13. Procedures should be in place for sending and receiving medical countermeasures during a public health emergency. | 0 | 0 | 0 | 0 | 1 | 0 | 0 | 16 | 9 | 1 | 9 | 0,0% | 3,7% | 92,6% | Selected |
| 14. Procedures for responding to foodborne disease and food contamination should be established and functional. | 0 | 0 | 0 | 0 | 0 | 0 | 3 | 12 | 12 | 0 | 9 | 0,0% | 0,0% | 100,0% | Selected |
| 15. Procedures for responding to zoonosis and potential zoonosis should be established and functional. | 0 | 0 | 0 | 0 | 0 | 0 | 3 | 12 | 12 | 0 | 9 | 0,0% | 0,0% | 100,0% | Selected |
| 16. In areas receptive for arbovirus transmission, standard operation procedures for field investigations and rapid vector control measures should be developed. | 0 | 0 | 1 | 0 | 0 | 0 | 4 | 14 | 6 | 2 | 8 | 3,7% | 0,0% | 88,9% | Selected |
| 17. Effective Public Health Response at Points of Entry, according to IHR, should be established. | 0 | 0 | 0 | 0 | 0 | 0 | 3 | 8 | 15 | 1 | 9 | 0,0% | 0,0% | 96,3% | Selected |
| 18. Public Health authorities should reinforce health monitoring systems. | 0 | 0 | 0 | 0 | 2 | 1 | 3 | 9 | 11 | 1 | 9 | 0,0% | 11,1% | 85,2% | Selected |
| 19. During the event, Public Health authorities should frequently evaluate health monitoring data related to the event. | 0 | 0 | 0 | 0 | 0 | 1 | 0 | 14 | 12 | 0 | 9 | 0,0% | 3,7% | 96,3% | Selected |
| 20. Health monitoring systems should monitor the evolving event (e.g. geographical and/or temporal distribution). | 0 | 0 | 0 | 0 | 0 | 0 | 5 | 10 | 12 | 0 | 9 | 0,0% | 0,0% | 100,0% | Selected |
| 21. Health monitoring systems should monitor the functioning of essential services. | 0 | 0 | 0 | 0 | 2 | 2 | 3 | 12 | 7 | 1 | 9 | 0,0% | 14,8% | 81,5% | Selected |
| 22. Health monitoring systems should be linked to laboratories and health facilities. | 0 | 0 | 0 | 0 | 0 | 0 | 1 | 10 | 14 | 2 | 9 | 0,0% | 0,0% | 92,6% | Selected |
| 23. Based on the gathered data, the effectiveness of response activities should be frequently evaluated. | 0 | 0 | 0 | 0 | 1 | 0 | 3 | 12 | 11 | 0 | 9 | 0,0% | 3,7% | 96,3% | Selected |
| 24. Response activities should constantly be adapted to the new situation. | 0 | 0 | 0 | 0 | 1 | 0 | 1 | 11 | 14 | 0 | 9 | 0,0% | 3,7% | 96,3%% | Selected |
| 25. Information of the evolving event should be communicated to the relevant stakeholders and the public. | 0 | 0 | 0 | 0 | 0 | 0 | 0 | 12 | 15 | 0 | 9 | 0,0% | 0,0% | 100,0% | Selected |
| 26. Public Health authorities should identify, map and monitor critical communication networks. | 0 | 0 | 0 | 0 | 1 | 1 | 4 | 11 | 8 | 2 | 9 | 0,0% | 7,4% | 85,2% | Selected |
| 27. Public Health authorities should develop a comprehensive communication strategy to engage with all relevant stakeholders such as public health professionals, media and public, non-health sectors, etc. | 0 | 0 | 0 | 0 | 0 | 1 | 2 | 14 | 10 | 0 | 9 | 0,0% | 3,7% | 96,3% | Selected |
| 28. Chains of responsibility should be clearly identified to ensure effective communications within the national and international level. | 0 | 0 | 0 | 0 | 0 | 0 | 2 | 10 | 15 | 0 | 9 | 0,0% | 0,0% | 100,0% | Selected |
| 29. All relevant stakeholders should be engaged and well informed in advance, throughout and after an event. | 0 | 0 | 0 | 0 | 0 | 0 | 0 | 14 | 12 | 1 | 9 | 0,0% | 0,0% | 96,3% | Selected |
| 30. During an event, core messages given out by the different authorities need to be coordinated and standardized. | 0 | 0 | 0 | 0 | 0 | 0 | 0 | 14 | 13 | 0 | 9 | 0,0% | 0,0% | 100,0% | Selected |
| 31. During an event, consistent messages should be disseminated by a trusted authority. | 0 | 0 | 0 | 0 | 0 | 0 | 1 | 8 | 18 | 0 | 9 | 0,0% | 0,0% | 100,0% | Selected |
| 32. Information related to an event should be disseminated between all relevant stakeholders within the health sector. | 0 | 0 | 0 | 0 | 0 | 1 | 0 | 12 | 14 | 0 | 9 | 0,0% | 3,7% | 96,3% | Selected |
| 33. Information related to an event should be disseminated between all relevant stakeholders within non-health sectors. | 0 | 0 | 0 | 0 | 0 | 2 | 2 | 14 | 9 | 0 | 9 | 0,0% | 7,4% | 92,6% | Selected |
| Post-event evaluation |  |  |  |  |  |  |  |  |  |  |  |  |  |  |  |
| 1. Public Health authorities should assess the level of preparedness by evaluating events of public health concern. | 0 | 0 | 0 | 0 | 0 | 0 | 3 | 14 | 10 | 0 | 9 | 0,0% | 0,0% | 100,0% | Selected |
| 2. Post-event evaluations should be part of an organization’s preparedness planning activities. | 0 | 0 | 0 | 0 | 0 | 1 | 3 | 9 | 14 | 0 | 9 | 0,0% | 3,7% | 96,3% | Selected |
| 3. The post-event evaluation should be conducted as soon as possible after the event. | 0 | 0 | 1 | 0 | 0 | 1 | 3 | 11 | 11 | 0 | 9 | 3,7% | 3,7% | 92,6% | Selected |
| 4. The post-event evaluation should be conducted on a national level. | 0 | 0 | 2 | 0 | 3 | 3 | 3 | 7 | 8 | 1 | 9 | 7,4% | 22,2% | 66,7% | Discussion |
| 5. The post-event evaluation should be of qualitative nature. | 0 | 0 | 1 | 1 | 2 | 1 | 4 | 8 | 7 | 3 | 9 | 3,7% | 14,8% | 70,4% | Selected |
| 6. Post-event evaluations should consist of an internal audit, involving all national stakeholders responsible for essential public health functions. | 0 | 1 | 0 | 0 | 1 | 1 | 8 | 10 | 5 | 1 | 8 | 3,7% | 7,4% | 85,2% | Selected |
| 7. Post-event evaluations should consist of an external peer review, inviting another IHR State Party and the WHO secretariat to participate and EU relevant agencies. | 1 | 2 | 1 | 2 | 4 | 2 | 3 | 7 | 3 | 2 | 7 | 14,8% | 29,6% | 48,1% | Not Selected |
| 8. Lessons learned from all relevant sectors should be systematically recorded in a post-event report. | 0 | 0 | 1 | 0 | 0 | 1 | 3 | 10 | 11 | 1 | 9 | 3,7% | 3,7% | 88,9% | Selected |
| Implementation of lessons learned |  |  |  |  |  |  |  |  |  |  |  |  |  |  |  |
| 1. Experiences and lessons learned, coming forth from post-event evaluation or exercises, should be reviewed across all relevant sectors. | 0 | 0 | 0 | 0 | 0 | 1 | 0 | 12 | 14 | 0 | 9 | 0,0% | 3,7% | 96,3% | Selected |
| 2. Experiences and lessons learned, coming forth from post-event evaluation or exercises, should be shared with the international community. | 0 | 0 | 1 | 0 | 2 | 4 | 8 | 8 | 4 | 0 | 8 | 3,7% | 22,2 | 74,1% | Selected |
| 3. Experiences and lessons learned, coming forth from post-event evaluation or exercises, should be used to improve preparedness and response activities. | 0 | 0 | 0 | 0 | 0 | 0 | 0 | 11 | 16 | 0 | 9 | 0,0% | 0,0% | 100,0% | Selected |
| 4. Experiences and lessons learned, coming forth from post-event evaluation or exercises, should be used to improve policies and practice. | 0 | 0 | 0 | 0 | 0 | 0 | 1 | 9 | 17 | 0 | 9 | 0,0% | 0,0% | 100,0% | Selected |
