# Supplementary material for: Towards defining core principles of public health emergency preparedness: scoping review and Delphi consultation among European Union country experts
Source: BMC Public Health. 2020 Oct 1;20:1482. doi: 10.1186/s12889-020-09307-y (PMC7527265; doi:10.1186/s12889-020-09307-y)
Supplement: Supplementary file 4 — Additional file 4:. Baseline (prioritized) set of recommendations. [file 12889_2020_9307_MOESM4_ESM.docx]

**Additional file 4**

The table in this appendix shows the preselected recommendations and corresponding scores of the panel.

| Recommendation | 1 Not relevant | 2 | 3 | 4 | 5 | 6 | 7 | 8 | 9 Very relevant | No opinion | Median | Percentage 1-3 | Percentage 4-6 | Percentage 7-9 | Conclusion |
| --- | --- | --- | --- | --- | --- | --- | --- | --- | --- | --- | --- | --- | --- | --- | --- |
| Governance |  |  |  |  |  |  |  |  |  |  |  |  |  |  |  |
| Emergency preparedness should be integrated in national health strategies, financing and plans.* | 0 | 0 | 0 | 0 | 0 | 0 | 1 | 5 | 6 | 0 | 9 | 0,0% | 0,0% | 100,0% | Selected |
| Multi-sectoral emergency risk management policies and legislation include public health treats.* | 0 | 1 | 0 | 1 | 0 | 0 | 2 | 4 | 4 | 0 | 8 | 8,3% | 8,3% | 83,3% | Selected |
| 1. A national Public Health Emergency Preparedness Plan should be developed, kept updated or endorsed by e.g. National Competent Body. | 0 | 0 | 1 | 0 | 3 | 0 | 1 | 6 | 12 | 0 | 9 | 4,3% | 13,0% | 82,6% | Selected |
| 3. Preparedness planning should include a self-assessment, involving identification of gaps and possible solutions, human  resources capacity, relevant national stakeholders. | 0 | 0 | 1 | 2 | 0 | 0 | 6 | 7 | 7 | 0 | 8 | 4,3% | 8,7% | 87,0% | Selected |
| 5. Preparedness planning should include assessing and strengthening existing capacities (structures/services, staff equipment, written plans for preparedness, standard operating procedures). | 0 | 0 | 1 | 1 | 0 | 0 | 4 | 9 | 7 | 0 | 8 | 4,5% | 4,5% | 90,9% | Selected |
| 12. Preparedness planning should ensure cross-sectorial collaboration and clearly defined roles and responsibilities for all stakeholders. | 0 | 0 | 0 | 0 | 1 | 1 | 2 | 5 | 14 | 0 | 9 | 0,0% | 8,7% | 91,3% | Selected |
| 15. Priority public health risks and resources should be mapped and utilized. | 0 | 0 | 0 | 1 | 2 | 3 | 4 | 4 | 9 | 0 | 8 | 0,0% | 26,1% | 73,9% | Selected |
| 18. A specific national framework should be in place for priority threats (such as pandemic Influenza) across all sectors. | 0 | 0 | 0 | 0 | 0 | 2 | 4 | 6 | 10 | 0 | 8 | 0,0% | 9,1% | 90,9% | Selected |
| 24. Laboratory services should be available to test for priority health threats. | 0 | 0 | 0 | 0 | 2 | 0 | 4 | 2 | 15 | 1 | 9 | 0,0% | 8,3% | 87,5% | Selected |
| 26. Preparedness should involve national, regional and global networks. | 0 | 0 | 0 | 0 | 2 | 1 | 4 | 9 | 7 | 1 | 8 | 0,0% | 12,5% | 83,3% | Selected |
| 27. Collaboration between countries should be in place to maintain high levels of preparedness. | 0 | 0 | 0 | 0 | 1 | 1 | 5 | 10 | 5 | 0 | 8 | 0,0% | 9,1% | 90,9% | Selected |
| 29. National IHR Focal Points functions and operations should be in place as defined by the IHR (2005). | 0 | 0 | 0 | 0 | 0 | 0 | 1 | 6 | 15 | 0 | 9 | 0,0% | 0,0% | 100,0% | Selected |
| 36. Public Health authorities (i.e. decision-makers) should establish communication policies and procedures to develop, coordinate, and disseminate information related to an event of public health concern. | 0 | 0 | 0 | 0 | 0 | 0 | 3 | 5 | 14 | 0 | 9 | 0,0% | 0,0% | 100,0% | Selected |
| 44. Information related to an event should be disseminated to the public, in order to explain the outbreak, to establish confidence and to minimize the risk of infection. | 0 | 0 | 0 | 0 | 0 | 2 | 2 | 8 | 11 | 0 | 8 | 0,0% | 8,7% | 91,3% | Selected |
| Developing competencies (Education, training & simulation exercise) |  |  |  |  |  |  |  |  |  |  |  |  |  |  |  |
| 1. Skills and competences of public health personnel should be strengthened to sustain public health surveillance and response at all levels of the health system. | 0 | 0 | 0 | 0 | 1 | 2 | 3 | 7 | 9 | 0 | 8 | 0,0% | 13,6% | 86,4% | Selected |
| 3. Education, training and exercises should be part of an organization’s preparedness planning activities. | 0 | 0 | 0 | 0 | 0 | 0 | 1 | 6 | 16 | 0 | 9 | 0,0% | 0,0% | 100,0% | Selected |
| 4. Public Health authorities should assess the level of preparedness through simulation exercises. | 0 | 0 | 0 | 1 | 1 | 0 | 4 | 8 | 8 | 0 | 8 | 0,0% | 9,1% | 90,9% | Selected |
| 10. Initial aims and objectives of education, training, and exercises should be evaluated and lessons learned documented in a report. | 0 | 1 | 1 | 0 | 0 | 3 | 6 | 6 | 6 | 0 | 8 | 8,7% | 13,0% | 78,3% | Selected |
| Surveillance |  |  |  |  |  |  |  |  |  |  |  |  |  |  |  |
| 1. Public Health authorities should have an indicator-based surveillance system in place (e.g. syndromic surveillance or mortality surveillance). | 0 | 0 | 0 | 1 | 0 | 0 | 1 | 9 | 12 | 0 | 9 | 0,0% | 4,3% | 95,7% | Selected |
| 3. Public Health authorities should have an event-based surveillance system in place (e.g. media surveillance). | 0 | 0 | 1 | 1 | 2 | 3 | 4 | 5 | 7 | 0 | 8 | 4,3% | 26,1% | 69,6% | Discussion |
| 5. Public Health authorities should participate in EU surveillance networks. | 0 | 0 | 0 | 0 | 0 | 1 | 1 | 6 | 14 | 0 | 9 | 0,0% | 4,5% | 95,5% | Selected |
| 6. The surveillance system should meet EU & WHO standards with regard to epidemiological data on all diseases under EU surveillance, their case definitions, and reporting protocols. | 0 | 0 | 0 | 0 | 0 | 1 | 3 | 8 | 11 | 0 | 8 | 0,0% | 4,3% | 95,7% | Selected |
| 18. Surveillance data should be systematically and regularly reported to the relevant sectors and stakeholders. | 0 | 0 | 1 | 0 | 0 | 0 | 0 | 7 | 15 | 0 | 9 | 4,3% | 0,0% | 95,7% | Selected |
| Risk assessment |  |  |  |  |  |  |  |  |  |  |  |  |  |  |  |
| 1. Alerts and early warnings should be assessed based on a joint analysis of the surveillance data. | 0 | 0 | 0 | 0 | 0 | 0 | 1 | 8 | 14 | 0 | 9 | 0,0% | 0,0% | 100,0% | Selected |
| 2. A risk assessment team should be assembled to assess the risks of a (possible) event of Public Health concern. | 0 | 0 | 0 | 0 | 1 | 2 | 4 | 6 | 10 | 0 | 8 | 0,0% | 13,0% | 87,0% | Selected |
| 4. Risk assessment should be used to aid preparedness planning of response activities. | 0 | 0 | 0 | 0 | 0 | 0 | 1 | 9 | 13 | 0 | 9 | 0,0% | 0,0% | 100,0% | Selected |
| Risk and crisis management |  |  |  |  |  |  |  |  |  |  |  |  |  |  |  |
| Specific procedures should be in place for activation and deactivation (‘stand-down’) of the health emergency response.* | 0 | 0 | 0 | 0 | 0 | 2 | 1 | 4 | 4 | 0 | 8 | 0,0% | 18,2% | 81,8% | Selected |
| 1. An emergency operational program should be in place involving an Emergency Operations Centre, Operating Procedures and Plans, and the capacity to activate emergency operations. | 0 | 0 | 0 | 0 | 0 | 0 | 4 | 6 | 13 | 0 | 9 | 0,0% | 0,0% | 100,0% | Selected |
| 2. Countries should have a tested command and control structure with clear roles and responsibilities. | 0 | 0 | 0 | 0 | 0 | 1 | 1 | 7 | 14 | 0 | 9 | 0,0% | 4,3% | 95,7% | Selected |
| 8. Multidisciplinary and multisectorial Rapid Response Teams (RRT) should be established and available 24 hours a day, 7 days a week. | 2 | 0 | 0 | 0 | 2 | 3 | 4 | 4 | 8 | 0 | 8 | 8,7% | 21,7% | 69,6% | Discussion |
| 23. Based on the gathered data, the effectiveness of response activities should be frequently evaluated. | 1 | 0 | 1 | 0 | 0 | 0 | 8 | 8 | 5 | 1 | 8 | 8,3% | 0,0% | 87,5% | Selected |
| 27. Public Health authorities should develop a comprehensive communication strategy to engage with all relevant stakeholders such as public health professionals, media and public, non-health sectors, etc. | 0 | 0 | 0 | 1 | 0 | 0 | 4 | 8 | 9 | 1 | 8 | 0,0% | 4,3% | 91,3% | Selected |
| 31. During an event, consistent messages should be disseminated by a trusted authority. | 0 | 1 | 0 | 1 | 0 | 0 | 2 | 4 | 14 | 0 | 9 | 4,5% | 4,5% | 90,9% | Selected |
| Post-event evaluation |  |  |  |  |  |  |  |  |  |  |  |  |  |  |  |
| 1. Public Health authorities should assess the level of preparedness by evaluating events of public health concern. | 0 | 0 | 0 | 0 | 1 | 0 | 1 | 8 | 13 | 0 | 9 | 0,0% | 4,3% | 95,7% | Selected |
| 2. Post-event evaluations should be part of an organization’s preparedness planning activities. | 0 | 0 | 1 | 0 | 0 | 0 | 3 | 5 | 13 | 0 | 9 | 4,5% | 0,0% | 95,5% | Selected |
| 7. Lessons learned from all relevant sectors should be systematically recorded in a post-event report. | 0 | 1 | 0 | 0 | 1 | 0 | 4 | 4 | 13 | 0 | 9 | 4,3% | 4,3% | 91,3% | Selected |
| Implementation of lessons learned |  |  |  |  |  |  |  |  |  |  |  |  |  |  |  |
| 3. Experiences and lessons learned, coming forth from post-event evaluation or exercises, should be used to improve preparedness and response activities. | 0 | 0 | 0 | 0 | 1 | 0 | 0 | 5 | 17 | 0 | 9 | 0,0% | 4,3% | 95,7% | Selected |
| 4. Experiences and lessons learned, coming forth from post-event evaluation or exercises, should be used to improve policies and practice. | 0 | 0 | 0 | 1 | 0 | 0 | 2 | 5 | 14 | 1 | 9 | 0,0% | 4,3% | 91,3% | Selected |

*** These recommendations were assessed by about half of the expert panel because they were added to the digital questionnaire when some expert already finished the questionnaire.**
