# Supplementary material for: Towards defining core principles of public health emergency preparedness: scoping review and Delphi consultation among European Union country experts
Source: BMC Public Health. 2020 Oct 1;20:1482. doi: 10.1186/s12889-020-09307-y (PMC7527265; doi:10.1186/s12889-020-09307-y)
Supplement: Supplementary file 5 — Additional file 5:. Interview guide step 3. [file 12889_2020_9307_MOESM5_ESM.pdf]

Fine-tuning of recommendations

Set of recommendations for inclusion in the handbook

Aim

to reach consensus on recommendations that were not directly accepted in the questionnaire

- Discuss new recommendations
- Discuss textual alternations based on comments provided
- Open disucssion on unclear definitions

What do we ask from you

- To help us with refining the recommendations in a group discussion
- Use of a voting system for small textual alterations of recommendations which are already accepted

Bricks

	Items to decide on
• Preparedness	6
• Capacity building and maintenance	1
• Surveillance	3
• Risk assessment	5
• Risk and crisis management	5
• Post-event evaluation	3
• Implementation of lessons learned	1
	24

Preparedness (1/6)

Recommendation 1:
A national Public Health Emergency Preparedness Plan should be developed by e.g. National Committees.

Comment: Only developed or also endorsed?

Proposal: ***A national Public Health Emergency Preparedness Plan should be developed or endorsed by e.g. National Committees.***

Do you endorse this adjustment?

A national Public Health Emergency Preparedness Plan should be developed or endorsed by e.g. National Committees.

A. Yes

B. No

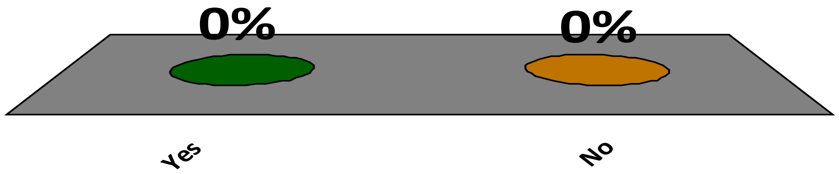

Preparedness (2/6)

T

Recommendation 35:
A system should be in place for medical evacuation of health personnel abroad during a public health emergency.

Comment: Not clear, whom it concerns exactly.

Proposal: ***For health personnel that are assisting in a public health emergency abroad, a system should be in place for medical evacuation.***

Do you endorse this adjustment?

For health personnel that are assisting in a public health emergency abroad, a system should be in place for medical evacuation.

A. Yes

B. No

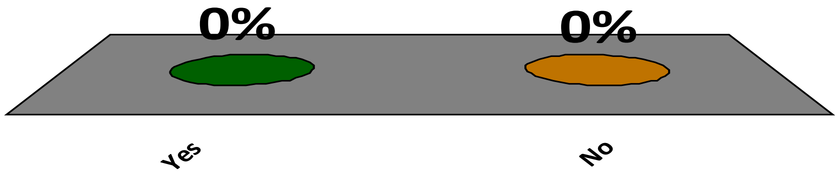

Preparedness (3/6)

T

Recommendation 47:
Information to the public should be meaningful relevant and timely.

Comments: Information to the public has to be true.

Proposal: ***Information to the public should be meaningful, transparent, relevant and timely.***

Do you endorse this adjustment?

Information to the public should be meaningful, transparent, relevant and timely.

A. Yes

B. No

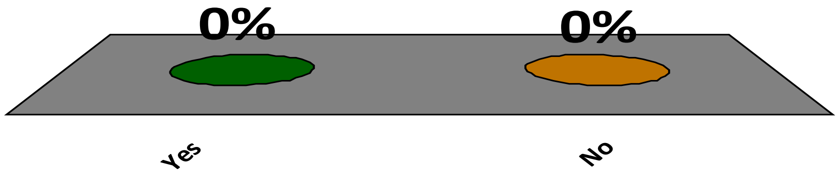

Preparedness (4/6)

T

Recommendation 49:
Information to the public should be based on a collective perception of risk.

Comments: This depends on the outbreak.

Proposal: ***Information to the public should be based on real-time monitoring of risk perceptions.***

Do you endorse this adjustment?

Information to the public should be based on real-time monitoring of risk perceptions.

A. Yes

B. No

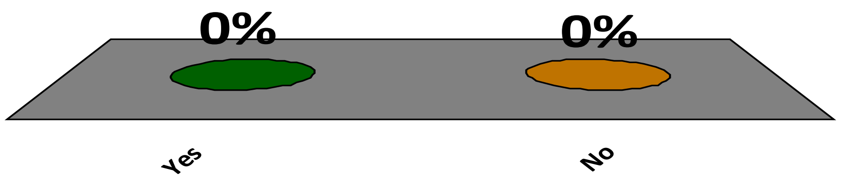

Preparedness (5/6)

T

Recommendation 54:
Public Health organizations (i.e. scientific advisors) should address ethical issues and produce plans for vulnerable populations.

Comments: This should be integrated in the existing plan. In addition, vulnerable populations should be specified.

Proposal: ***Public Health organizations (i.e. scientific advisors) should address ethical issues and integrate vulnerable populations (e.g. children, pregnant women, elderly people, malnourished people, and people who are ill or immunocompromised) in their preparedness plan.***

Do you endorse this adjustment?

Public Health organizations (i.e. scientific advisors) should address ethical issues and integrate vulnerable populations (e.g. children, pregnant women, elderly people, malnourished people, and people who are ill or immunocompromised) in their preparedness plan.

A. Yes

B. No

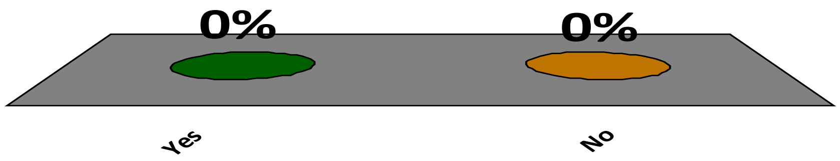

Preparedness (6/6)

N

General comment: It should inlcude an additional recommendation regarding focal points of specific vulnerable groups.

New recommendation: ***Focal points of specific vulnerable populations should be identified.***

Open for discussion

Do you endorse this new recommendation?

Focal points of specific vulnerable populations should be identified.

A. Yes
B. No

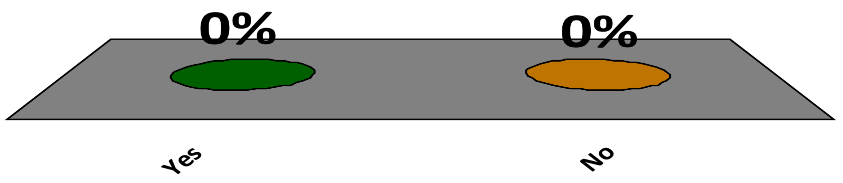

Capacity building & maintenance (1/1)

Recommendation 11:
Public Health authorities should conduct exercises to test the actual functionality of IHR capacity.

Comment: This is very broad.

Proposal: Public Health authorities should conduct exercises to test the actual functionality of IHR core capacities.

Do you endorse this adjustment?

Public Health authorities should conduct exercises to test the actual functionality of IHR core capacities.

A. Yes
B. No

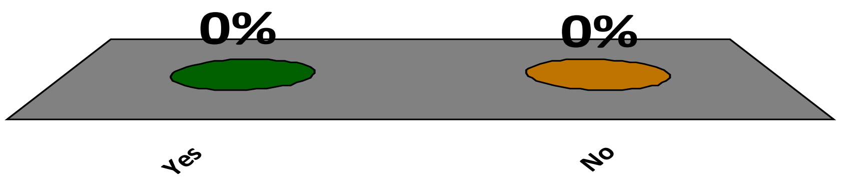

Surveillance (1/3)

O

Recommendation 7:
The surveillance system should provide real-time reporting of surveillance data

Comment: Define ‘real-time’.

Question: Do we have to define ‘real-time’ and how?

Open for discussion

Surveillance (2/3)

T + O

Recommendation 10:
The surveillance system should obtain information from a large amount of resources.

Comments: Clarify what kind of resources.

Proposal: ***The surveillance system should obtain information from a broad range of different and reliable resources.***

Question: How can we specify the different resources?

Open for discussion

Do you endorse this adjustment?

The surveillance system should obtain information from a broad range of different and reliable resources.

A. Yes

B. No

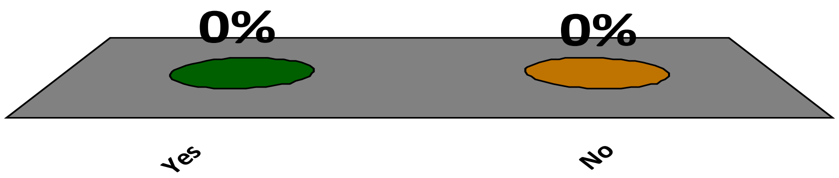

Question: How can we specify the different resources?

Open for discussion

Surveillance (3/3)

o

Recommendation 12:
The surveillance network should include information from veterinary surveillance systems.

Recommendation 13:
The surveillance network should include information from entomological surveillance systems.

Recommendation 14:
The surveillance network should include information from environmental surveillance systems.

Recommendation 14:
The surveillance network should include information from meterological surveillance systems.

Comments: I don't know. Please define 'surveillance network'.

Question: Do we have to define 'surveillance network' and how?

Open for discussion

Risk assessment (1/5)

T

Recommendation 10:
The level of risk assigned to an event should be based on the suspected (or known) hazard.

Comments: It is not only based on the hazard. In addition, it also depends on the severity of the hazard.

Proposals: ***The level of risk assigned to an event should, among other, be based on the severity of the suspected (or known) hazard.***

Do you endorse this adjustment?

The level of risk assigned to an event should, among other, be based on the severity of the suspected (or known) hazard.

A. Yes

B. No

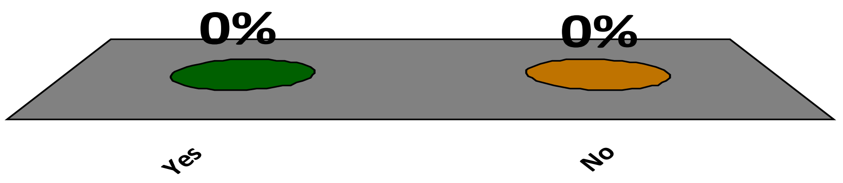

Risk assessment (2/5)

T

Recommendation 11:
The level of risk assigned to an event should be based on the possible exposure to the hazard.

Comments: It is not only based on the hazard.

Proposal: ***The level of risk assigned to an event should, among other, be based on the possible exposure to the hazard.***

Do you endorse this adjustment?

The level of risk assigned to an event should, among other, be based on the possible exposure to the hazard.

A. Yes

B. No

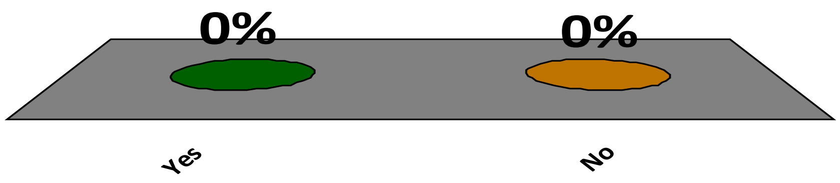

T

Risk assessment (3/5)

Recommendation 13:
The level of risk assigned should be based on the disease characteristics (such as number of cases/deaths, proportion of severe disease in population, clinical groups most affected, etc.).

Comments: Not only disease, but also outbreak.

Proposal: ***The level of risk assigned should be based on the disease and outbreak characteristics (such as number of cases/deaths, proportion of severe disease in population, clinical groups most affected, etc.).***

Do you endorse this adjustment?

The level of risk assigned should be based on the disease and outbreak characteristics (such as number of cases/deaths, proportion of severe disease in population, clinical groups most affected, etc.).

A. Yes
B. No

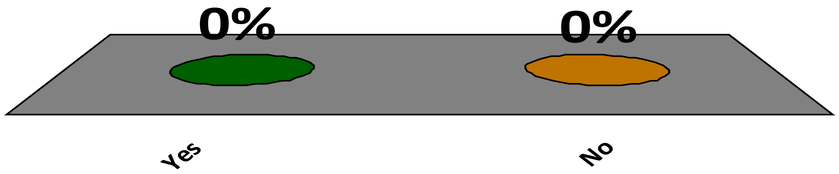

Risk assessment (4/5)

D

Recommendation 15:
The level of risk assigned should be based on the expected behavioral response (e.g. levels of concern experienced by the population).

Comments: This is difficult to measure and anticipate. This should be based on evidence not behavioral.

Open for discussion

Risk assessment (5/5)

D

Recommendation 16:
Risk characterization should be based primary on quantitative model and secondary on the expert opinion of the team.

Comments: Models are too much based on assumptions and are arbitrary. Agree for the most part, however, most of the time not fully understood.

Open for discussion

o

Risk and crisis management (1/5)

Recommendation 8:
Multidisciplinary and multisectorial Rapid Response Teams (RRT) should be established and available 24 hours a day, 7 days a week.

Comment: It is not clear what RRT means.

This recommendation is accepted, however: Open for discussion if more clarification is needed.

o

Risk and crisis management (2/5)

Recommendation 16:
In areas receptive for arbovirus transmission, standard operation procedures for field investigations and rapid vector control measures should be developed.

Comment: This should be for all vector borne diseases.

This recommendation is accepted, however: Open for discussion if all vector borne diseases should be included.

T

Risk and crisis management (3/5)

Recommendation 22:
Health monitoring systems should be linked to laboratories and health facilities.

Comments: Not clear. Define ‘linked’.

Proposal: ***Health monitoring systems should receive input from and give feedback to laboratories and health facilities.***

Do you endorse this adjustment?

Health monitoring systems should receive input from and give feedback to laboratories and health facilities.

A. Yes

B. No

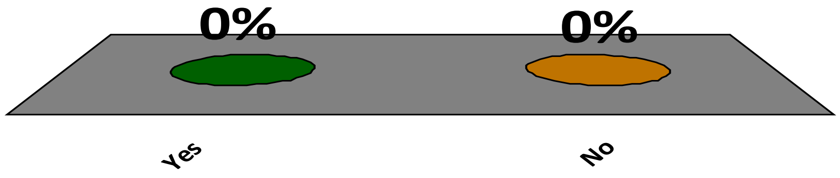

Risk and crisis management (4/5)

o

Recommendation 26:
Public Health authorities should identify, map and monitor critical communication networks.

Comment: Define critical communication networks

Question: Do we have to define 'critical communication networks' and how?

Open for discussion

Risk and crisis management (5/5)

General comment: The set of recommendations should include a recommendation regarding decision-making

New recommendation: ***Response decisions should be based on available resources, capacities, capabilities and support.***

Do you endorse this new recommendation?

Response decisions should be based on available resources, capacities, capabilities and support.

A. Yes

B. No

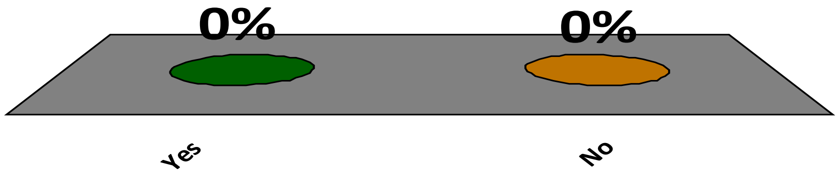

Post-event evaluation (1/3)

o

Recommendation 5:
The post-event evaluation should be of qualitative nature.

Comments: Probably mostly the best, however not only qualitative. Both qualitative as quantitative are relevant.

This recommendation is accepted, however: Open for discussion if quantitative should be included.

Post-event evaluation (2/3)

Recommendation 4:
The post-event evaluation should be conducted on a national level.

Comments: It should and must have external partners. It depends on the event.

Open for discussion

Post-event evaluation (3/3)

General comment: Evaluation should be more stadardized and systematic

New recommendation: ***Post-event evaluation should consist of a systematic approach with standardized indicators related to the characteristics of the event.***

Do you endorse this adjustment?

Post-event evaluation should consist of a systematic approach with standardized indicators related to the characteristics of the event.

A. Yes

B. No

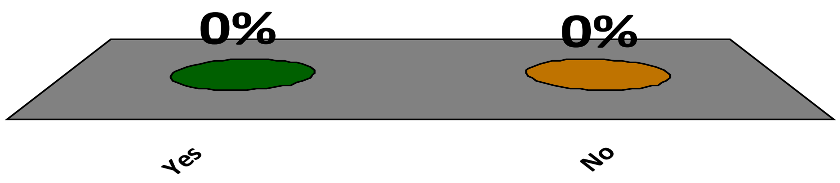

O

Implementation of lessons learned (1/1)

General comment: Nations should not be obliged to write evaluation reports in English.

Open for discussion
