# Supplementary material for: Towards defining core principles of public health emergency preparedness: scoping review and Delphi consultation among European Union country experts
Source: BMC Public Health. 2020 Oct 1;20:1482. doi: 10.1186/s12889-020-09307-y (PMC7527265; doi:10.1186/s12889-020-09307-y)
Supplement: Supplementary file 6 — Additional file 6:. Interview guide step 6. [file 12889_2020_9307_MOESM6_ESM.pptx]

## Slide 1
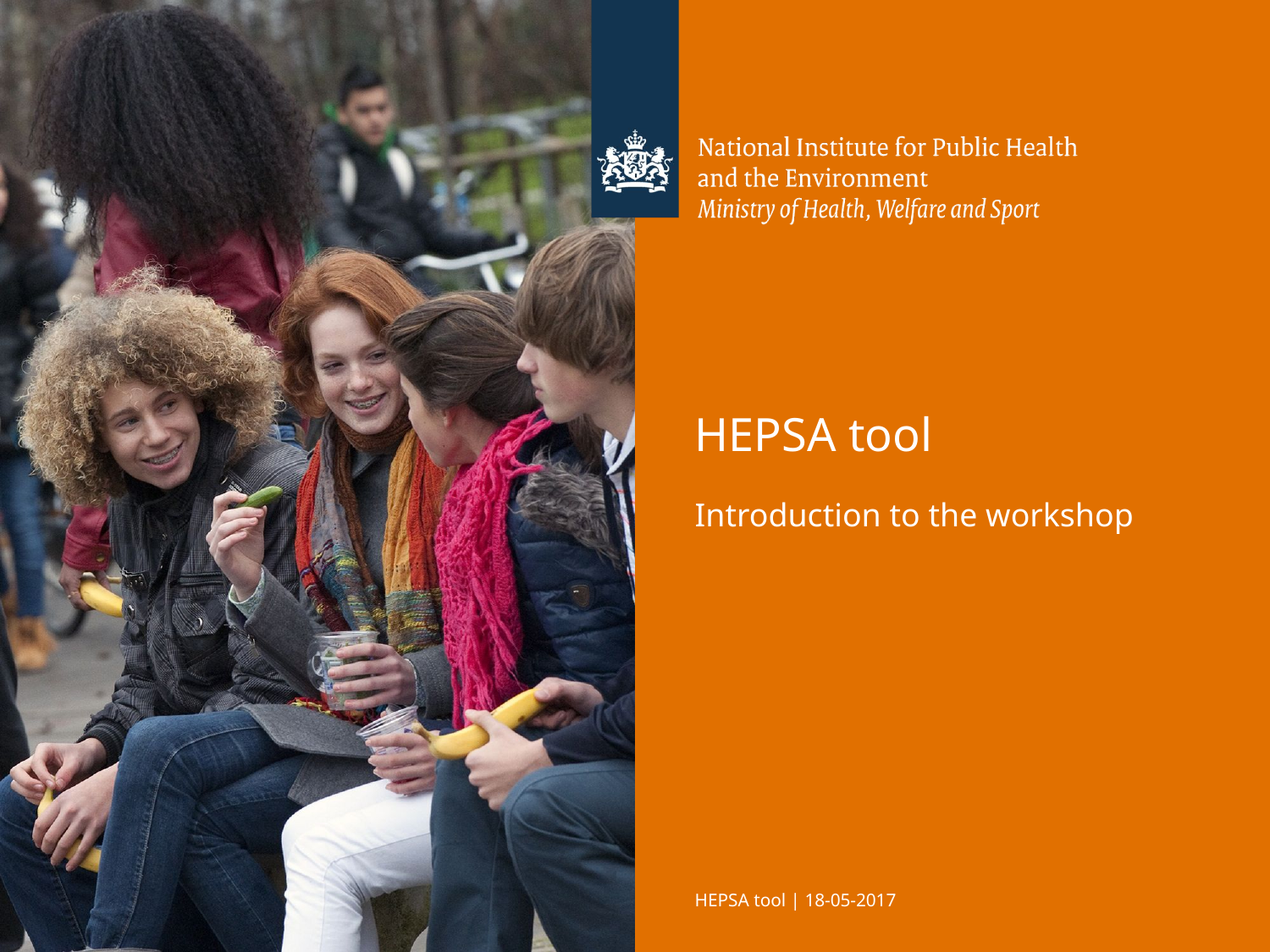

# HEPSA tool
Introduction to the workshop
HEPSA tool | 18-05-2017

## Slide 2
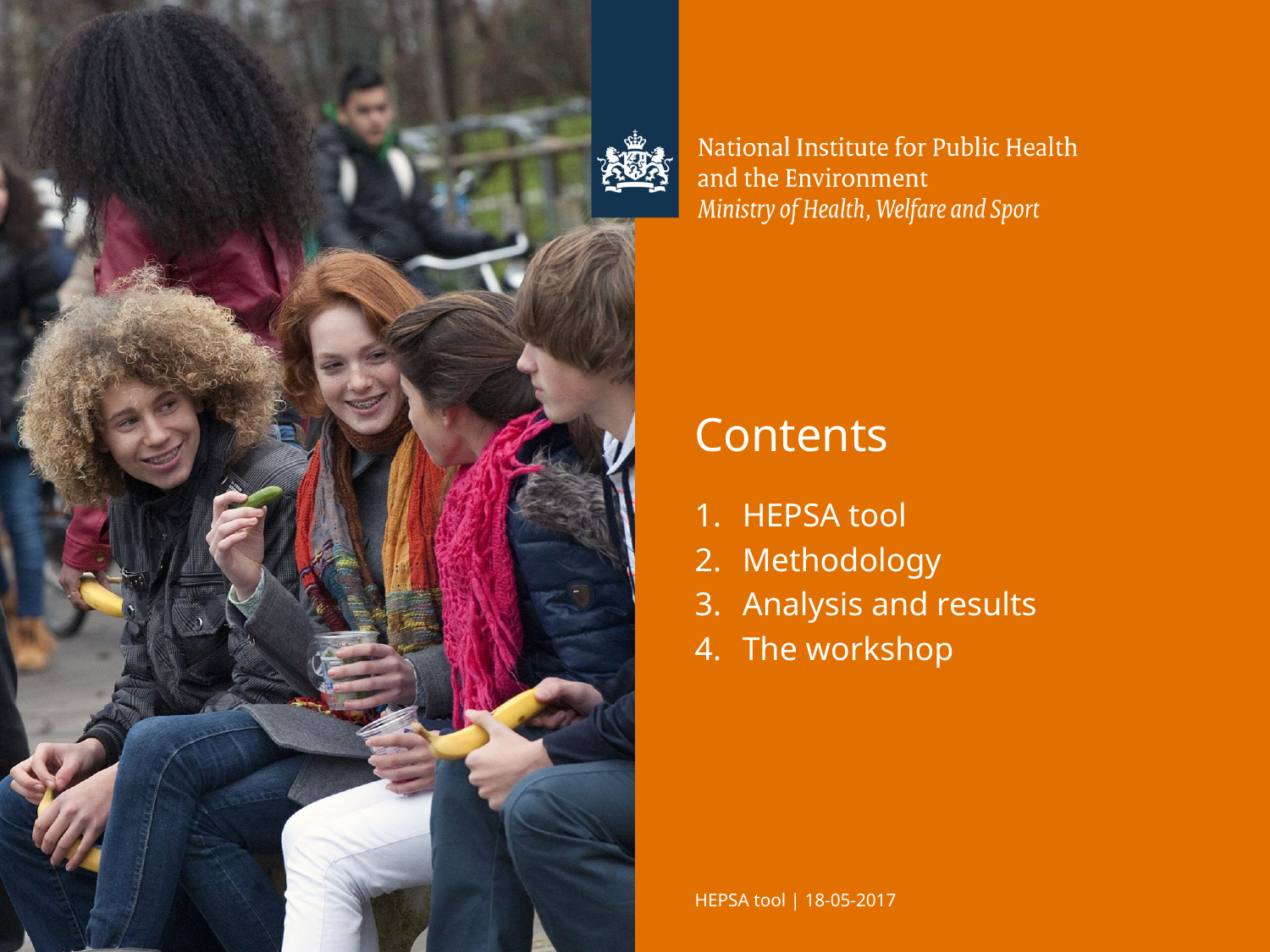

# Contents
HEPSA tool
Methodology
Analysis and results
The workshop
HEPSA tool | 18-05-2017

## Slide 3
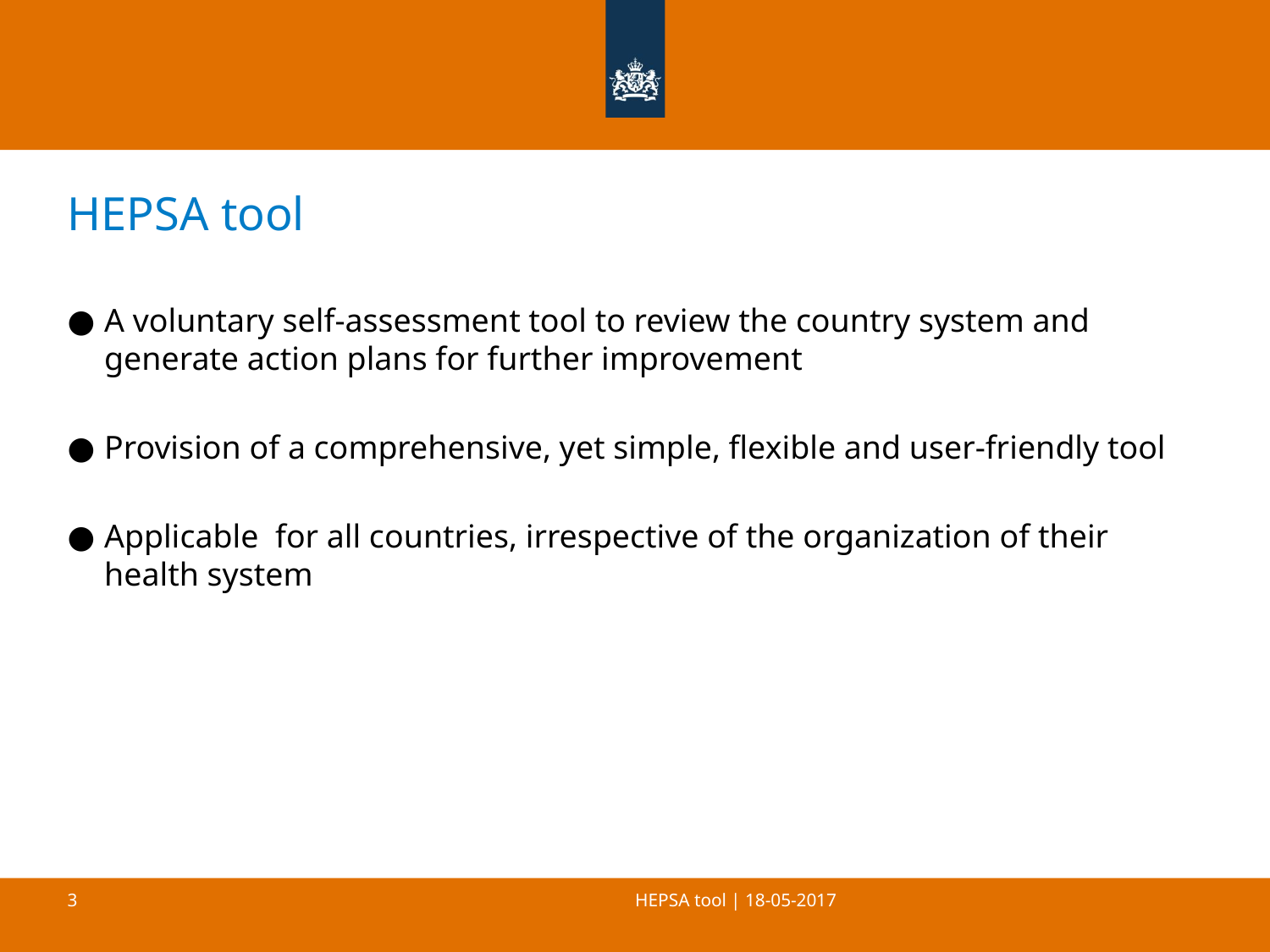

# HEPSA tool
A voluntary self-assessment tool to review the country system and generate action plans for further improvement
Provision of a comprehensive, yet simple, flexible and user-friendly tool
Applicable for all countries, irrespective of the organization of their health system
HEPSA tool | 18-05-2017
3

## Slide 4
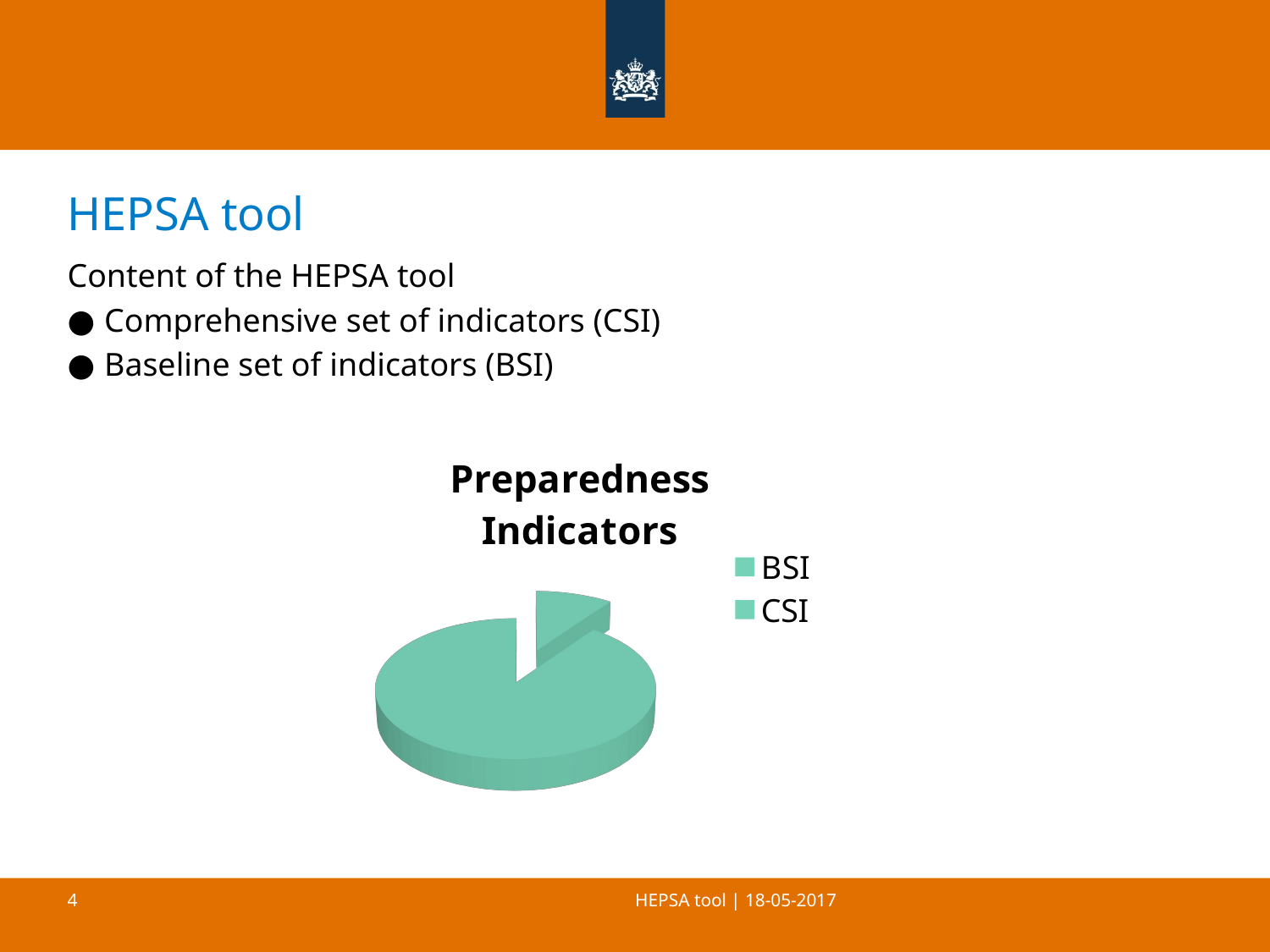

# HEPSA tool
Content of the HEPSA tool
Comprehensive set of indicators (CSI)
Baseline set of indicators (BSI)
[unsupported chart]
HEPSA tool | 18-05-2017
4

## Slide 5
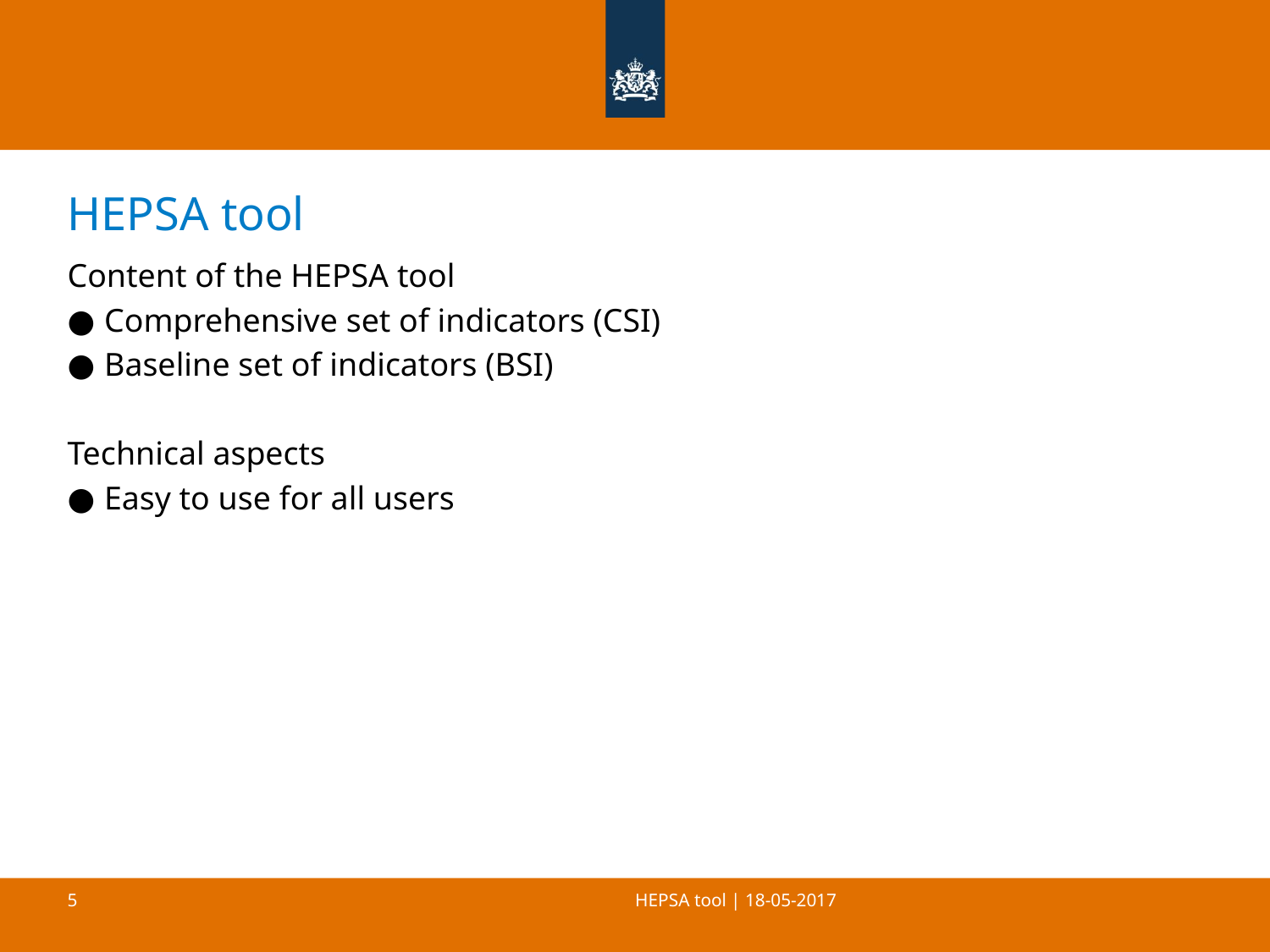

# HEPSA tool
Content of the HEPSA tool
Comprehensive set of indicators (CSI)
Baseline set of indicators (BSI)
Technical aspects
Easy to use for all users
HEPSA tool | 18-05-2017
5

## Slide 6
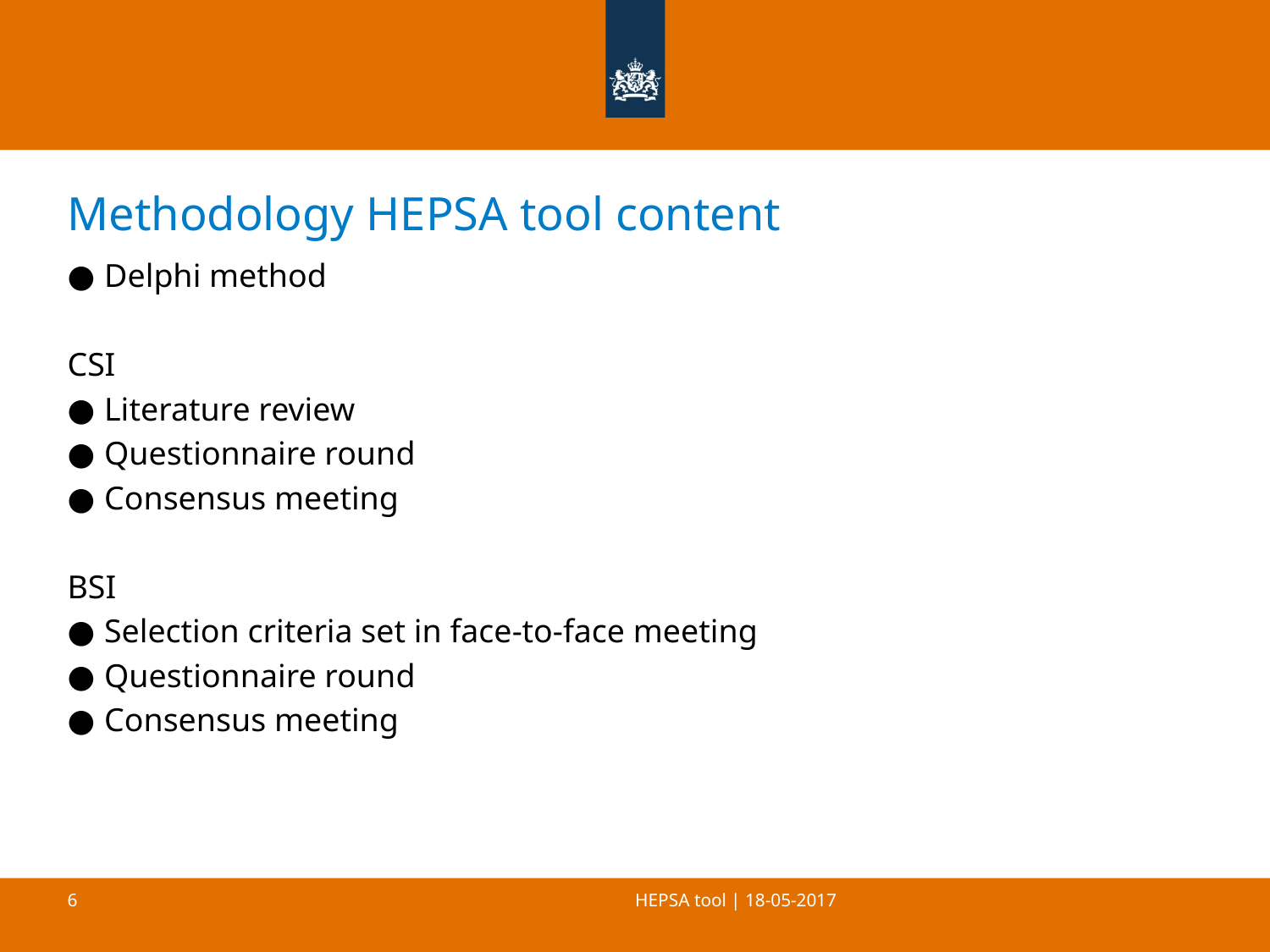

# Methodology HEPSA tool content
Delphi method
CSI
Literature review
Questionnaire round
Consensus meeting
BSI
Selection criteria set in face-to-face meeting
Questionnaire round
Consensus meeting
HEPSA tool | 18-05-2017
6

## Slide 7
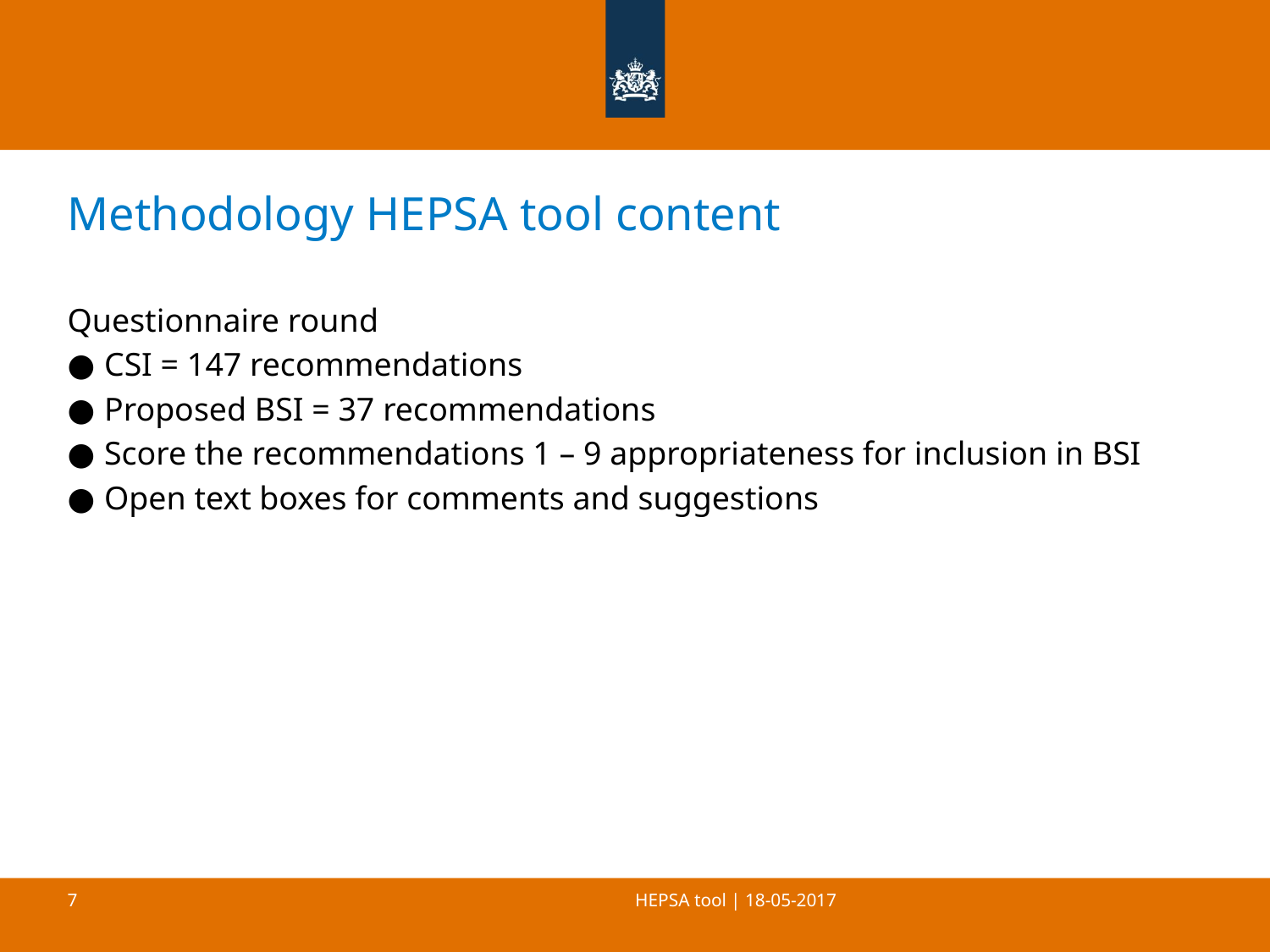

# Methodology HEPSA tool content
Questionnaire round
CSI = 147 recommendations
Proposed BSI = 37 recommendations
Score the recommendations 1 – 9 appropriateness for inclusion in BSI
Open text boxes for comments and suggestions
HEPSA tool | 18-05-2017
7

## Slide 8
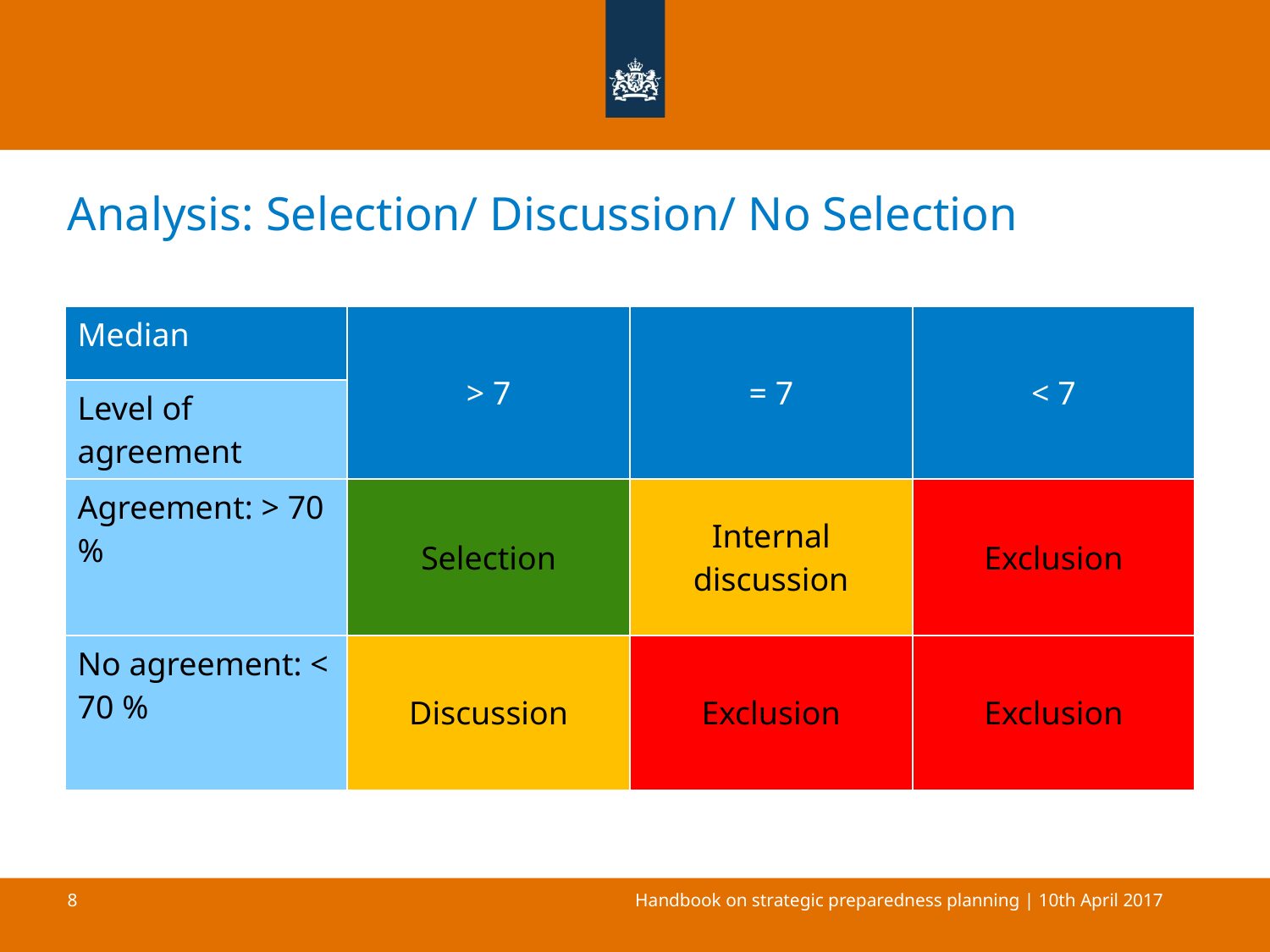

# Analysis: Selection/ Discussion/ No Selection
| Median | > 7 | = 7 | < 7 |
| --- | --- | --- | --- |
| Level of agreement | | | |
| Agreement: > 70 % | Selection | Internal discussion | Exclusion |
| No agreement: < 70 % | Discussion | Exclusion | Exclusion |
Handbook on strategic preparedness planning | 10th April 2017
8

## Slide 9
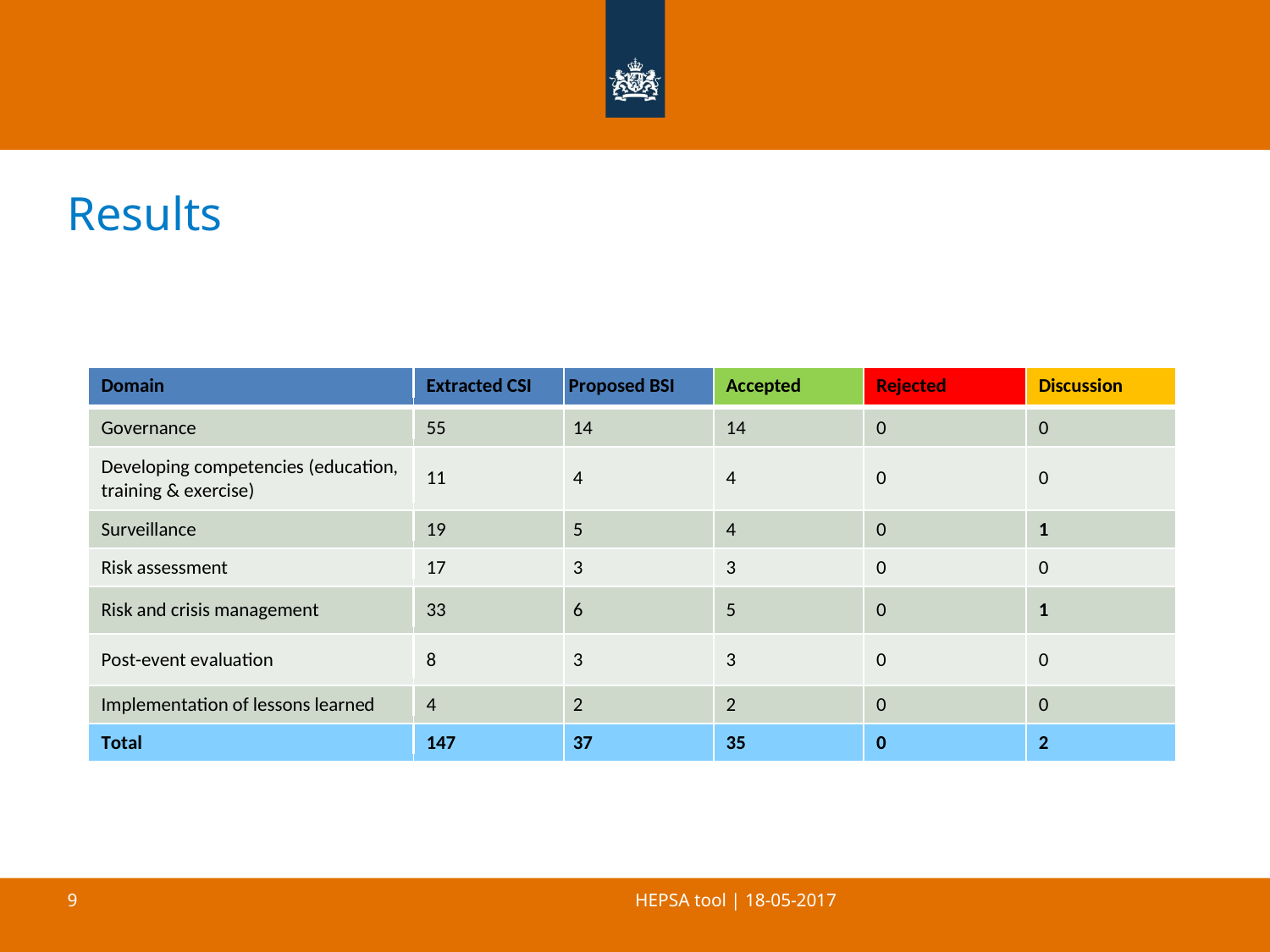

# Results
HEPSA tool | 18-05-2017
9

## Slide 10
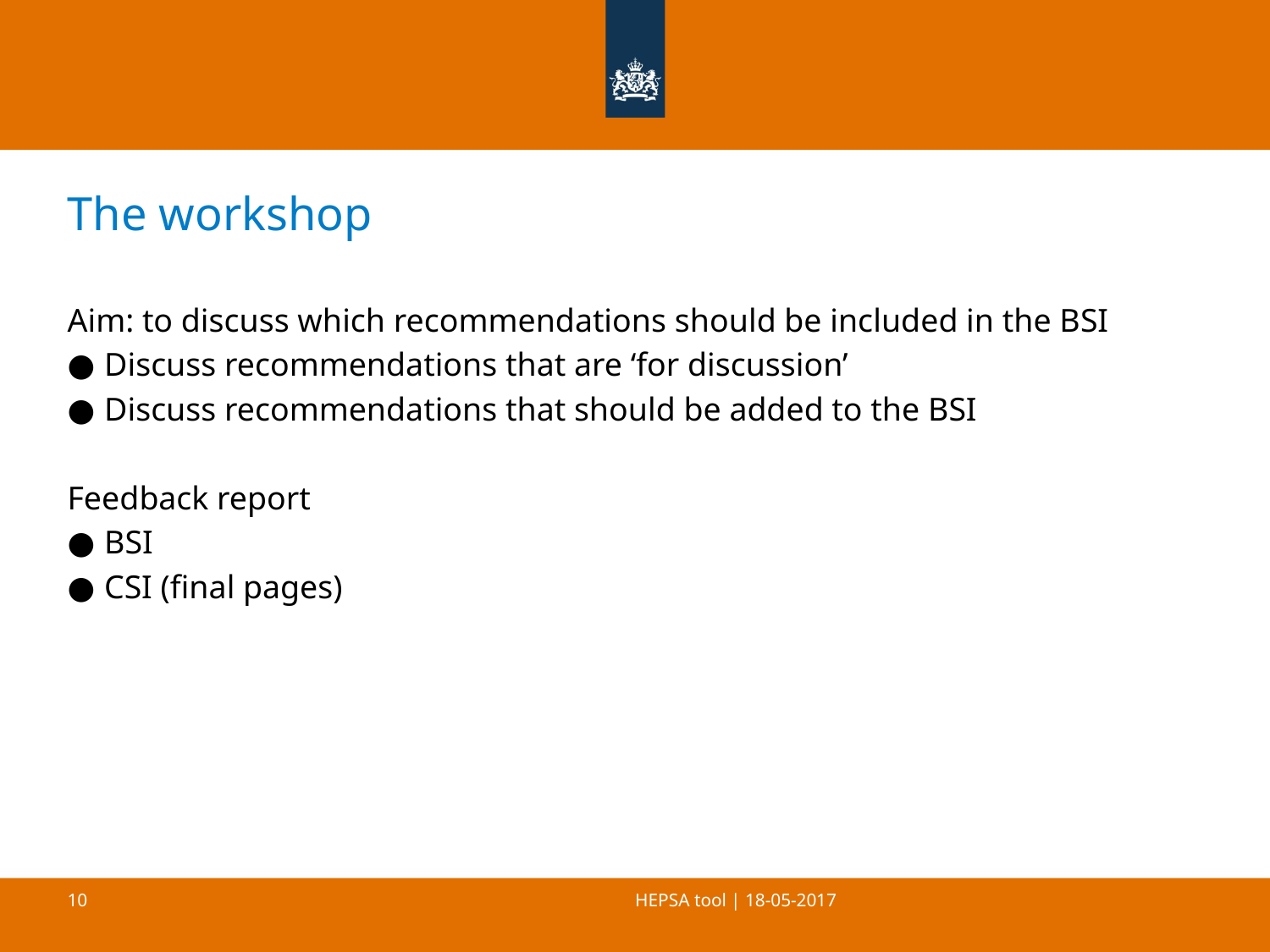

# The workshop
Aim: to discuss which recommendations should be included in the BSI
Discuss recommendations that are ‘for discussion’
Discuss recommendations that should be added to the BSI
Feedback report
BSI
CSI (final pages)
HEPSA tool | 18-05-2017
10

## Slide 11
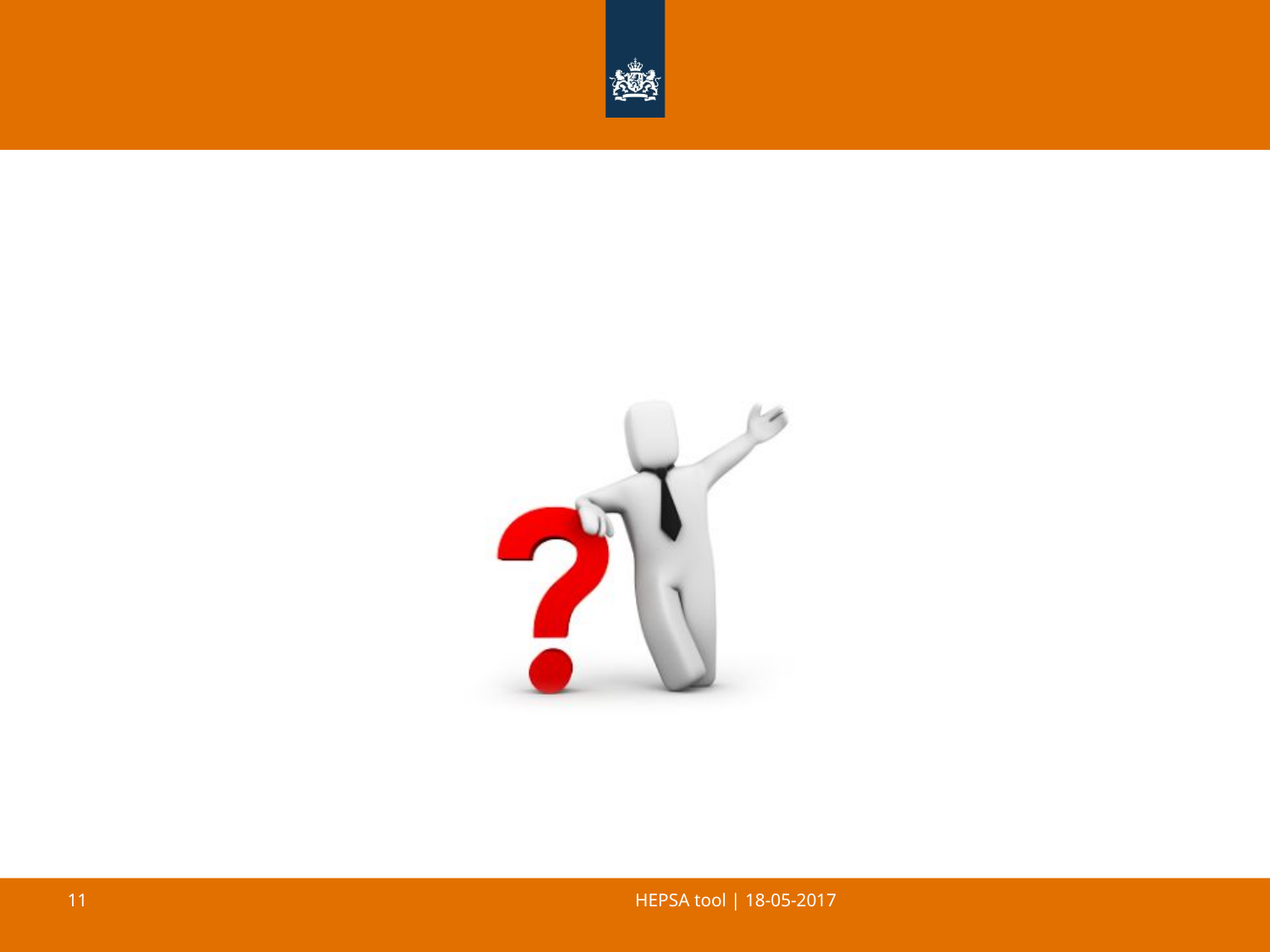

#
HEPSA tool | 18-05-2017
11

## Slide 12
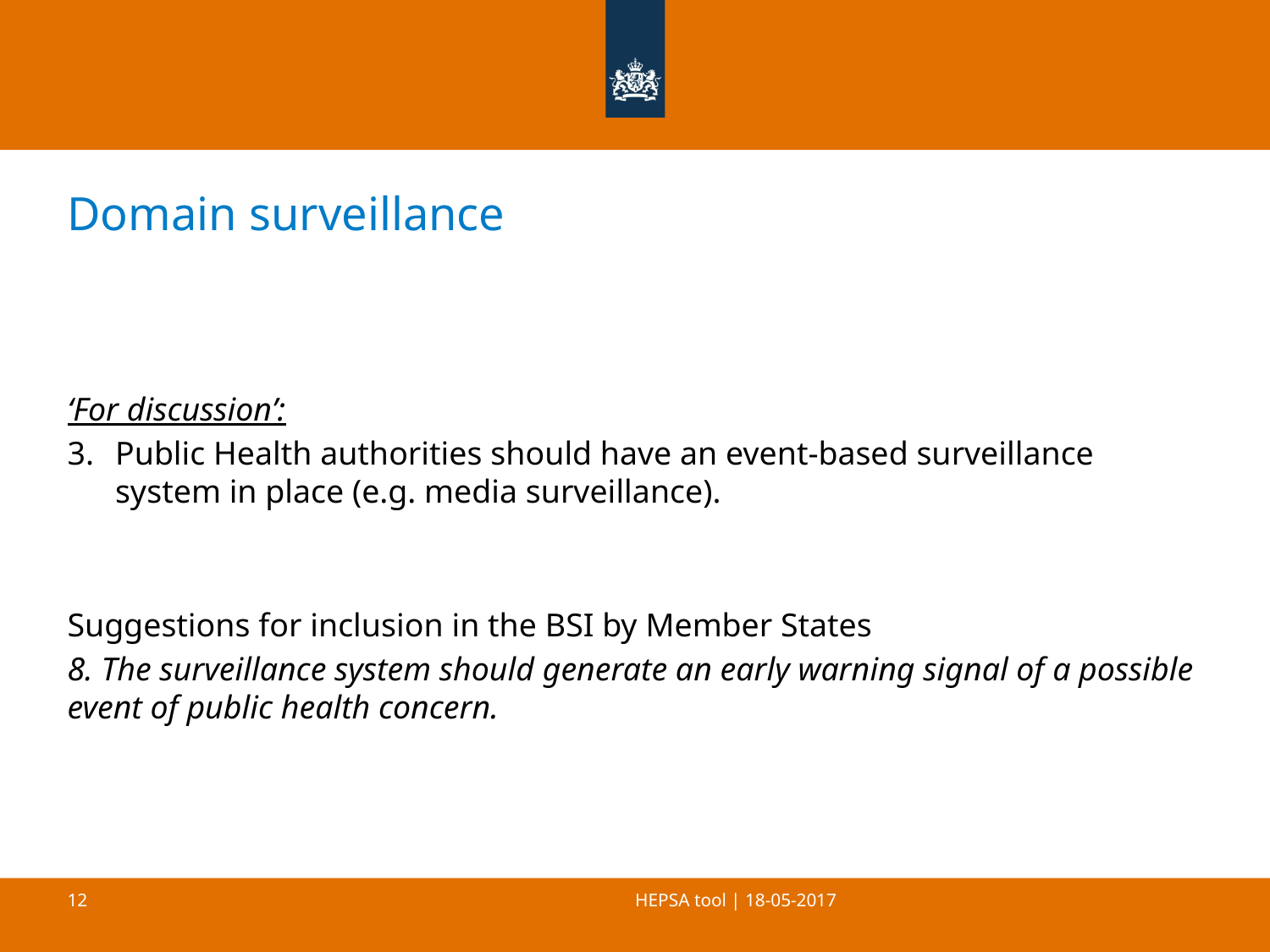

# Domain surveillance
‘For discussion’:
Public Health authorities should have an event-based surveillance system in place (e.g. media surveillance).
Suggestions for inclusion in the BSI by Member States
8. The surveillance system should generate an early warning signal of a possible event of public health concern.
HEPSA tool | 18-05-2017
12

## Slide 13
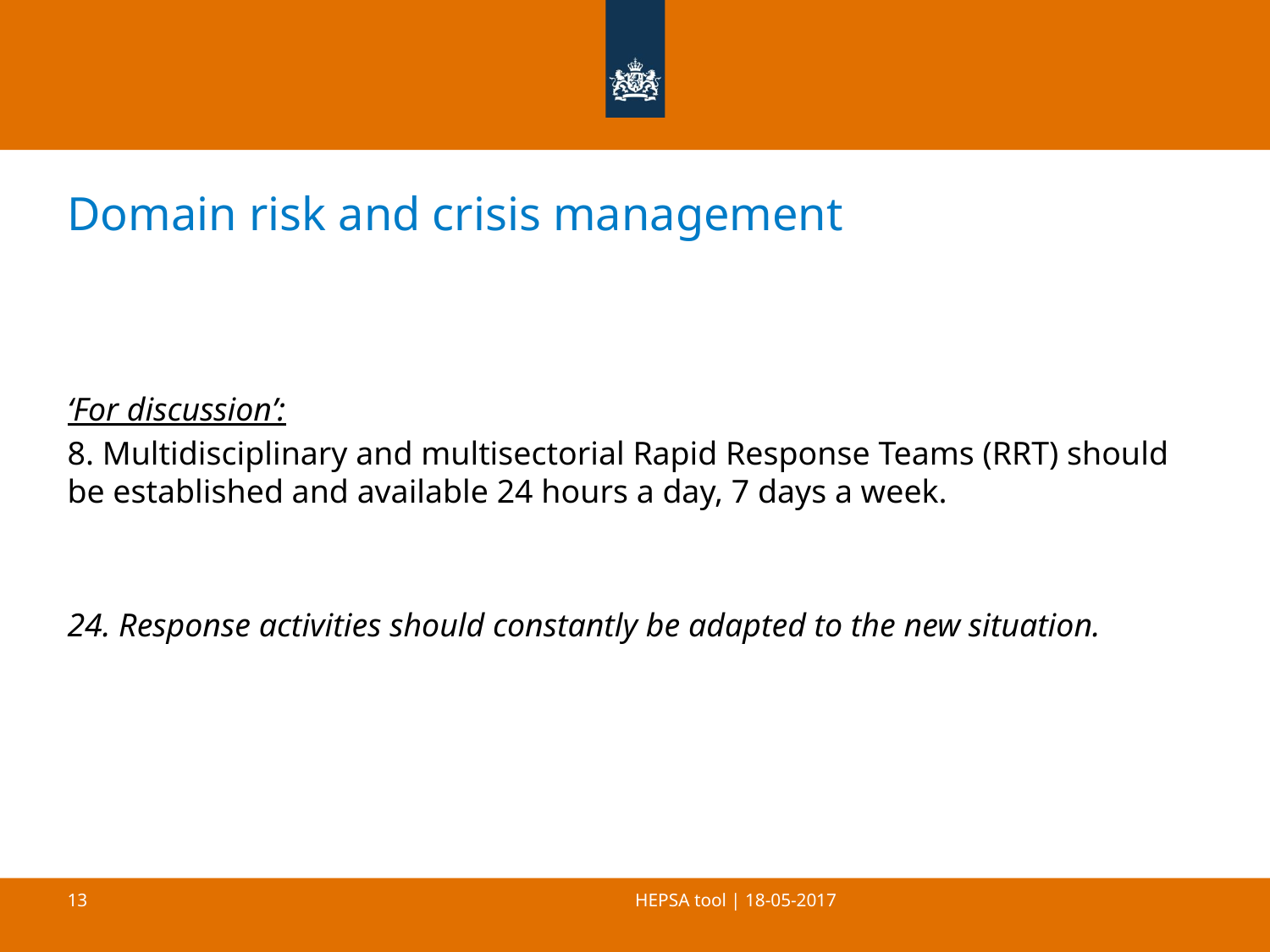

# Domain risk and crisis management
‘For discussion’:
8. Multidisciplinary and multisectorial Rapid Response Teams (RRT) should be established and available 24 hours a day, 7 days a week.
24. Response activities should constantly be adapted to the new situation.
HEPSA tool | 18-05-2017
13

## Slide 14
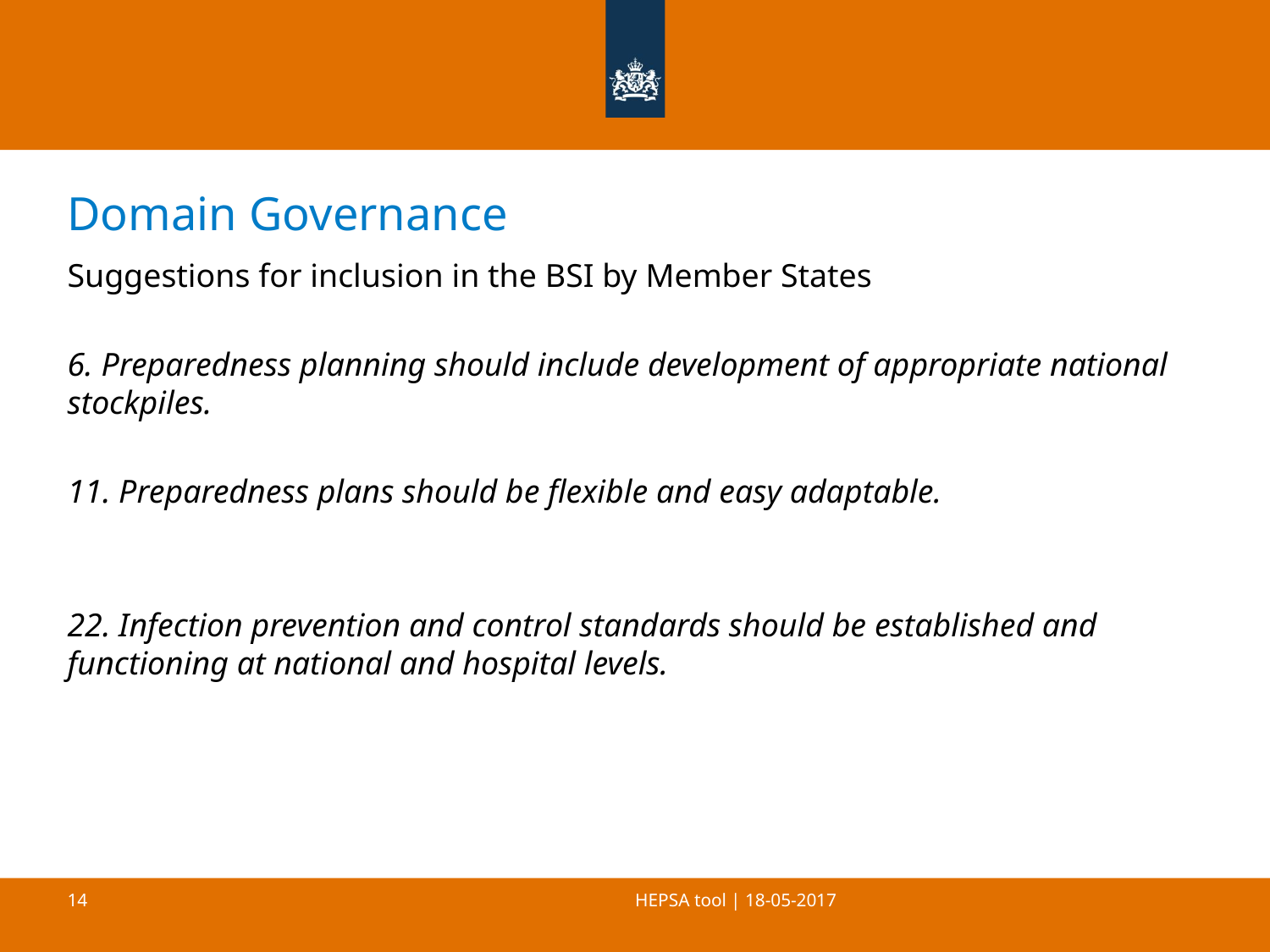

# Domain Governance
Suggestions for inclusion in the BSI by Member States
6. Preparedness planning should include development of appropriate national stockpiles.
11. Preparedness plans should be flexible and easy adaptable.
22. Infection prevention and control standards should be established and functioning at national and hospital levels.
HEPSA tool | 18-05-2017
14

## Slide 15
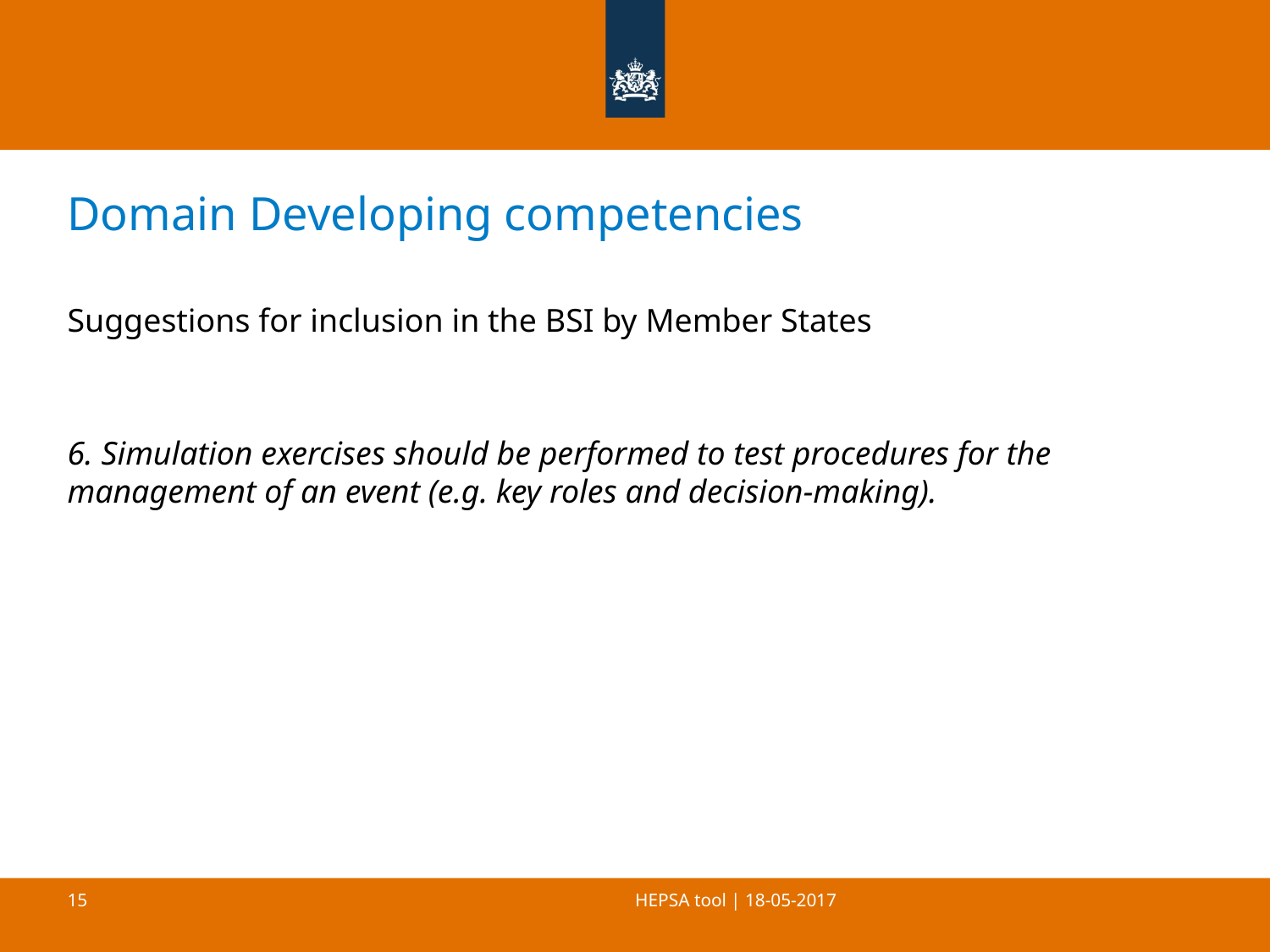

# Domain Developing competencies
Suggestions for inclusion in the BSI by Member States
6. Simulation exercises should be performed to test procedures for the management of an event (e.g. key roles and decision-making).
HEPSA tool | 18-05-2017
15

## Slide 16
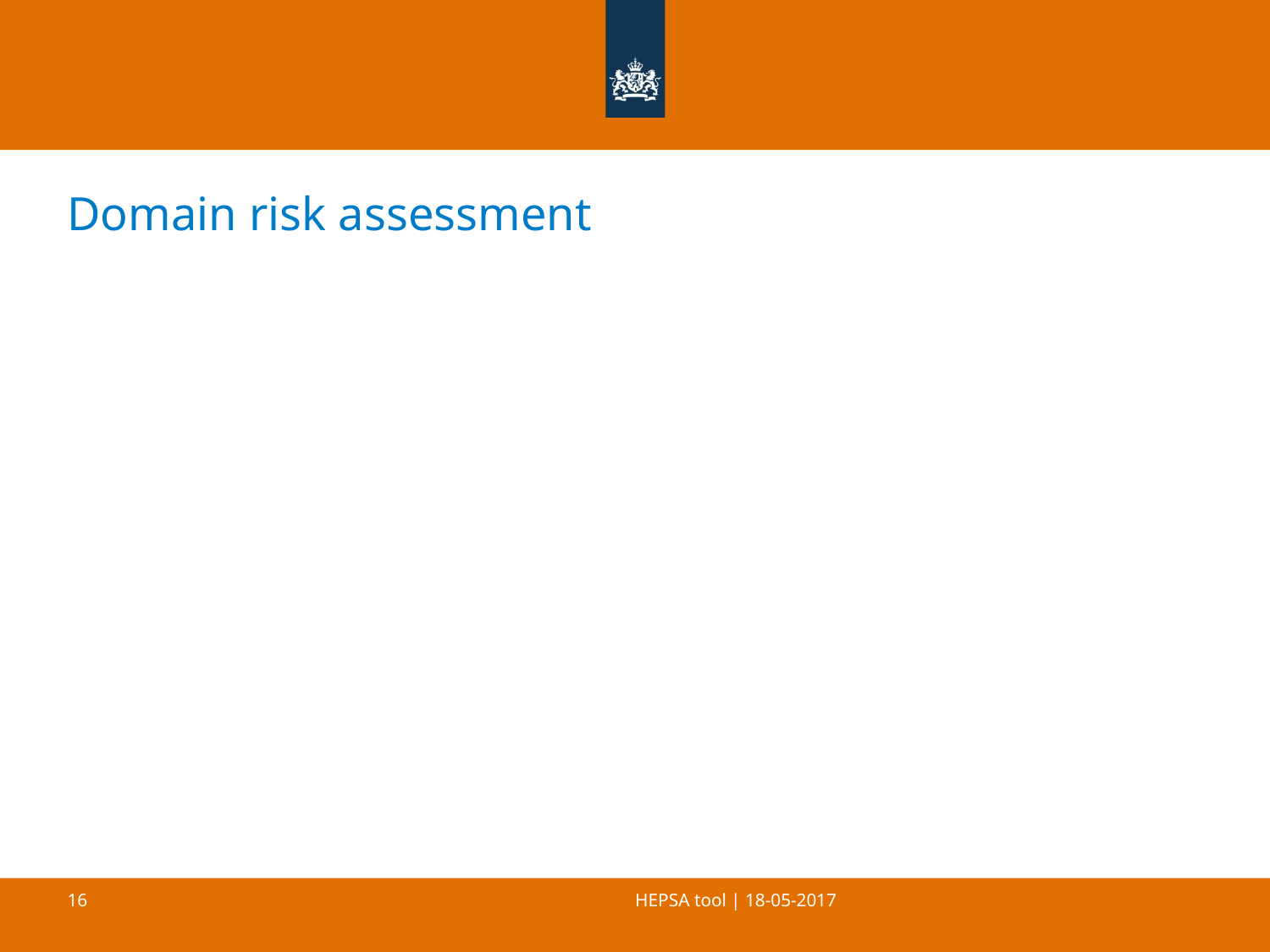

# Domain risk assessment
HEPSA tool | 18-05-2017
16

## Slide 17
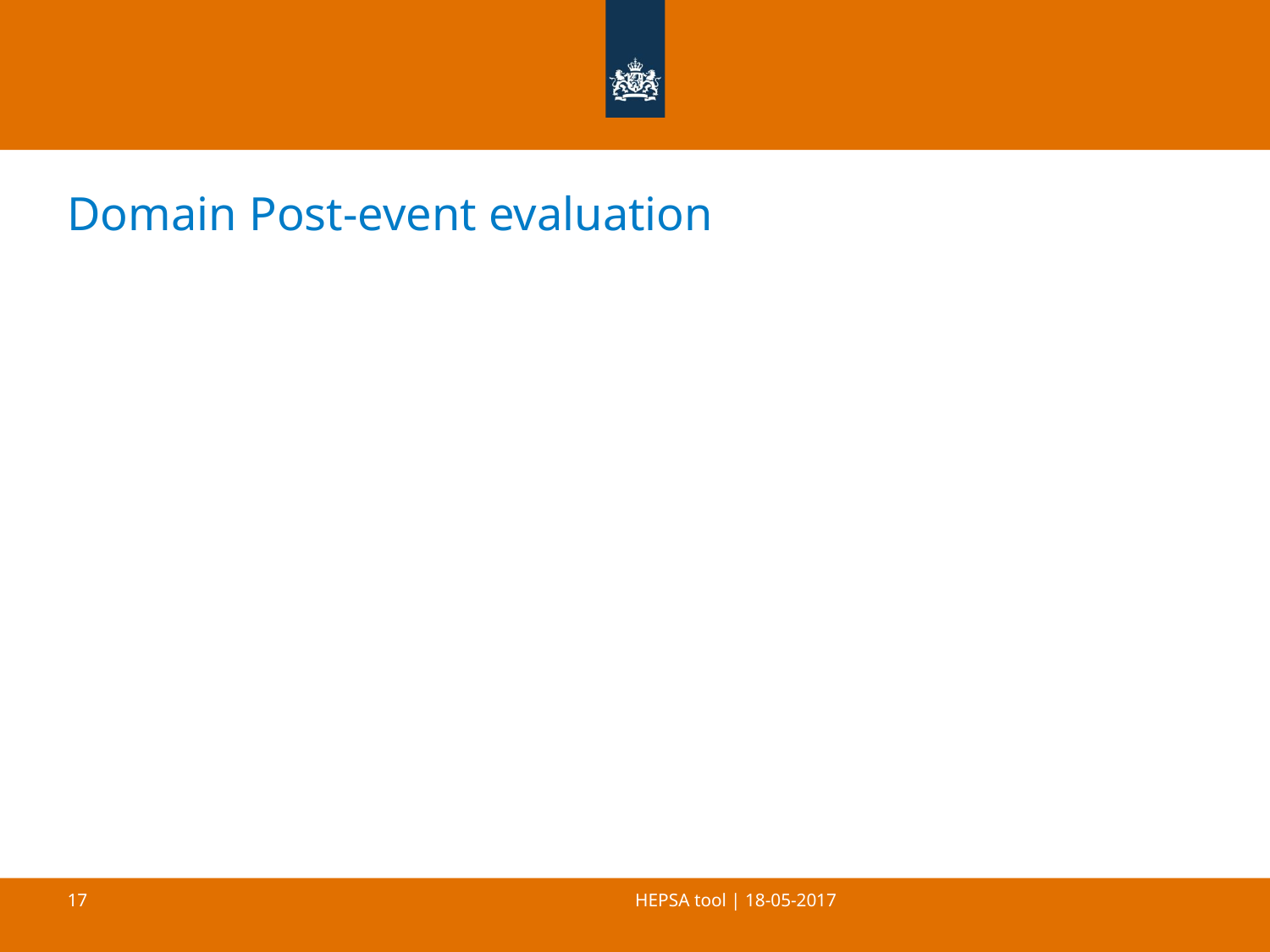

# Domain Post-event evaluation
HEPSA tool | 18-05-2017
17

## Slide 18
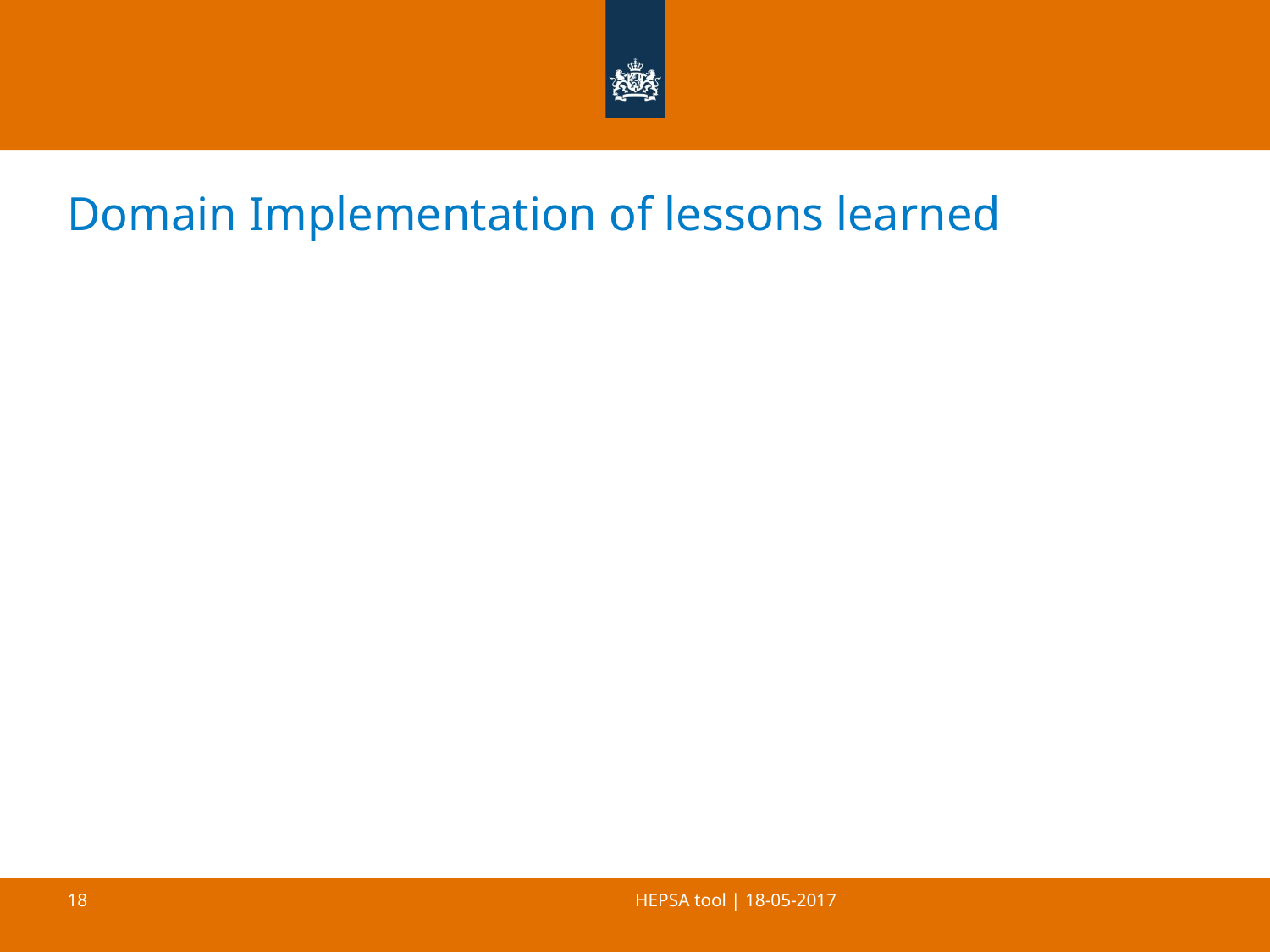

# Domain Implementation of lessons learned
HEPSA tool | 18-05-2017
18
